# Recent synthetic advances in pyridine-based thermotropic mesogens

**DOI:** 10.1039/c9ra04389f

**Published:** 2019-07-26

**Authors:** Deepak Devadiga, T. N. Ahipa

**Affiliations:** Centre for Nano and Material Sciences, Jain University Jain Global Campus, Jakkasandra Post, Ramanagara District Bangalore-562 112 India tn.ahipa@jainuniversity.ac.in

## Abstract

Currently, numerous articles have reported pyridine-based thermotropic mesogens; however, reviews of their synthetic methodologies are rare. Therefore, the present critical review describes the recent synthetic advances in the field of pyridine-based thermotropic mesogens. Also, we discuss the various types of thermotropic mesogens (such as calamitic, discotic, bent-shaped, polycatenar, and polymeric mesogens) consisting of pyridine derivatives and their structure–property relationships.

## Abstract 2

Currently, numerous articles have reported pyridine-based thermotropic mesogens; however, reviews of their synthetic methodologies are rare.

## Graphical abstract

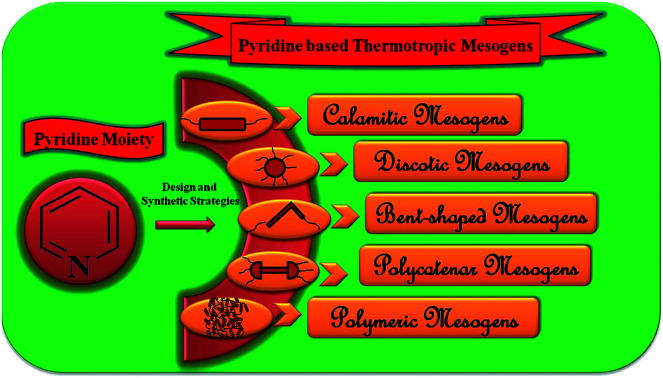

## 1. Introduction

For certain organic compounds, samples do not move directly from the solid to the liquid state upon heating. Instead, they pass through an intermediate state called the liquid crystalline state. The compounds which exhibit this special feature are designated as liquid crystal materials. In general, liquid crystals are calamitic, discotic, polycatenar or bent-shaped organic molecules with terminal groups such as –OR, –COOR, –Cl, and –NO_2_ and bridging groups such as –C=N–, –COO–, –NH–(C=O)–, and –C=C–. In general, liquid crystals are known for their unique characteristics. In fact, these molecules have high order and no or little translational freedom in their solid states. On the other hand, they do not have intrinsic order in the liquid state. The characteristic ordering in the liquid crystal state is between those in the solid and liquid states, *i.e.*, the molecules in the liquid crystal state are almost parallel to each other and can flow like a liquid with translational freedom. Thus, liquid crystals have the fluidity of liquids and the layer ordering of solids.

It is interesting to note that the design and development of various heterocyclic-based thermotropic liquid crystals has gained the interest of researchers due to the stress-free modification of molecular shapes and their excellent ability to impart lateral and/or longitudinal dipoles.^1–3^ In fact, these features have helped further several photochemical and optical applications in areas of material science, such as organic photo-voltaic devices,^4–6^ optical signal processing and storage,^7^ switching ferroelectric materials,^8^ and organic transistors.^9^ In the last few years, a pronounced number of thermotropic liquid crystalline materials with core moieties comprising heterocyclic rings have been designed, synthesized and characterized. These heterocyclic rings consist of electronegative hetero-atoms (such as nitrogen, oxygen, and sulphur), and the occupancy of these electronegative atoms frequently triggers diminished symmetry of the complete molecule and, thereby, develops powerful polar induction.^7^ Highly π-conjugated mesogens bearing heterocycles are showing enhanced use in organic photonics.^10^ In fact, five-membered N-heterocyclic compounds are frequently fabricated in materials science due to their ability to further interact (or hydrogen bond) with metal atoms.^7^ Five-membered N-heterocyclic compounds are capable of building complexes with metals or may exhibit mesogenic natures through hydrogen bond donor–acceptor assemblies *via* specific interactions. Also, some of these compounds can be electron rich or deficient based on the ring nature and, therefore, can be utilized in organic electronic applications. In this context, pyrazoles have been efficiently implemented in the domain of metallo-mesogens; at the same time, triazoles have been utilized to produce supramolecular mesogens.^7^

The use of six-membered N-heterocyclic systems in mesogenic molecules moderately alters their molecular geometry in contrast with their benzene analogs. However, the site of the N-heteroatom has a significant impact on the dispersion forces, the dipole moment, the polarizability of the molecule and, inevitably, on the dielectric nature of the mesogen.^11^ Thus, depending on the heteroatom sites in mesomorphic derivatives, the dielectric anisotropy of mesogens can have negative or positive values. In the case of pyridazine derivatives, they have negative dielectric anisotropy because their dipole moment directions create an angle close to 90° with a long molecular axis; in the case of pyrimidine derivatives, they have positive dielectric anisotropy because their dipole moments are directed along the molecular axis.^3^ However, the presence of a single nitrogen atom in the case of pyridine also alters the photophysical, electrical, and thermal mesogenic behaviours compared to carbocyclic derivatives. Inspired by these unique properties, we focus our present review article on recent advances in the field (such as the design, synthesis, and photophysical and liquid crystal properties) of pyridine-based compounds as our prime subject of interest.

## 2. Pyridine-based molecules as thermotropic mesogens

### 2.1. Pyridine-based calamitic mesogens

Calamitic mesogens are rod-like molecules wherein the length of the molecule is much greater than its width. The rigid core usually consists of aromatic rings (phenyl, biphenyl, *etc.*) and/or even heteroaromatic rings. In addition, certain alicyclic rings (cholesteryl, *trans*-4-cyclo hexyl, *etc.*) can be used. In general, these cores are connected either by covalent bonds or by linkage units (–COO–, –N=N–, –CH=N–). Moreover, these molecules contain terminal alkyl or alkoxy chains; in some cases, one of these chains is a polar group (CN, NCO, NCS, *etc.*). This section summarizes the recently developed synthetic methods for the preparation of different pyridine-based calamitic mesogens and their properties.

**Scheme 1:**
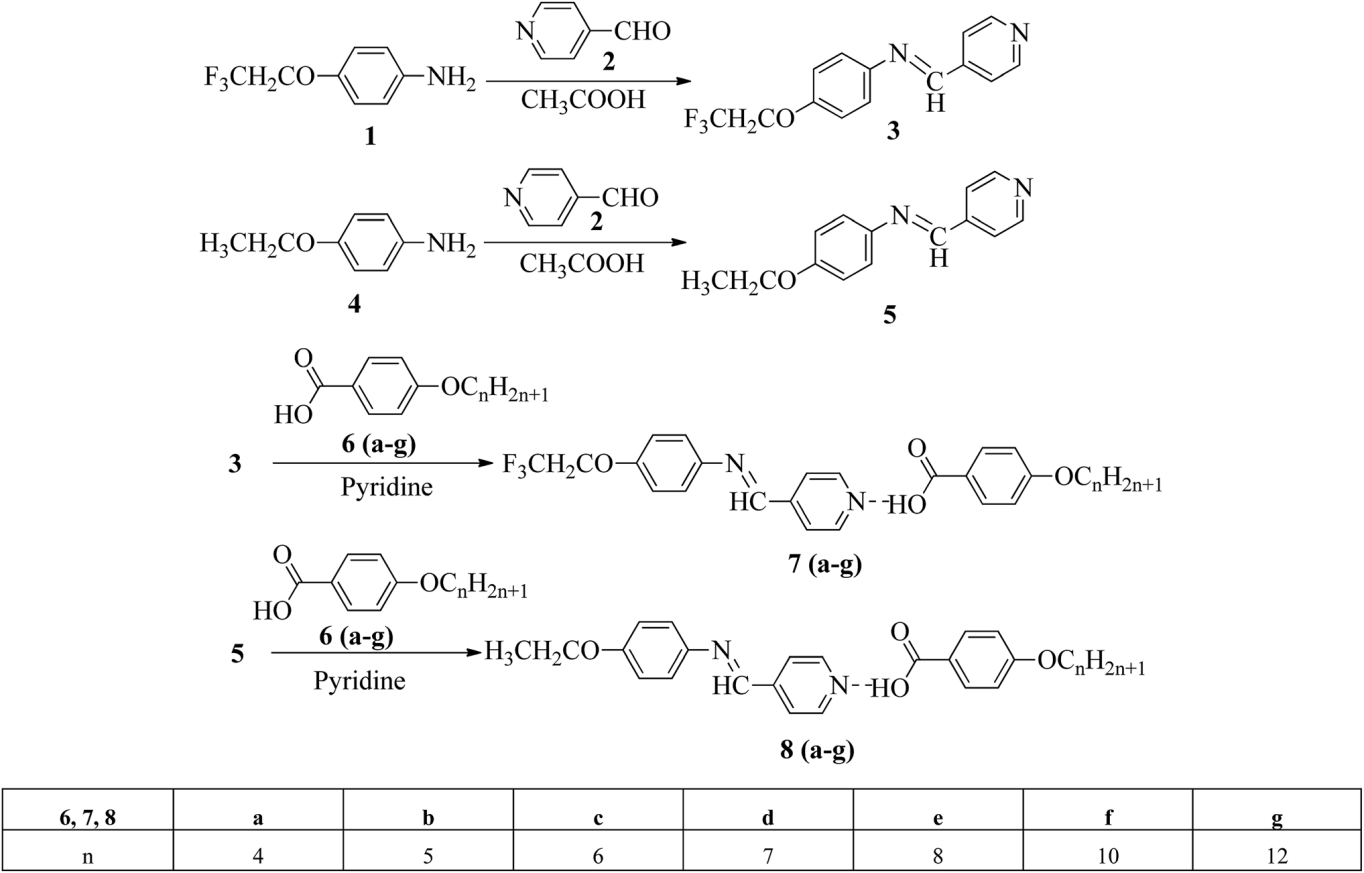
Synthetic routes for 7a–g and 8a–g.

Liu *et al.*^12^ synthesized two proton acceptor molecules bearing Schiff base linkages, *i.e. N*-(pyridin-4-ylmethylene)-4-(2,2,2-trifluoroethoxy)benzenamine (3) and 4-ethoxy-*N*-(pyridin-4-ylmethylene)benzenamine (5). These compounds were synthesized by reacting either 4-(2,2,2-trifluoroethoxy)aniline (1) or 4-ethoxyaniline (4) with pyridine 4-carbaldehyde (2) through condensation reactions. Further, they prepared a series of supramolecular hydrogen bonded liquid crystal complexes (7a–g, 8a–g) using these proton acceptor molecules (3, 5) and 4-(*n*-alkoxy)benzoic acids (6a–g) in pyridine solvent (Scheme 1). The formation of hydrogen bonding between the pyridine derivative and 4-(*n*-alkoxy)benzoic acid was confirmed by comparison of the infrared spectra of the parent compounds and the hydrogen bonded complex. Polarizing optical microscopy (POM) and differential scanning calorimetry (DSC) studies on these hydrogen bonded complexes reveal that the fluorinated analogues have higher clearing temperatures than the non-fluorinated analogues; hence, the authors concluded that there is increased stability and a higher degree of order in the fluorine analogue-based hydrogen bonded complexes due to the presence of trifluoromethyl groups. Further, the fluorinated analogues exhibited smectic A phase and the non-fluorinated analogues showed nematic phase under POM examination; later, these mesophases were further confirmed by X-ray diffraction (XRD) studies. Moreover, the authors checked the stability of the imine bonds in the prepared compounds through hydrolysis reactions using water and ethanol in a 9 : 1 v/v ratio at room temperature. From this study, it was observed that the imine bonds are stable even after 7 days of continuous hydrolysis; the stability of the imine bonds is believed to be due to the existence of conjugation in the molecules.

**Scheme 2:**
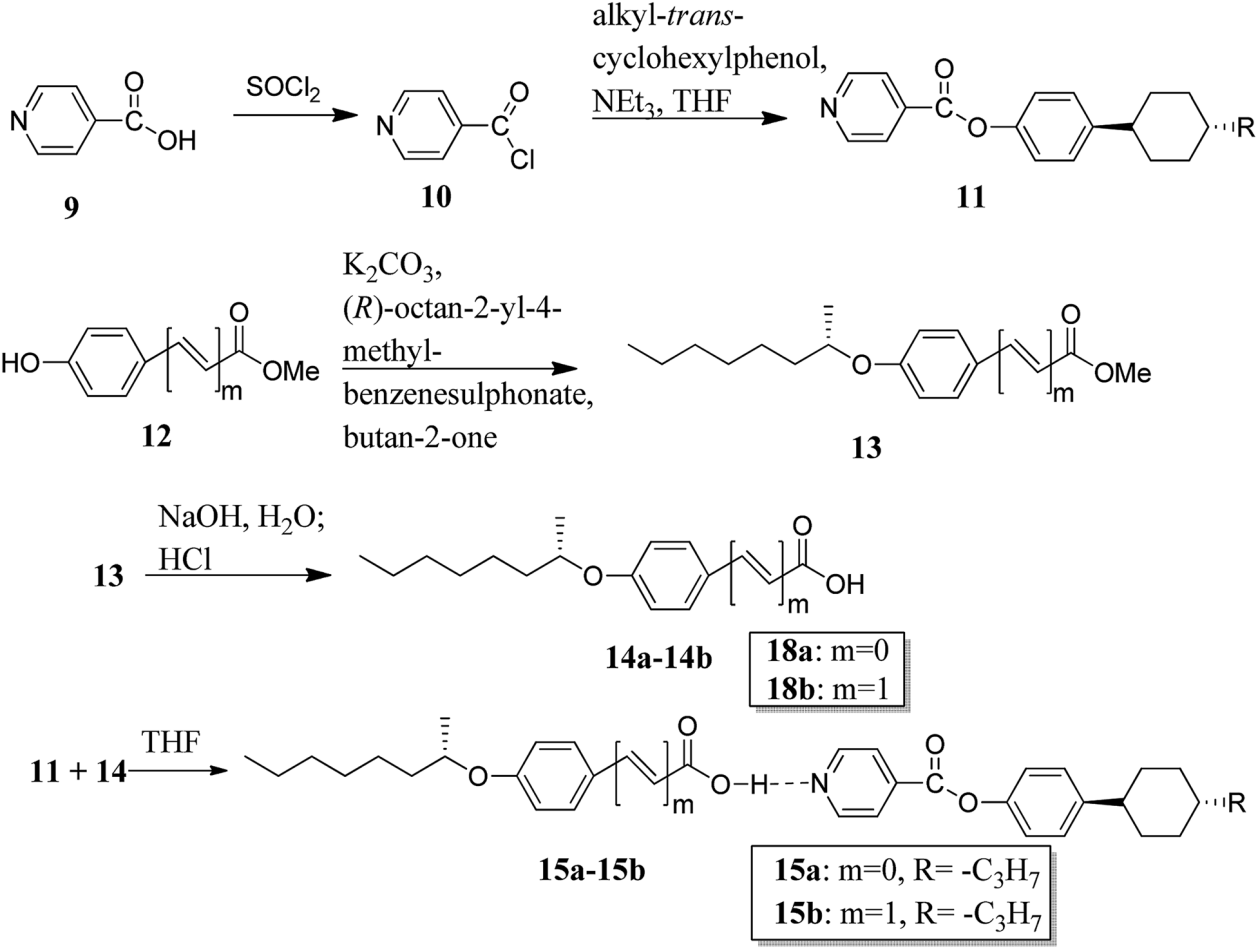
Synthetic routes for the hydrogen bonded liquid crystals 15a and 15b.

Recently, He *et al.*^13^ synthesized a proton acceptor molecule, *i.e.* 4-(4-*trans*-propylcyclohexyl)phenol isonicotinate (11), with an ester linkage, and proton donor molecules, (*S*)-4-(2-octanyloxy)benzoic acid (14a) and (*S*)-4-(2-octanyloxy)cinnamic acid (14b), as shown in Scheme 2. These proton acceptor and donors are non-mesogenic in nature. Further, hydrogen bonded complexes were prepared by dissolving 1 : 1, 1 : 2, and 1 : 3 mole ratios of 11 and 14a or 14b, respectively, in tetrahydrofuran (THF) and heating the resulting mixture to 60 °C for several minutes followed by solvent evaporation; this led to the formation of hydrogen bonded complexes. These hydrogen bonded complexes exhibited liquid crystalline properties. Mesophases such as smectic A (SmA), twisted-grain-boundary A (TGBA*) and chiral nematic phase (N*) were observed in complex 15a, whereas complex 15b exhibited blue phase (BP*) in addition to the mesophases of complex 15a. Hydrogen bonded complex 15b in a mole ratio of 1 : 2 generated BP* mesophase. Further, the BP* phase range was extended to 10 °C during the cooling cycle. Density functional theory (DFT) studies showed that the flexible cinnamic-acid-derived liquid crystals have a bent shape; thus, they concluded that the flexible nature of the compounds plays a positive role in the stabilization of BPs and, thus, these complexes can be used in applications of photonic crystals and optoelectronics.

**Scheme 3:**
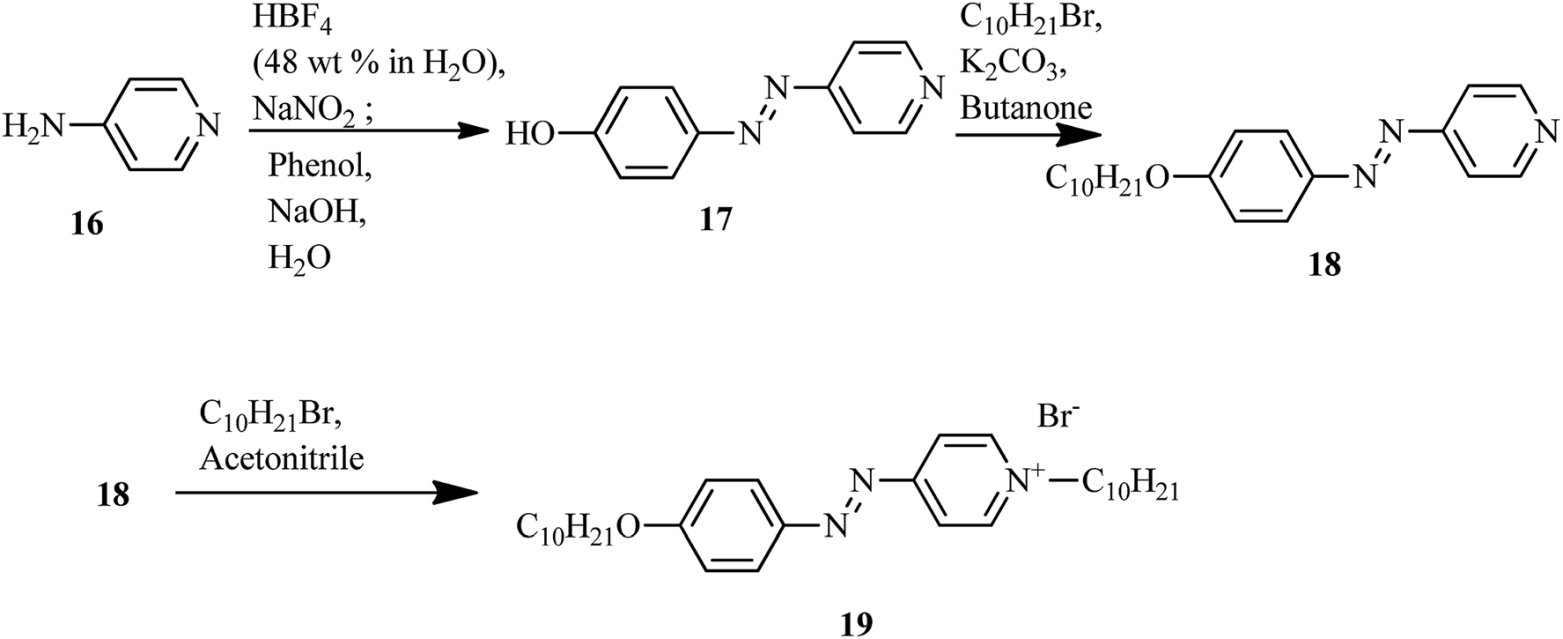
Synthetic route of the ionic liquid crystal 19.

The first report on ionic liquid crystals with amphotropic behavior for the development of electrochemical biosensors was prepared by Zapp *et al.*^14^ The synthetic scheme involves the diazotization of 4-aminopyridine (16) using tetrafluoroboric acid solution (HBF_4_) (48 wt% in water) and NaNO_2_. This yields the corresponding diazonium tetrafluoroborate salt. Further, the prepared diazonium salt is treated with phenol in a basic aqueous solution through an azo-coupling reaction to afford compound (17); alkylation of the phenolic hydroxyl group with *n*-bromodecane in the presence of base, *i.e.* K_2_CO_3_, resulted in the formation of the long-chained compound (18). Finally, the desired ionic compound, *i.e.* (*E*)-1-decyl-4-[(4 decyloxyphenyl)diazenyl]pyridinium bromide (19), was prepared *via* alkylation of pyridine (Scheme 3). Also, POM and DSC studies revealed that compound 19 exhibits liquid crystalline properties from 117 °C to 134 °C during the heating cycle. While cooling, the compound showed a fan-shaped texture; this was designated as smectic A phase. In general, most of the ionic liquids exhibited smectic A phase. This method of synthesis is fast and simple; also, the target compound is an ionic liquid. Moreover, this compound can find applicability in hospitals and emergency units for the selective recognition of myoglobin in the near future.

**Scheme 4:**
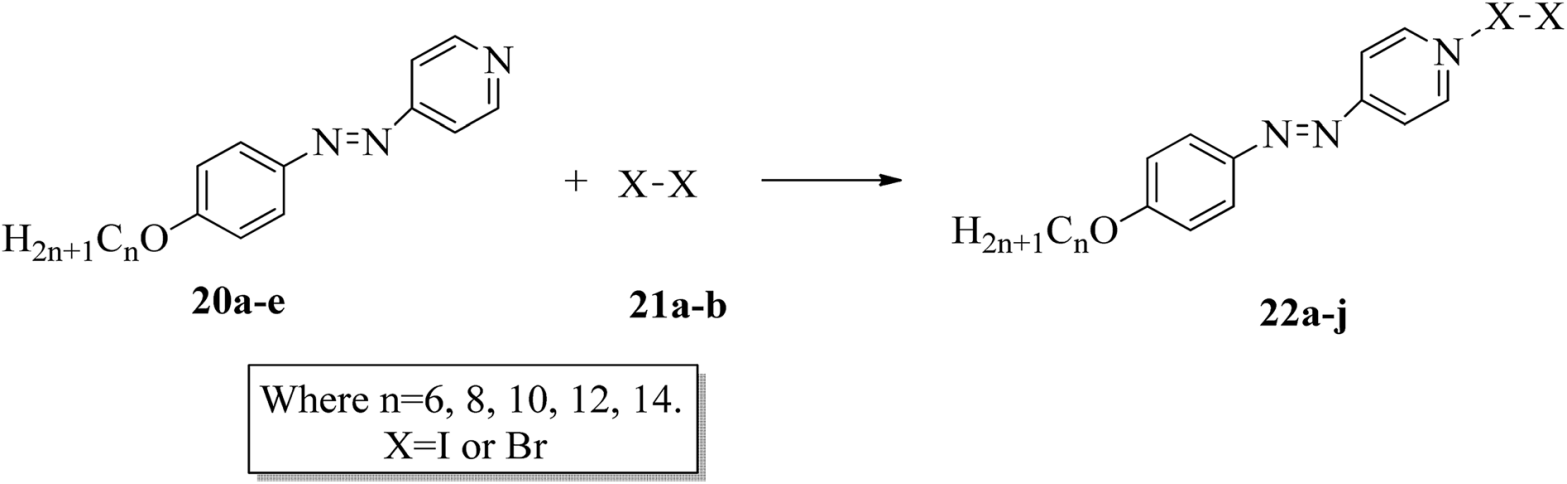
Preparation of halogen bonded liquid crystals (22a–j).

The photoresponsive properties of halogen bonded liquid crystals (22a–j) in their liquid crystalline phases were studied by Chen *et al.*^15^ During the synthesis of 22a–j (Scheme 4), azo pyridines (20a–e) were initially mixed with halogens [iodine (21a) or bromine (21b)]. The required starting materials, *i.e.* azo pyridines 20a–e, were prepared by referring to the report by Mallia *et al.*^16^ Further, their studies revealed that the synthesized azo pyridines (20a–e) are non-mesogenic. However, their halogen bonded complexes showed increased crystallization temperatures, and most of the complexes were liquid crystals. Furthermore, the azo pyridine–iodine complexes with lower chain lengths (*i.e.*22a–b) were non-mesogenic in nature; however, as the chain length increased, smectic A phase appeared, and it was also noted that as the chain length increased in these complexes, the crystallization temperature decreased. However, all the azo pyridine–bromine (22f–j) complexes exhibited mesogenic properties (*i.e.* smectic A phase). Further, they studied the photoresponsive properties of these complexes through *in situ* observation by POM. Upon UV irradiation at 360 nm, the mesogenic texture disappeared and a dark image was formed; hence, a photoinduced phase transition from liquid crystal phase to isotropic phase occurs in the iodine complexes (22a–e) but not in the bromine complexes (22f–j). Hence, the observed phase transitions arise because of photoisomerisation of the prepared complexes from *trans* to *cis* configuration as the bent-shaped isomers destabilize the liquid crystal phases.

**Fig. 1:**
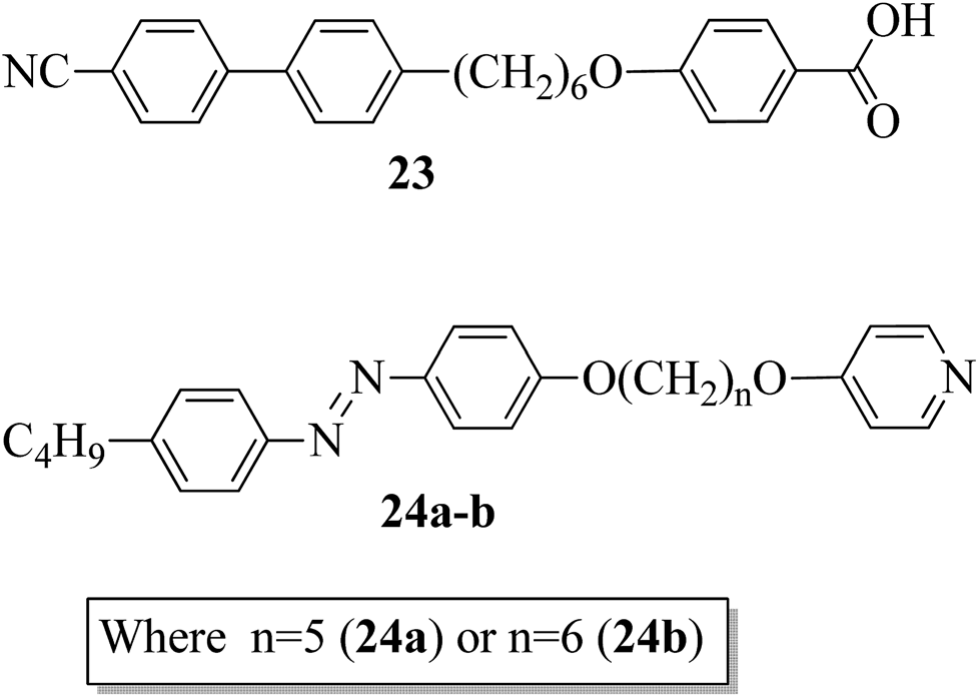
Structures of proton donor 23 and proton acceptors 24a–b.

Paterson *et al.*^17^ prepared mixtures of mesogenic 6-(4′-cyanobiphenyl-4-yl)hexyloxybenzoic acid (23) with non-mesogenic compounds, *i.e.* 1-(4-butylazobenzene-4′-oxy)-5-(4-oxypyridine)pentane (24a) or 1-(4-butylazobenzene-4′-oxy)-6-(4-oxypyridine)hexane (24b), in a 1 : 1 molar ratio. They synthesized the mesogen (23) by referring to the article by Jansze *et al.*,^18^ and two non-mesogenic units, *i.e.*24a–b, were synthesized by referring to the report by Wallage and Imrie.^19^Fig. 1 presents the structures of proton acceptors 24a–b and the structure of proton donor 23. The complexes of mesogenic and non-mesogenic compounds were prepared by dissolving equimolar amounts in chloroform solvent; the solvent was then evaporated and the products were dried. Both mixtures showed enantiotropic nematic phase. However, the evaporated twist-bend nematic phase of 23 was quenched in both complexes. Further, the associated entropy change during the nematic–isotropic transition for the 23/24b mixture and its transition temperature were found to be higher than those of the 23/24a mixture. The complete temperature ranges of the complexes were studied by Fourier transform infrared spectroscopy. It was revealed that this complex is not quantitatively formed but instead involves the 1 : 1 complex, free acid, and both open and cyclic acid dimers; hence, free 24a and 24b molecules exist in their prepared complexes. In all these mixtures, the molecules (average) exist in linear shapes, which helps to increase the elastic constant; hence, the stability of the twisted-bend nematic phases decreases.

**Fig. 2:**
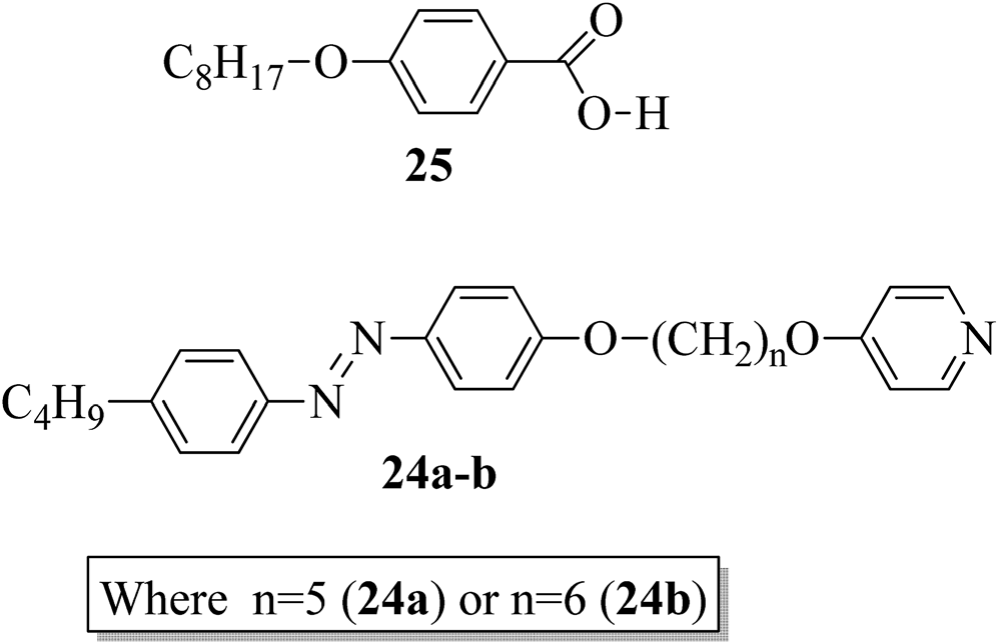
Structures of proton acceptors 24a and b and the structure of proton donor 25.

In addition, Martínez-Felipe and Imrie^20^ prepared binary mixtures by reacting mesogenic 4-octyloxybenzoic acid (25) with either 1-(4-butylazobenzene-4′-oxy)-6-(4-oxypyridine)pentane (24a) or 1-(4-butylazobenzene-4′-oxy)-6-(4-oxypyridine)hexane (24b) in a 1 : 1 molar ratio. For synthesis, the starting materials, *i.e.* the two non-mesogenic units (24a, b), were prepared by referring to the report by Wallage and Imrie;^19^ mesogen 25 was obtained from a commercial source. Fig. 2 presents the structures of proton acceptors 24a–b (non-mesogenic) and the structure of proton donor 25 (mesogenic). The mesogenic and non-mesogenic compounds were initially dissolved in pyridine; the solvent was allowed to evaporate slowly, and the products were later dried in vacuum for 24 h. From the XRD and POM studies, it is revealed that both the equimolar mixtures exhibit smectic A phase; also, in both cases, the nematic and smectic C phases of the mesogen (25) were quenched. However, the even-membered complex was found to be well matched with the smectic A environment; hence, they observed a high transition temperature. Further, Fourier transform infrared spectroscopy revealed that the complexes are not quantitatively formed; instead, the prepared 1 : 1 mixtures contain free acid, both open and cyclic acid dimers and, hence, free 24a and 24b molecules.

**Scheme 5:**
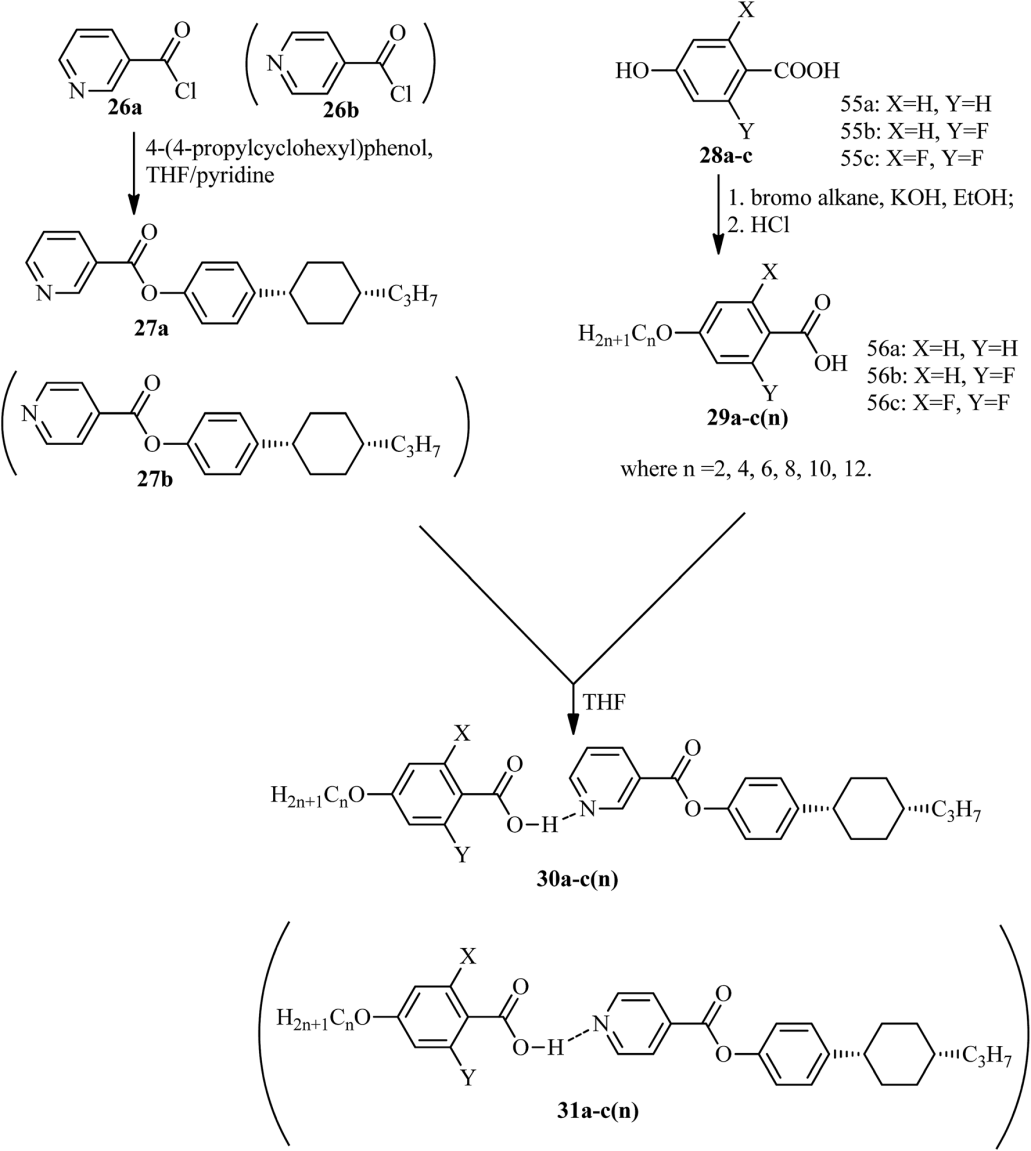
Synthesis of hydrogen bonded complexes 30a–c(*n*) and 31a–c(*n*).

He *et al.*^21^ synthesized nicotinate and isonicotinate derivatives (27a and 27b, respectively) as proton acceptors and studied the effects of bent-shape or calamitic-shape structures on their mesogenic behavior. During the synthesis, nicotinic acid and DMF as solvent were combined and stirred. To this, thionyl chloride (SOCl_2_) was added slowly at room temperature, and the reaction mixture was slowly stirred for about 24 h. After completion of the reaction, the excess SOCl_2_ was removed in vacuum at 40 °C. The resulting crude product was collected by filtration and washed with petroleum ether; the crude product was later dried in vacuum at room temperature to yield nicotinylchloride hydrochloride (26a). Immediately, it was taken in anhydrous THF; to this, 4-(4-propylcyclohexyl)phenol in pyridine was added. This reaction mixture was stirred for 24 h and was then filtered. The filtered product was further dissolved in hexane and activated carbon was added, followed by filtration and evaporation of the solvent to afford the hydrogen bond acceptor 4-(4-propylcyclohexyl)phenylnicotinate (27a). On the other hand, the donor, *i.e.* substituted 4-hydroxybenzoic acid (28a–c), was dissolved in 75% ethanol; then, KOH and KI were added, and the reaction mixture was stirred for 1 h at room temperature, followed by the addition of 1-bromo alkane and refluxing of the reaction mixture for 20 h. Later, the solution was cooled and washed with petroleum ether, and the water layer was acidified using concentrated HCl solution to afford the precipitates of substituted 4-alkoxy-benzoic acid (29a–c(*n*)); then, the precipitates were filtered and recrystallized using 50% v/v ethanol/water. To prepare the hydrogen bonded complexes of the nicotinate derivatives (31a–c(*n*)) and isonicotinate derivatives (30a–c(*n*)), the respective donor and acceptor compounds were dissolved in THF and heated at 40 °C for several minutes, followed by evaporation of the solvent and drying under reduced pressure for 24 h (Scheme 5). Also, the authors studied the effects of the terminal chain length and lateral fluoro-substituents on the prepared complexes. According to the authors, the length to breadth ratio decreased greatly when the structure of the hydrogen bonded liquid crystal changed to bent-shaped from calamitic; this led to decreases in the melting point and clearing point and also narrowed the temperature range of the mesophase. Similarly, substitution of fluorine showed a decreased length to breadth ratio, which led to a narrowed mesophase range.

**Scheme 6:**
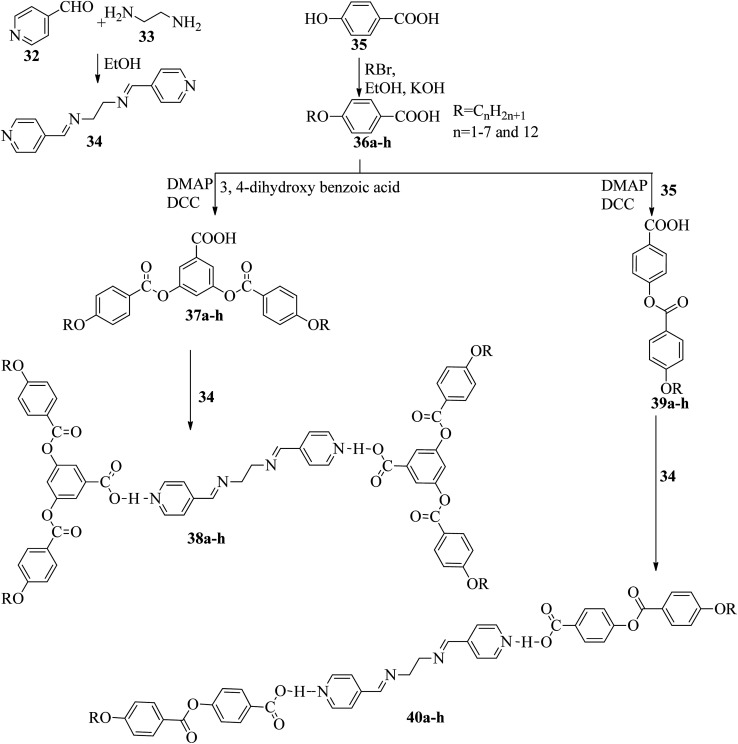
Preparation of hydrogen bonded complexes 38a–h and 40a–h.

Two series of supramolecular hydrogen bonded liquid crystal dimers were prepared by Al-Lami;^22^ the mesogenic properties of the dimers were studied, as well as their structure–property relationships. The synthetic routes for the preparation of the hydrogen bonded complexes are shown in Scheme 6. The author implemented two steps in the reaction route. The first step is the preparation of the hydrogen bond acceptor, *i.e.* α,β-bis(pyridine-4-yl-methylene)ethane-1,2-diamine (34), by refluxing 4-pyridinecarboxaldehyde (32) with 1,2-diaminoethane (33) and a catalytic amount of glacial acetic acid in ethanol solvent for 2 h; the obtained product is filtered and recrystallized using ethanol. The second step is the preparation of the hydrogen bond donors, *i.e.* 4-(4-alkoxybenzoyloxy)benzoic acid (39a–h) and 3,5-bis(4-alkoxybenzoyloxy)benzoic acid (37a–h). These compounds were synthesized through the Steglich esterification method, wherein 4-alkoxy benzoic acid (36a–h) was reacted with 4-hydroxy benzoic acid or 3,5-dihydroxy benzoic acid in dichloromethane solvent followed by addition of the coupling reagent (*i.e.* 1,3-dicyclohexyl carbodiimide (DCC)) and catalyst (*i.e.* 4-dimethylaminopyridine). Further, the reaction mixture was stirred at room temperature for 24 h. Finally, hydrogen bonded dimers 38a–h and 40a–h were prepared by dissolving the intermediates 34 and 37a–h or 39a–h in pyridine solvent, followed by evaporation of the solvent under various conditions for several days. First, the 40a–h series exhibited nematic phase for shorter chain length compounds, *i.e.*40a–b (*n* = 2, 3), and smectic A phase was noted for longer chain length compounds, *i.e.*40c–h (*n* = 4 to 8 and 12). This is due to the increased ratio of lateral to terminal attraction between the molecules with increasing chain length. As the chain length increased, the probability of layer arrangement during the melting process (crystal–liquid crystalline transition) also increased due to the weakened terminal attractions. In contrast, the second series of dimers (38a–h) exhibited nematic phase; this is due to the presence of four terminal alkoxy chains which were attached to the terminal phenyl ring but posed a smaller length/width ratio. Also, it was found that even-numbered chain lengths have higher transition temperatures than odd-numbered chain lengths. Further, their clearing temperatures and melting points were found to gradually decrease as the flexible alkoxy chain length increased, in which even-numbered members had slightly higher values. Furthermore, the decreased melting point and isotropic temperature were due to the increased terminal chain length; also, the number of possible conformations increased, which resulted in distortion of the cylindrical shapes of the mesogens.

**Scheme 7:**
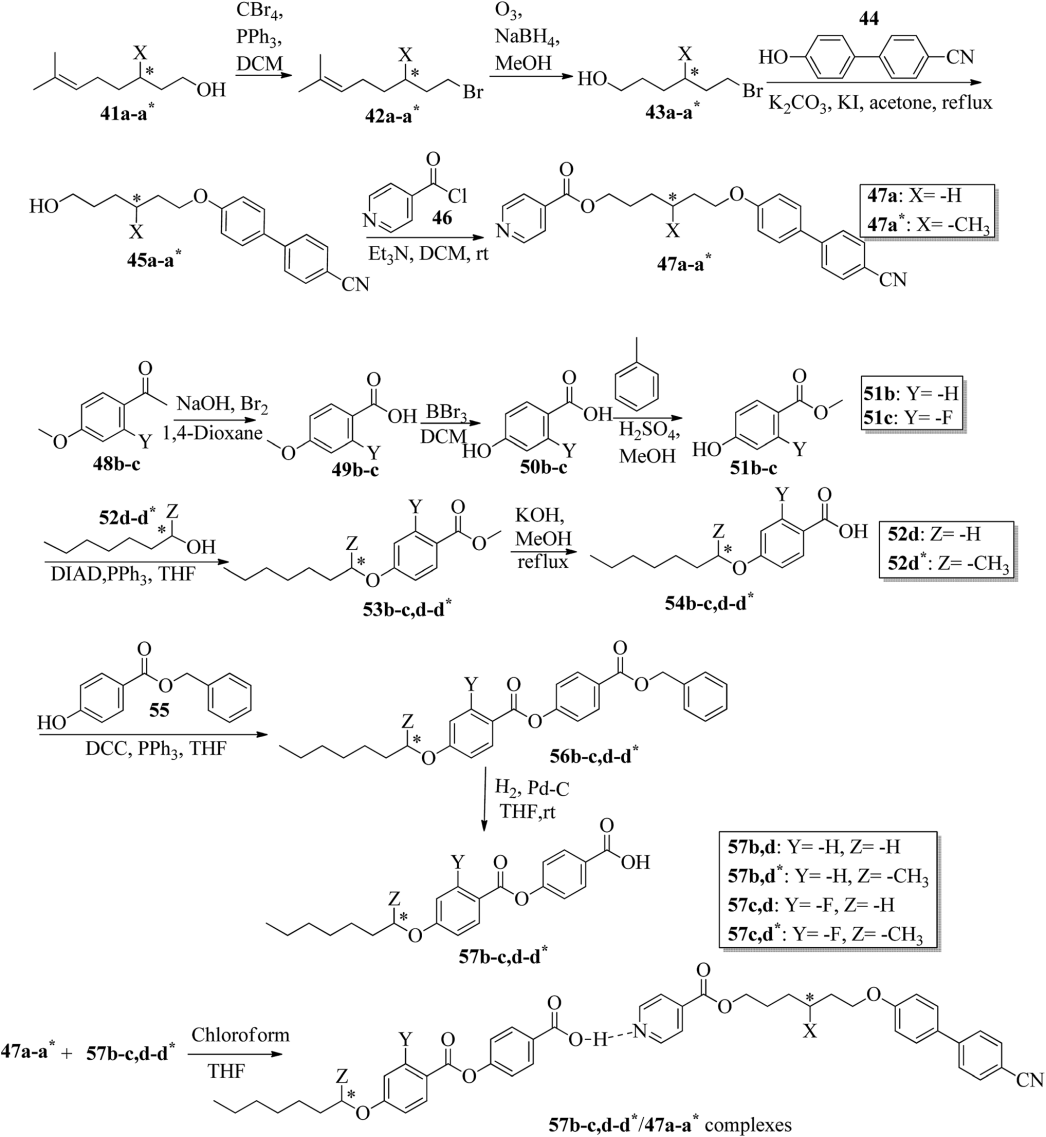
Synthetic route of the 57b-c,d-d*/47a-a* complexes.

Wei *et al.*^23^ prepared a series of asymmetric hydrogen bonded complexes 57b-c,d-d*/47a-a** and also prepared complexes with different molar ratios of proton donors (57b-c,d-d*) and proton acceptors (47a-a*). Further, they studied their mesomorphic properties and compared them with their analogous covalent diads reported by Wei *et al.*^24^Scheme 7 shows the synthetic route for the preparation of the hydrogen bonded complexes. Hydrogen bond donors 57b-c,d-d* were synthesized by stirring 56b-c,d-d* and 10% Pd/C catalyst in THF solvent under hydrogen atmosphere at room temperature overnight. After completion of the reaction, the catalyst was removed by filtration through Celite and washed thoroughly with THF. Later, the solvents were evaporated under reduced pressure. On the other hand, the hydrogen bond acceptors were prepared by dissolving isonicotinoyl chloride hydrochloride (46), compounds 45a-a* and triethylamine in dichloromethane (DCM) and stirring the reaction mixture at room temperature for 8 h under nitrogen. After completion of the reaction, the solvent was extracted with DCM and removed under reduced pressure to afford 47a-a*. Finally, the hydrogen bonded complexes were prepared by mixing appropriate molar ratios of hydrogen acceptors (47a-a*) and hydrogen donors (57b-c,d-d*) in THF solvent, followed by slow solvent evaporation. Further, the hydrogen bonded complexes 57c,d*/47a* (1 : 1 mol) exhibited wide ranges of blue phase (6.0 °C) which became even wider (13.2 °C) as the mole ratio changed to 3 : 1 due to the excess acid; dimers 57c,d* acted as chiral dopants. Comparing the blue phases of these complexes with the previously reported analogous covalent diads showed that the control of the blue phase majorly depends on the bent angle; if the bent angle lies between 132.1° and 152.9° in the molecular structure, it prefers to show blue phase. Because of the unsuitable bent angles in the 57b,d/47a* complexes (162.0°) and the covalent diads 57b,d–47a* (126.5°), they did not show blue phase. Further, complexes 57b,d/47a (1 : 1 mol) and 57b,d/47a* (1 : 1 mol) have higher transition temperatures than complexes 57c,d/47a (1 : 1 mol) and 57c,d/47a* (1 : 1 mol), respectively, because the lateral fluoro substituent is larger than hydrogen. The large size of the lateral fluoro substituent and chiral center causes smaller π–π interactions to occur in the mesogens; thus, the smectic A phase range was decreased by enlarging the N or N* phase range. In addition, these complexes failed to show blue phases because of their smaller biaxial ratios and larger bent angles.

**Fig. 3:**
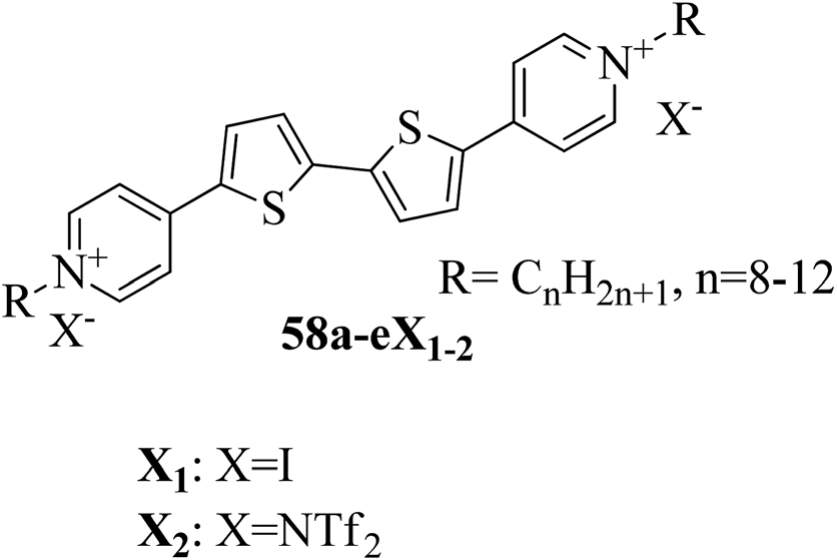
Structure of ionic liquid crystals 58a-eX_1-2_.

Cospito *et al.*^25^ prepared a series of thienoviologens, *i.e.* 4,4′-(2,2′-bithiophene-5,5′-diyl)bis(1-alkylpridinium)X_2_, where X = I (58a-eX_1_) or NTf_2_, *i.e.* (bis(tri-fuoromethylsulfonyl)imide) (58a-eX_2_), and *m* = 8 to 12. The compounds (58a-eX_1_) were synthesized by suspending 5,5′-bis-(4-pyridyl)-2,2′-bithiophene in anhydrous chloroform under nitrogen atmosphere. To this stirred suspension, iodoalkane was added; the mixture was heated to 80 °C for 24 h, then cooled. The solvent was removed under vacuum and diethyl ether was added; then, the suspension was filtered, and the residue was washed with diethyl ether. Lithium triflimide was added to a stirred solution of viologen iodide in methanol and stirred for 15 h at room temperature; then, the solvent was removed, and the resulting mixture was added to water. The solid suspension was filtered and washed with water to afford 58a-eX_2_. Fig. 3 presents the structures of the ionic liquid crystals 58a-eX_1-2_. Further, the study on this series revealed that when the chain length was small (*m* = 8), both series does not exhibited any mesophases; as the chain length increased, the series exhibited mesogenic properties. Comparing the mesomorphic properties of 58a-eX_1_ and 58a-eX_2_ indicated that 58a-eX_1_ have higher clearing points than 58a-eX_2_. Moreover, compounds 58a-eX_2_ have lower crystal-to-mesophase transition temperatures than 58a-eX_1_. These differences are mainly due to the larger size of NTf. Further, studies of the 58a-eX_2_ series indicated that the columnar arrangement was stable at lower chain lengths and lower temperatures, while calamitic behavior was seen at higher chain lengths and higher temperatures. This is mainly because the longer chains destabilize the dimers, leading to calamitic behavior. After the first heating scan of 58eX_2_, the columnar phase was lost because of the larger steric hindrance due to the longer chains. Studies also revealed that the transition temperature to the isotropic liquid state increased with increasing alkyl chain length. The mesophase range increased from 12 °C to 65 °C with increasing alkyl chain length. 58a-eX_2_ exhibited ionic liquid crystalline properties and multifunctional properties; they are also electro-chromic and exhibit strong fluorescence behaviour. Further, all the compounds are electro-active with n-type character, and their LUMO energy levels are very close to those of other organic n-type semiconductors. For all these reasons, these compounds have applicability in organic electronic applications as electron-acceptor materials.

**Fig. 4:**
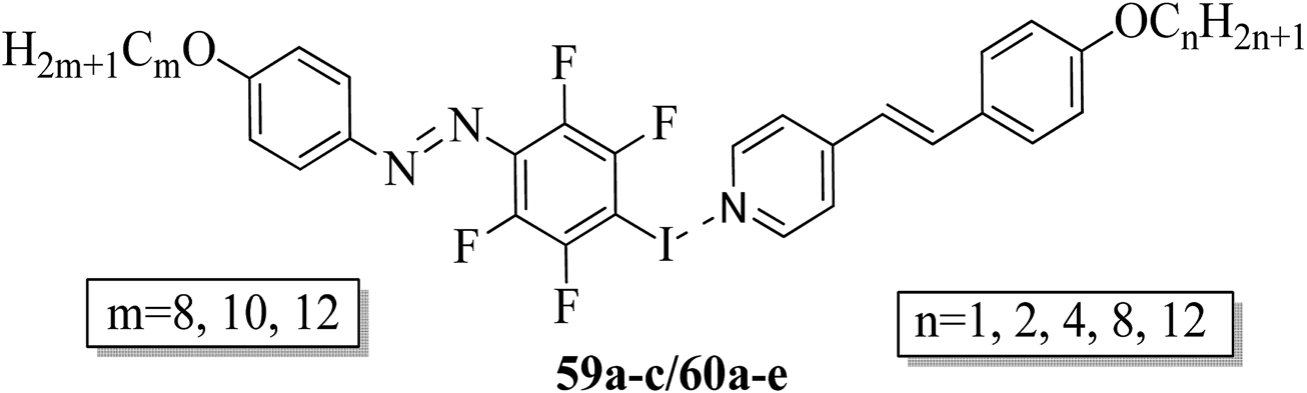
Halogen bonded complexes 59a–c/60a–e.

Fernandez-Palacio *et al.*^26^ synthesized halogen bonded fluorinated supramolecular liquid crystals and also studied their light-responsive properties. This was the first report on light-induced and reversible crystal-to-isotropic phase transitions in supramolecular liquid crystals. They synthesized alkoxystilbazoles by referring to a previous literature report (*i.e.* Bruce *et al.*^27^), while the iodoperfluorinated azobenzenes were synthesized by diazotization of 4-iodo-2,3,5,6-tetrafluoroaniline using nitrosonium tetrafluoroborate (NOBF_4_) at −30 °C. After 1 h, the mixture was stirred at room temperature with the relative alkoxybenzene in nitrogen atmosphere using acetonitrile solvent. Further, the mixture was extracted using dichloromethane (CH_2_Cl_2_) and the solvent was removed under reduced pressure. In the case of the halogen bonded fluorinated supramolecular complexes, fluorine serves to increase the lifetime of the *cis*-form of the azobenzene units and also strengthens the non-covalent interactions with the stilbazole molecules. Fig. 4 presents the prepared halogen bonded complexes (59a–c/60a–e). Further, enantiotropic liquid crystal phases were observed in all the prepared complexes except complexes 59c/60e, which were monotropic in nature. In all the other complexes, nematic phase was commonly observed, while smectic A phases were identified only in the complexes of the longest alkyl chains when attached at both ends. Most of these complexes exhibited broad temperature ranges of nematic phase and mesophase ranges greater than 30 °C. They also noted that the onset temperatures for the mesophases can be suppressed by preparing mixtures of several complexes compared to single supramolecular entities. For example, they mixed 59c with 60b and 60e in a 1.0 : 0.5 : 0.5 molar ratio and noted that the mixture exhibited a crystal-to-nematic transition at 79.5 °C, which was 10 °C lower than that of pure 59c/60b. Also, these complexes undergo fast and reversible transitions from isothermal liquid crystal-to-isotropic phase upon irradiation of UV light; also, they are photoresponsive complexes. They also undergo reversible crystal-to-isotropic transitions in addition to liquid crystal-to-isotropic phase transitions under UV light irradiation. Moreover, a clear reversible crystal-to-isotropic transition was observed within 30 s during 395 nm UV light irradiation. When the irradiation was ceased, recrystallization was observed in *ca.* 3 min through partial nematic phase; finally, homogeneous crystalline phase was formed. The partial nematic phase and significant delay were observed because of photoisomerization rather than a photothermal effect. Light-induced changes in birefringence, absorption, and optical scattering analysis revealed that less than 4% of the mesogenic units in the *cis*-form were sufficient to cause the full liquid crystal-to-isotropic phase transition. Finally, it was concluded that these complexes have potential uses in the fabrication of supramolecular actuators and tunable photonic devices.

**Scheme 8:**
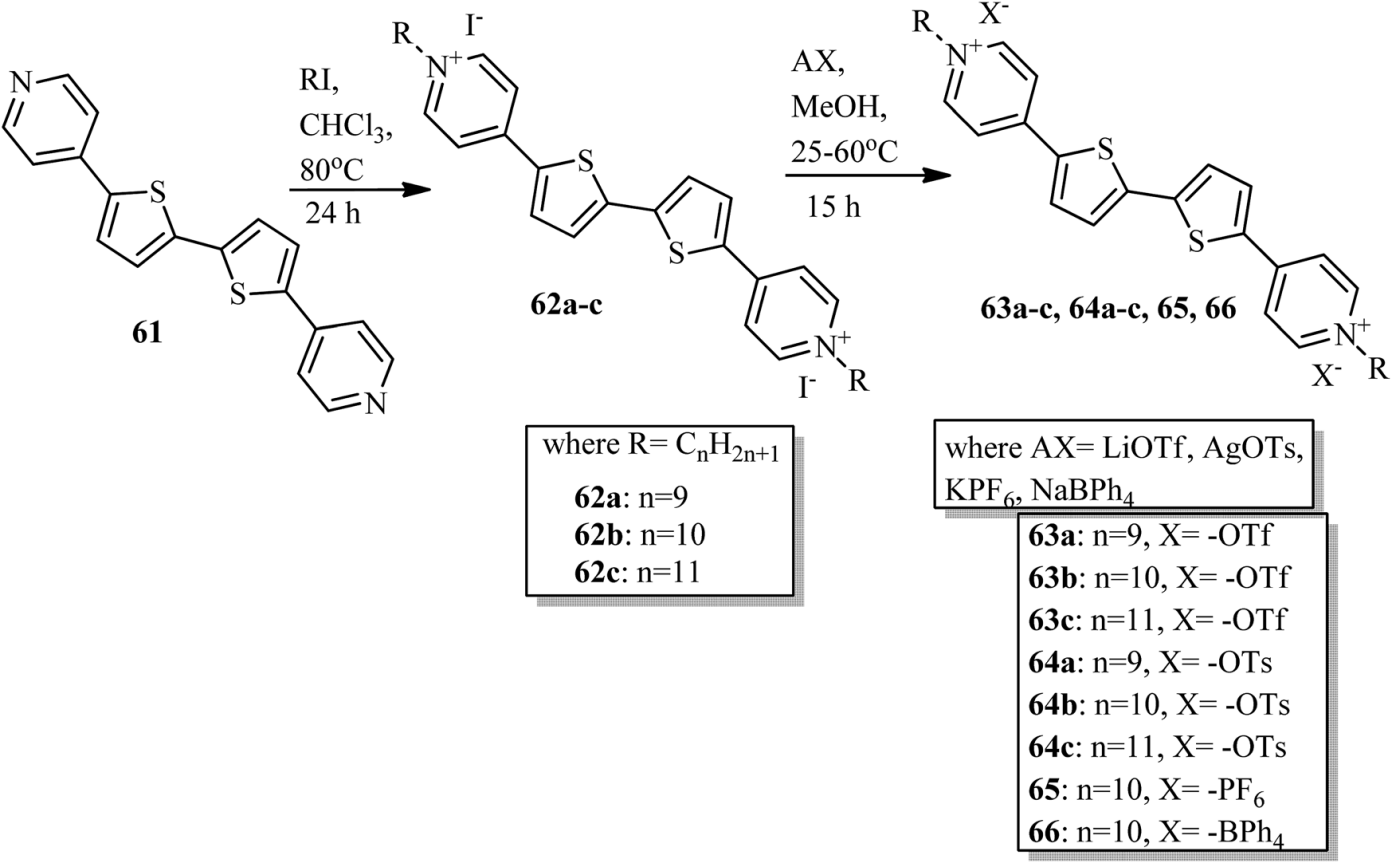
Synthesis of thienoviologen salts.

Veltri *et al.*^28^ prepared thienoviologen salts and studied the effects of the counter anion and the chain length on their liquid crystal properties. 4,4′-(2,2′-bithiophene-5,5′-diyl)bis(1-alkylpyridinium) was synthesized by referring to a previous report by Beneduci *et al.*,^29^ and the anions were changed by counter ion exchange reactions, as reported in Scheme 8. During POM observation, the authors did not note any mesophase formation in compound 65. The observed non-mesogenity may be due to the stronger interaction of hexafluorophosphate anion with the cation, leading to the formation of stable crystalline phase compounds. Similarly, for compound 66 (containing tetraphenylborate anion), liquid crystal phase was not observed because of the large anion, which further destabilized the formation of the stable dimeric structure. However, triflate 63a–c and tosylate 64a–c exhibited liquid crystalline properties above 150 °C; this was maintained up to 280 °C for the tosylate and 190 °C for the triflate, respectively. Smectic C phase was observed only in the complexes with shorter chain lengths (*i.e.*63a and 64a). In contrast, compound 63b showed nematic phase during both the heating and cooling cycles, but compound 63c exhibited nematic phase only during the cooling cycle. In addition, columnar phase was observed in the compounds of triflate 63a–c and tosylate 64a–c. Also, these compounds exhibited fluorescence properties in the bulk.

**Fig. 5:**
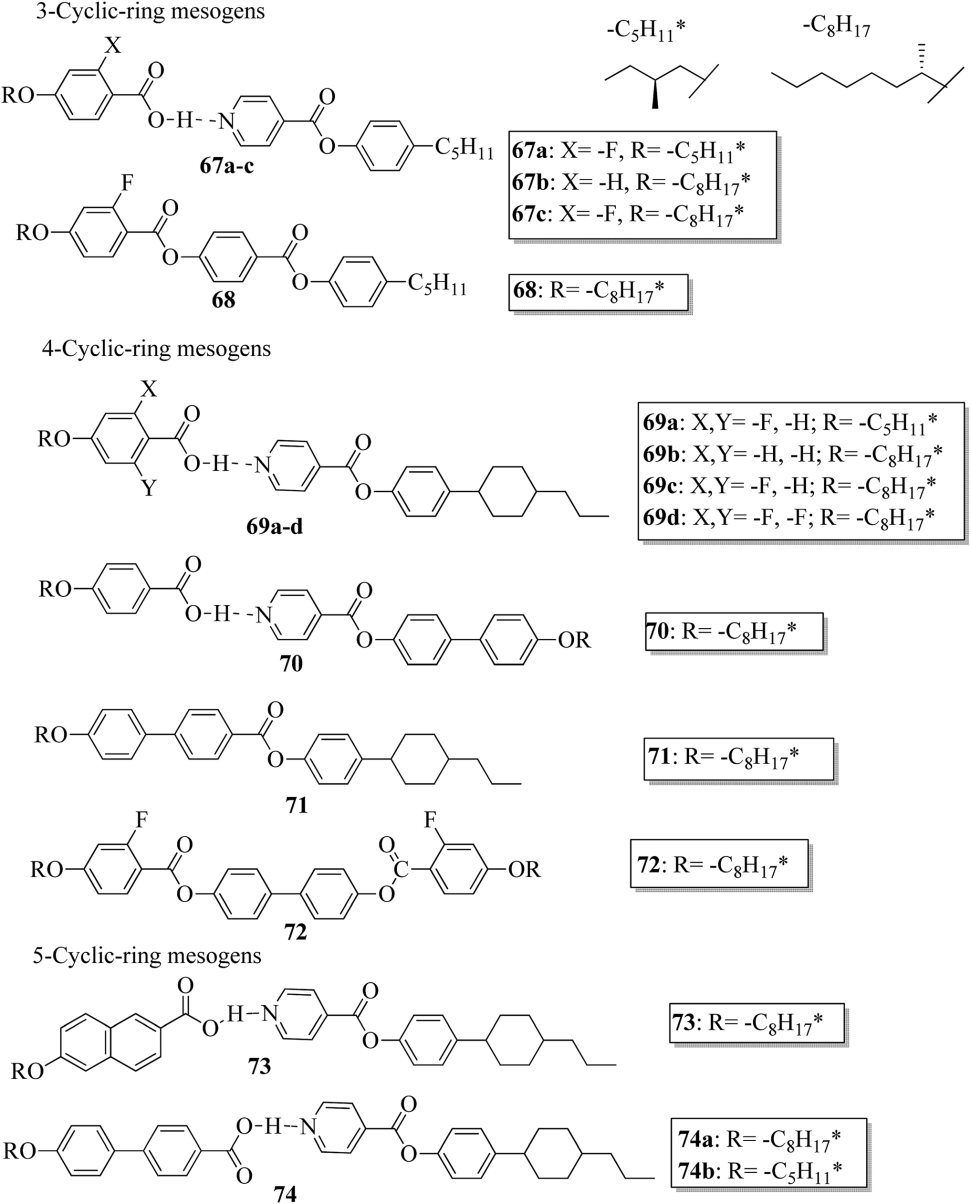
Molecular structures of hydrogen bonded complexes 67–74.

Further, He *et al.*^30^ synthesized hydrogen bonded mesogens containing chiral carbons and their C-bonded mesogens by referring to the previous report by He *et al.*;^31^ they also studied the effects of the length of the terminal chains and the mesogenic core on the mesogenic properties of the compounds and their performance for extending the temperature range of the blue phase. Fig. 5 presents the molecular structures of the prepared complexes 67–74. They found that the hydrogen bonded complexes exhibited lower phase transition temperatures than the analogous C-bonded compounds; also, the liquid crystal ranges were found to be narrower than those of the analogous C-bonded compounds. The complexes 67a and 67b exhibited monotropic nematic phases on cooling. However, their analogue 68 exhibited enantiotropic nematic phase. Complex 67c did not exhibit mesogenic properties. Compared to the 3-cyclic ring systems (67–68), the 4-cyclic ring systems (69–72) have high transition and clearing temperatures. Some of the hydrogen bonded complexes (69b–d) exhibited smectic A phases; however, this was not observed in their C-bonded analogous compounds. Among the 4-cyclic ring system-based compounds, compound 71 exhibited a high nematic phase range. In the 5-cyclic ring systems, compound 74a exhibited nematic as well as smectic phases, and the remaining two complexes (74b and 73) exhibited only nematic phases. The hydrogen bonded complexes had lower phase transition temperatures because the blue phase range moved to room temperature. The blue phase range was not extended in complex 67c due to the absence of mesogenic behavior in this complex. The widest blue phase range of about 12.9 °C was exhibited by the C-bonded 71-doped mixture compared to the hydrogen bonded complex. In the complex with an achiral acceptor, the ability of chiral transfer from the chiral hydrogen bonded mesogen to the host liquid crystal is weak, which results in a narrower blue phase range compared to the C-bonded analogues. Due to the weak ability of chiral transfer from the chiral hydrogen bonded mesogen to the host liquid crystal, the induced blue phase range of the C-bonded analog was wider than that of the hydrogen bonded complex with an achiral acceptor.

**Scheme 9:**
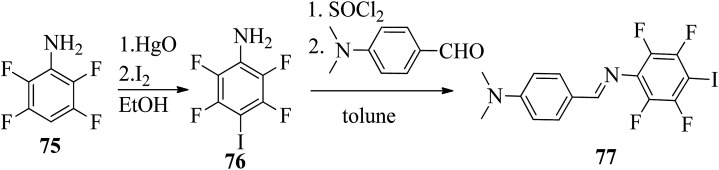
Synthesis of *N*-benzylideneaniline (77).

**Fig. 6:**
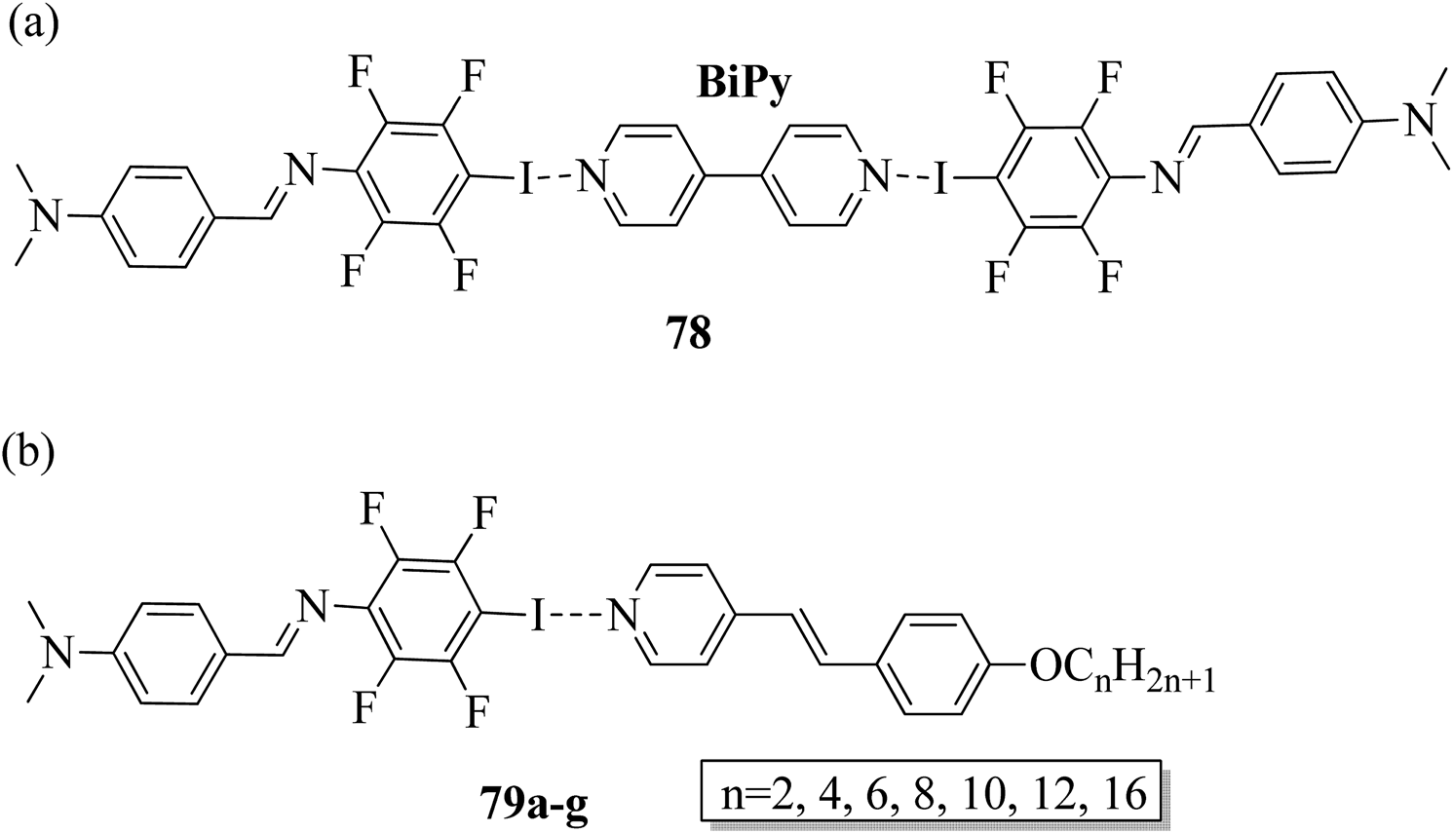
(a) Structure of halogen bonded complex 78; (b) structure of halogen bonded complexes 79a–g.

Wang *et al.*^32^ prepared halogen bonded complexes using *N*-benzylideneaniline 77 and 4-alkoxystilbazoles with pyridine as a proton acceptor moiety. They also prepared the halogen bonded co-crystal 78. By referring to the previous report by Bushuyev *et al.*,^33^ they synthesized compound 76 by iodination of tetrafluoroaniline (75) in the presence of iodine (I_2_) and mercuric oxide (HgO) in ethanol solvent. They condensed 4-dimethylaminobenzaldehyde and compound 76 in toluene solvent to synthesize *N*-benzylideneaniline 77 by referring to the previous report by Weiss and Pühlhofer^34^ (Scheme 9). Further, they synthesized the halogen bonded co-crystal 78 by dissolving a 2 : 1 ratio of compound 77 and bipyridine (BiPy) in THF solvent and allowing it to evaporate. In the crystal packing of compound 77, the two aromatic rings are not coplanar in alignment but are deviated towards each other. Additionally, there was no perceptible C–I⋯N interaction between 4-dimethylamino or imine and iodo-tetrafluorobenzene. However, it self-assembled into an unexpected fishbone-like assemblage by π⋯π, C–I⋯π and the coordination of various intermolecular interactions. In addition, they found that halogen bonded co-crystals (78) were formed, mainly due to C–I⋯NPy interactions. Furthermore, the authors prepared the hydrogen bonded complexes 79a–g by dissolving compound 77 and 4-alkoxystilbazoles in THF followed by slow evaporation of the solvent and drying under vacuum. Fig. 6 represents the structures of the halogen bonded complexes (78 and 79a–g). The DSC study revealed that liquid crystalline properties were not exhibited by the short chain complexes, *i.e.*79a and 79b. This was ascribed to the fact that short chain 4-alkoxystilbazoles are not flexible enough. When the chain length increased to *n* = 6 (*i.e.*79c), nematic phase with a droplet texture was observed during POM investigation. However, the DSC study did not display a distinct liquid crystalline phase transition for this compound. It was also observed that both crystalline and nematic phase co-existed, and the liquid phase gradually transformed to the crystalline phase. This is presumably due to the very close transition temperatures of isotropic to nematic phase and of nematic phase to crystallization. Monotropic long-lived nematic phases were observed clearly as the 4-alkoxystilbazole chain length increased (79d, 79e, and 79f), and typical Schlieren textures were observed by POM. Even after several expeditions into the isotropic phase, the thermal behaviors were found to be reproducible. Especially, longer lived nematic phase was observed for 79e and reproducibly occurred at 25 °C before crystallization. Decreases in the mesophase ranges were observed on further increasing the chain length for 79g, which exhibited a narrow range of nematic phase.

**Scheme 10:**
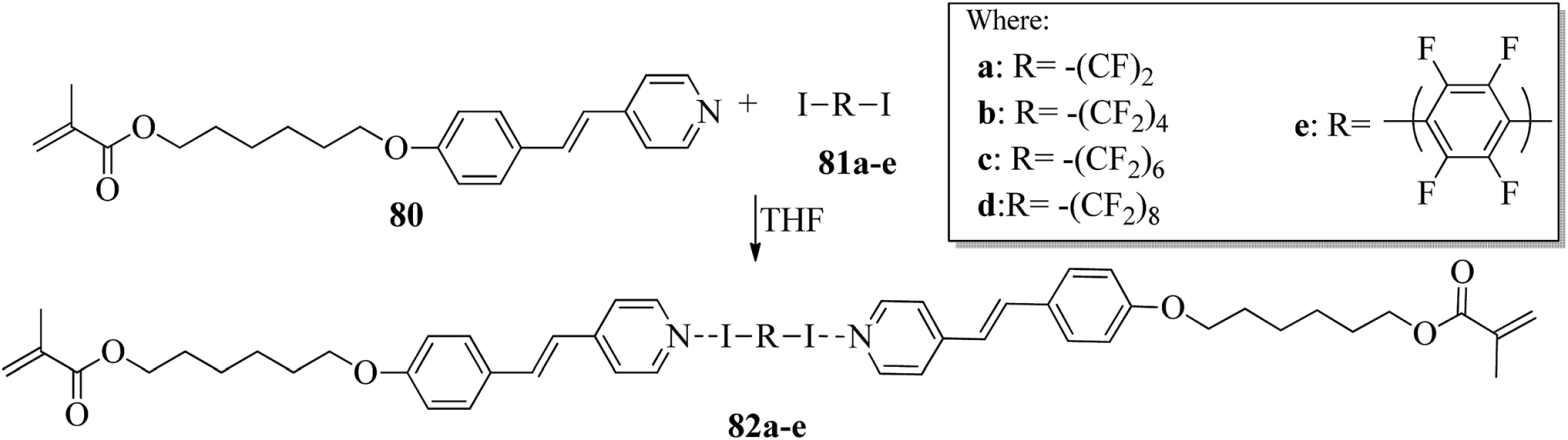
Preparation of halogen bonded complexes 82a–e.

Kumar *et al.*^35^ synthesized halogen bonded trimeric complexes using halogen bonded donors such as α,ω-diiodoperfluoroalkanes (81a–d) and 1,4-diiodotetrafluorobenzene (81e) and halogen bond acceptors such as methacrylate functionalized alkoxystilbazole (80). By referring to the literature report by Lin and Hendrianto,^36^ they synthesized the stilbazole methacrylate. Diiodoper-fluorocarbons (81a–e) and stilbazole methacrylate 80 were dissolved in THF solvent in 1 : 2 molar ratios, and crystallization from the solvent afforded the halogen bonded complexes (Scheme 10). The complexes obtained were yellow, whereas the parent compounds are white; this provides evidence for the successful formation of the complexes. This colour change was observed mainly because of the degree of charge shift from the pyridine nitrogen to the σ-hole on the iodine atom. Further, POM studies of the prepared 82a–e complexes revealed that all the parent compounds are non-mesogens, whereas the prepared 82b–e complexes are mesogens. The halogen bonded complexes 82a–e have higher melting points than the pure halogen bond acceptor and donors. This provides evidence for the preferable formation of well-defined chemical species 82a–e rather than mechanical blends of the parent molecules. The prepared complexes 82a–e melted directly into isotropic liquids on heating. Monotropic smectic A phase was observed in complexes 82b–e on cooling from the isotropic phase; however, complex 82a degraded promptly after melting. This result was observed because of the high volatility of the diiodoperfluoroethane molecule, which evaporated from the liquid mixture during heating. The complexes 82b–e showed perfectly reproducible mesogenic behavior even after various periods in the isotropic liquid phase, and smectic A phases were noted across a range of 20 °C to 30 °C ahead of the crystallization of the materials except for complex 82b, whose mesomorphic phase appeared to be long lived (up to 45 °C). Because of the segregation between hydrocarbon chains and the fluorocarbons, monotropic smectic A phase was observed. Further, single crystal X-ray diffraction studies revealed that N⋯I XB interactions are mainly responsible for the self-assembly of the corresponding molecules 80 and 81a–e and displayed noticeable separations between the hydro-carbon molecules and perfluorocarbons; this ideally encourages the formation of lamellar phase in the liquid crystal state.

**Fig. 7:**
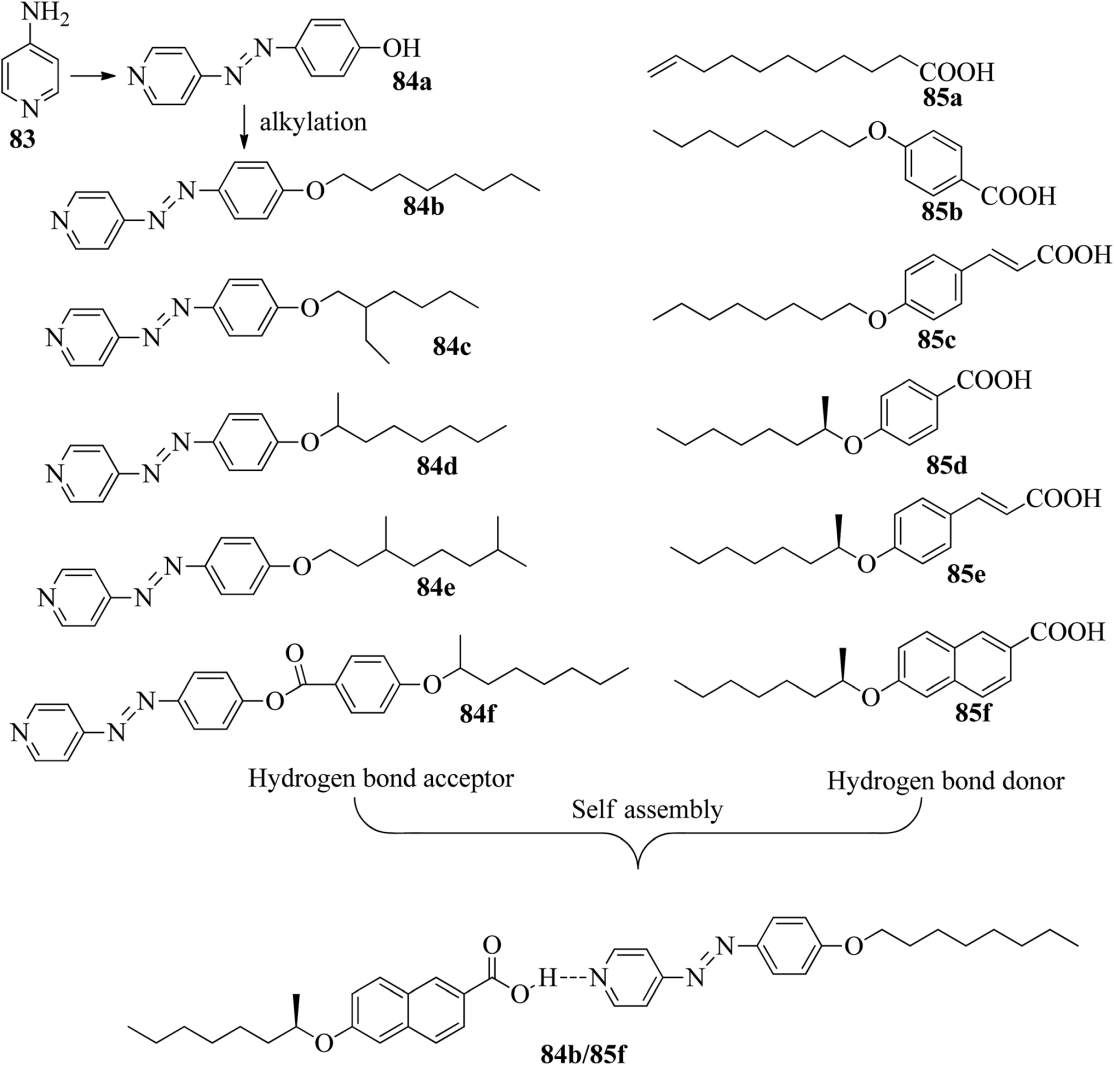
Structures of azobenzene precursors, proton donors and complexes (84b/85f).

He *et al.*^37^ synthesized pyridine-based azo compounds as hydrogen bond acceptors, *i.e.* 4-(4′-pyridylazo)-4′-substituted benzoates or 4-(4′-pyridylazo)-4′-alkoxybenzoates, with branched or straight terminals. Further, the authors prepared hydrogen bonded segregations using the synthesized hydrogen bond acceptors and different carboxylic acids. During synthesis, the 4-amino pyridine (83) and HCl were initially taken in a round bottom flask and cooled to 0 °C for some time, followed by the dropwise addition of sodium nitrate and phenol. Further, the reaction mixture was maintained in an ice bath and stirred for 0.5 h. A yellow precipitate of 4-(4-hydroxyphenylazo)pyridine (84a) was obtained by maintaining the pH of the reaction mixture at 6 to 7 with the addition of 10 wt% aqueous NaOH solution. Further, compound 84a, K_2_CO_3_, and KI were dissolved in acetone; to this, 1-bromooctane in acetone was added dropwise, and the mixture was refluxed for 16 h at 60 °C. After completion of the reaction, the reaction mixture was poured into water, and the product was extracted using ethyl acetate. The solvent was removed using a rotary evaporator, and the crude product was then purified through silica gel column chromatography to afford pure 4-(4-octyloxyphenylazo)pyridine (84b). In a similar manner, they synthesized compounds 84c–e using 1-bromo-2-ethyl hexane and (*R*)-2-octanyl tosylate as starting materials. The structures of the azobenzene precursors and proton donors are presented in Fig. 7. The compounds (*S*)-4-(octan-2-yloxy)benzoic acid, 4-(4-hydroxyphenylazo)pyridine, *N*-ethyl-*N*′-(3-dimethylaminopropyl)carbodiimide hydrochloride and 4-(*N*,*N*-dimethylamino)pyridine (DMAP) were dissolved in dichloromethane solvent and stirred at room temperature for 20 h. After completion of the reaction, the reaction mixture was filtered and the solvents were removed under reduced pressure. Finally, the product 4-(4-hydroxyphenylazo)pyridine4-(octan-2-yloxy)benzoate (84f) was purified through chromatography. Further, the hydrogen bonded complexes were prepared by dissolving appropriate amounts of hydrogen bond acceptors and donors in THF solvent followed by solvent removal using reduced pressure and drying in vacuum for 24 h at room temperature.

Liquid crystalline studies on these materials revealed that all the starting materials except 85b and 85c are non-mesogens; compound 85b exhibited smectic C phase and compound 85c exhibited nematic phase. The liquid crystalline properties of the prepared mixtures were studied using POM and DSC. Furthermore, the studies revealed that in the case of the precursors, the melting and clearing points increased with the introduction of terminal alkyl chains and decreased with increasing length of the rigid structures. Most of the complexes prepared using 84b exhibited only smectic A phase. However, the 84b/85b and 84b/85f complexes exhibited nematic phase in addition to smectic A phase. In the 84c-based complexes, only 84c/85c exhibited nematic phase; all the other complexes were non-mesogens. In the 84d-based complexes, nematic phase was observed in 84d/85d, 84d/85c, and 84d/85f complexes, and the remaining complexes are non-mesogens. In the 84e-based complexes, only 84e/85c exhibited nematic phase, and all the other complexes are non-mesogens. In the 84f-based complexes, all the complexes exhibited nematic phase and twist ground boundary A phase except the 84f/85a complexes, which exhibited only smectic A phase. Finally, they concluded that mesomorphic states were observed in the straight terminal hydrogen bonded complexes, while the azo-derived complexes exhibited mesogenic behavior only with branched terminals. In general, when the proton donor is altered from a flexible chain to one or two rigid rings in its structure, the length-to-breadth ratio increases, which causes the extended mesogenic properties and increases in the phase transition points.

**Fig. 8:**
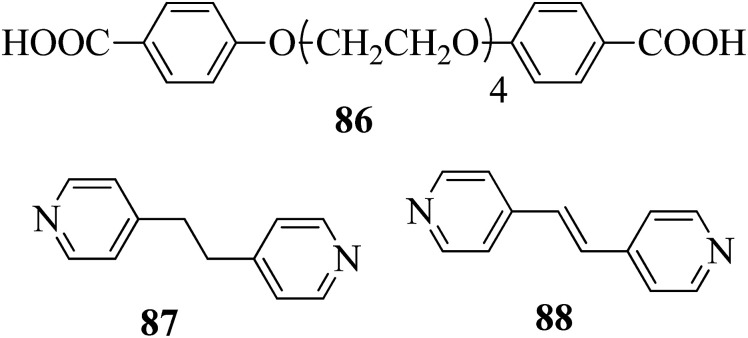
Structures of proton donor 86 and proton acceptors 87 and 88.

Carli *et al.*^38^ prepared a series of hydrogen bonded liquid crystalline supramolecular copolymers. They synthesized the tetra(ethyleneglycxoy)bis-4-benzoic acid (86) by referring to the literature report by Greuel *et al.*;^39^ later, it was mixed with the proton acceptors 1,2-bis(4-pyridyl)ethene (88) and 1,2-bis(4-pyridyl)ethane (87) and converted to a molten state for two minutes in nitrogen atmosphere, followed by cooling the mixtures to room temperature to yield the expected complexes. Fig. 8 presents the structures of proton donor 86 and the proton acceptors 87–88. Mesophase was observed when the rigid bis pyridyl (88) was hydrogen bonded; however, in the case of the structurally analogous flexible bispyridyl (87), mesophase was not observed. The complexes containing 87 and 88 as proton acceptors showed nematic phase at 75% addition of 88, and smectic C phase was observed below 50% addition of 88. As the 87 content increased, the lifetime of the liquid crystalline property decreases due to the enormous eutectic effect. Only isotropic melting and crystallization were observed in complexes containing only 86 and 87 (0% 88). Hence, they concluded that a minimum amount of rigid 88 module was essential to establish the liquid crystalline phase. 25% of the accessible compounds containing pyridine moiety are essential to establish the mesophase demand arising from 87. Several heating and cooling cycles did not exhibit divergent mesogenic features, suggesting that the mesophases produced are the thermodynamically ideal structures. In addition, the increased stability of the mesophases at high constitution is notable due to the organizational similarities between the non-mesogenic and mesogenic acceptors. In the excess mixtures, the mesophase obtained was powerful and capable of overcoming notable contamination from the competitive species. Further, increased mesophase lifetime was contributed by the “molecular peer pressure” phenomenon, *i.e.* an *anti* conformer of the alkane chain in 87 imposed by molecular restrictions.

**Scheme 11:**
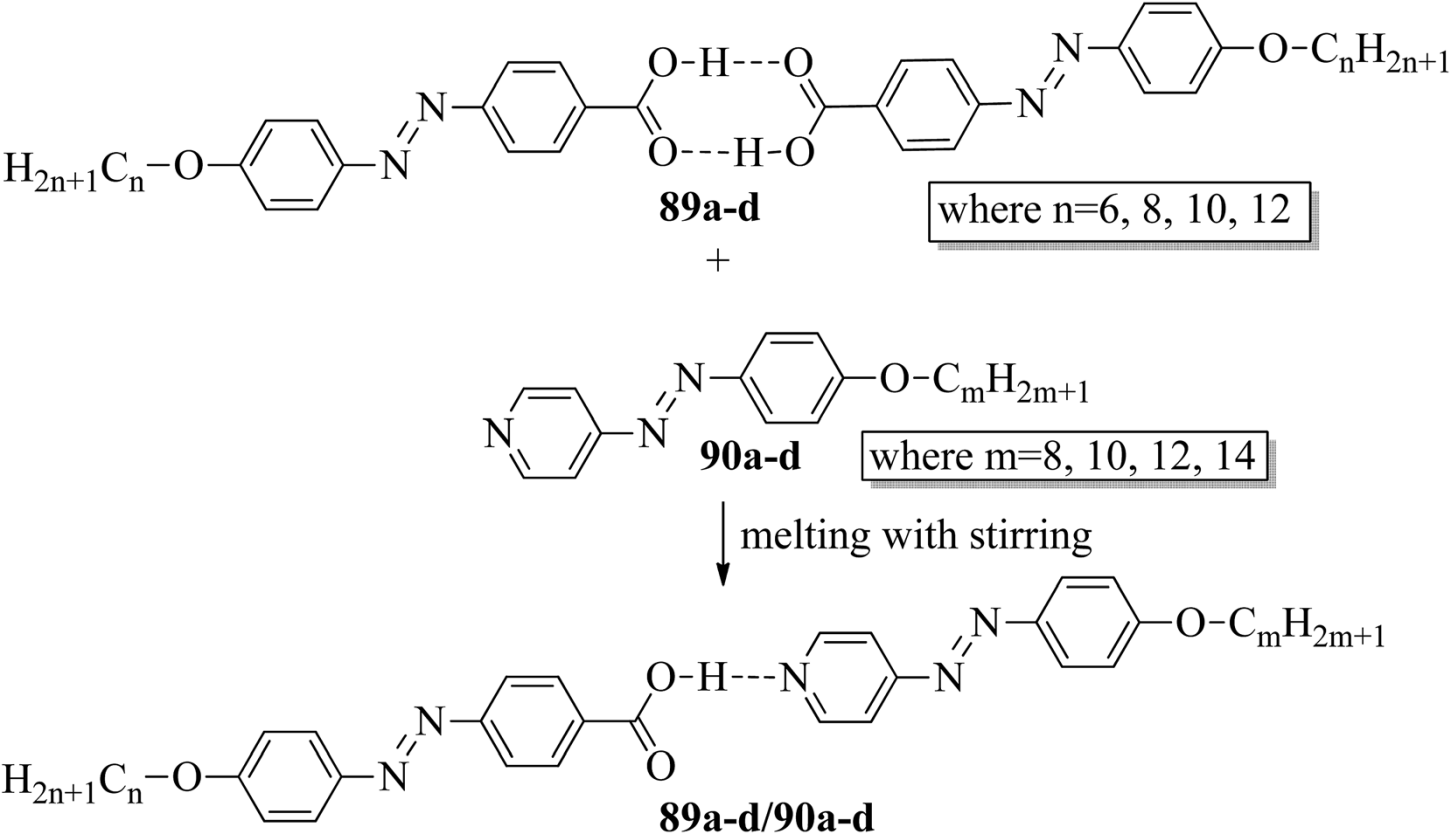
Preparation of hydrogen bonded complexes 89a–d/90a–d.

Ahmed *et al.*^40^ prepared supramolecular hydrogen bonded complexes using 4-alkoxyphenylazo-benzoic acids (89a–d) and 4-alkoxyphenylazo pyridines (90a–d). Using polarizing light microscope (PLM) and DSC analysis, they studied the mesophase behavior. Hydrogen bond formation between the prepared complexes was confirmed by DSC and FT-IR spectroscopy techniques. By referring to previous work (Alaasar *et al.*,^41^ Janietz and Bauer,^42^ Ahmed *et al.*^43^), they prepared the proton acceptor and proton donor molecules, *i.e.* 4-alkoxypheylazo-4′-pyridines (90a–d) and 4-alkoxyphenylazo-benzoic acids (89a–d), respectively. The pure benzoic acid derivatives displayed nematic phase (N) on a very minute scale and smectic C phase with relatively high transition temperatures (Janietz and Bauer,^42^ Ahmed *et al.*^43^); meanwhile, azopyridines 90a–d are non mesogens, *i.e.* they directly melt to the isotropic liquid phase from the crystalline phase (Alaasar *et al.*^41^). Supramolecular aggregations (89a–d/90a–d) were developed by melting two reciprocal modules in 1 : 1 molar ratios with stirring to obtain an intimate blend, followed by cooling to room temperature (Scheme 11). All the prepared complexes displayed two solid crystalline phases, followed by a smectic C phase and an observed broad nematic phase at the end; this was induced in these complexes, *i.e.* it was not observed in the cases of the pure acid components 89a–d. In all the samples, a more narrow range of smectic C phase than of nematic phase was observed. Additionally, an increase in *m* moderately influenced the nematic phase range, whereas an increase in *n* increased the nematic stability (*T*_N-I_). Also, the results revealed that for a specific value of *m*, the length of the alkoxy-chain in the acid derivatives (*n*) moderately influences the melting temperatures of the supramolecular aggregations. Consequently, despite the fact that azopyridine compounds 90a–d do not display any mesophases, all the developed aggregations 89a–d/90a–d displayed induced nematic phases with relatively vast temperature spans, with a higher value of about 65.4 °C for 89c/90a aggregation and a lower value of about 37 °C for 89b/90b aggregation. Also, an increase in the alkoxy chain length of the acid complement (*n*) causes an increase in the nematic transition enhancement (Δ*T*); therefore, the alkoxy chain length of the acid complement (*n*) was found to have a greater effect on the nematic phase stability. Further, widening of the nematic phase was promoted by the increase in molecular anisotropy in the prepared supramolecular aggregations, in accord with previous reports (Ahmed *et al.*^43^). Furthermore, these studies revealed that the stabilities of smectic C and nematic phase increase as the mesogenic core length increases.

**Scheme 12:**
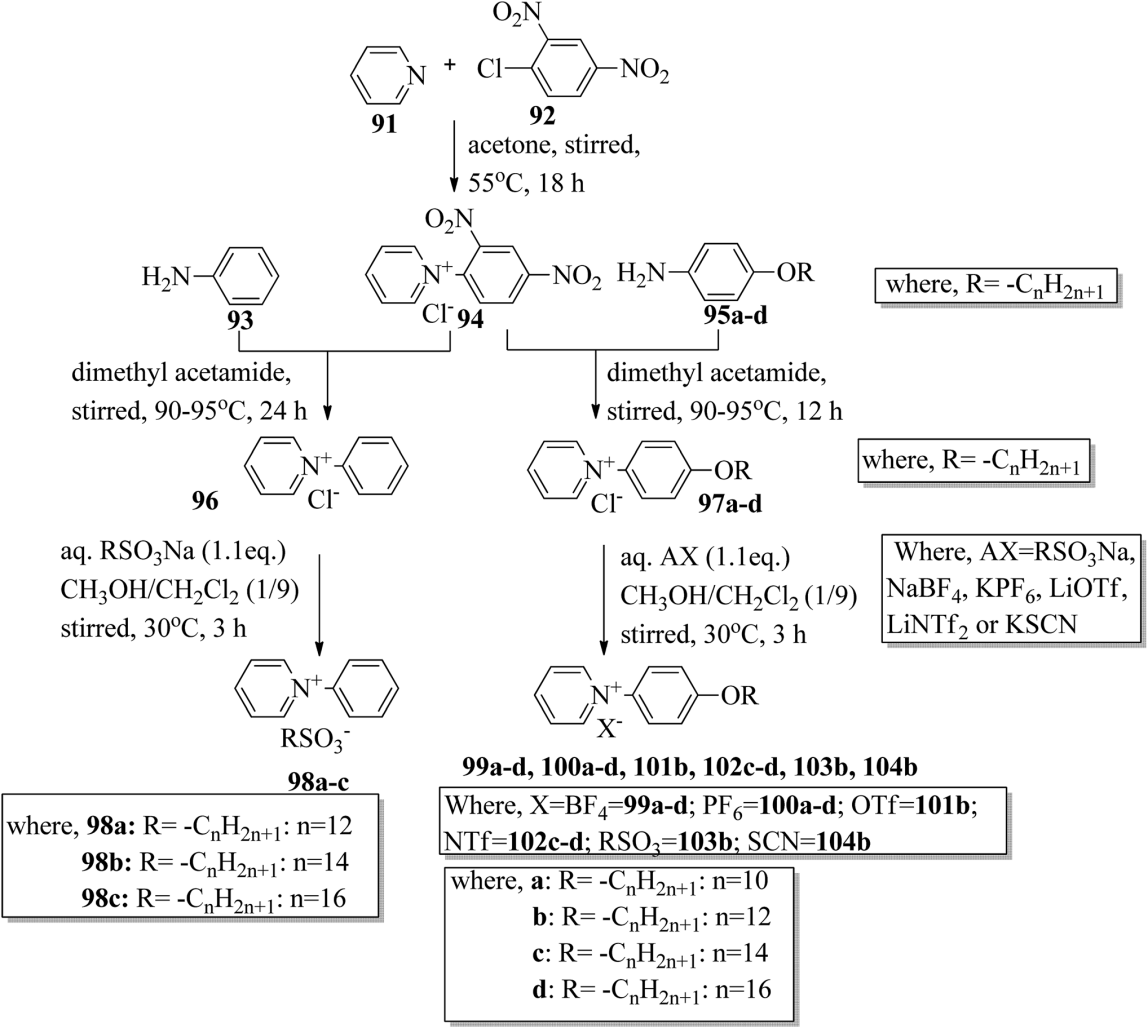
Synthetic route for ionic liquid crystals 98–104.

Wang *et al.*^44^ synthesized two series of ionic liquid crystals, *i.e. N*-phenylpyridinium derivatives (98–104). By studying previous literature (Michels *et al.*,^45^ Zeghbib *et al.*,^46a^ Kuwabara *et al.*,^47^ Kuo *et al.*^48^), they synthesized precursors such as *N*-phenyl pyridinium chloride 96 and *N*-(4-alkoxyphenyl)pyridinium chloride 97a–d. To substitute the chloride into different anions, they used methanol as a phase transfer medium. To prepare this, they dissolved the metal salt solution (RSO_3_Na, NaBF_4_, KPF_6_, LiOTf, LiNTf_2_ or KSCN) in water, and this was added to a solution of 96 or 97a–d precursor in CH_3_OH/CH_2_Cl_2_ (1/9). After 30 min, the milky or cloudy solution became clear, followed by continuous stirring for 3 h at 30 °C. Scheme 12 presents the synthetic route for the ionic liquid crystals 98–104. X-ray crystal structure analyses revealed that hydrogen bonds in the solid state induced bilayer lamellar or sandwiched head-to-head structures. In addition, the anion was embedded near the head group. All the compounds exhibited enantiotropic behavior except compounds 100a and 102c; these compounds exhibited monotropic behavior. DSC data of the compounds (98a–c) revealed that the melting temperature remained at 91.0–107.0 °C while the clearing temperature (*T*_cl_) increased with carbon chain length; *i.e. T*_cl_ = 133.0 °C (98a) < 157.0 °C (98b) < 202.6 °C (98c). As the chain length increases, the smectic A (SmA) phase range also increases; Δ*T*_SmA_ = 54.0 °C (98a) < 77.0 °C (98b) < 138.1 °C (98c) during the cooling cycle. Further, the expanded temperature range of Δ*T*_SmA_ = 138.1 °C in the derivative 98c indicates that this derivative has an almost ideal characteristic ratio of *d*/*l*, which is essential for mesophase formation. Also, the clearing temperature of compound 103b was higher than that of compound 98a; *i.e. T*_cl_ = 133.0 °C < 199.0 °C. Here, the dodecyloxy chain-substituted compound 103b facilitated the mesophase formation. On the other hand, the mesophase range of 98a was narrower than that of 103b, *i.e.* Δ*T*_meso_ = 85.4 °C > 54.0 °C during the cooling cycle. Further, the DSC data of compounds 99a–d indicated that the melting temperature remained at *T*_melt_ = 83.7 °C to 88.6 °C; at the same time, the clearing temperature increased with carbon chain length, *i.e. T*_cl_ = 170.1 °C (99a) < 216.8 °C (99b) < 252.9 °C (99c) < 272.9 °C (99d). As the chain length increases, the smectic A phase range also increases; Δ*T*_SmA_ = 116.0 °C (99a) < 173.9 °C (99b) < 210.1 °C (99c) < 234.3 °C (99d) during the cooling cycle. Among all the counter anions, the smallest temperature ranges of smectic A phase were observed in compounds 102c–d; Δ*T*_SmA_ = 11.0 °C (99c) < 26.5 °C (99d) during the cooling cycle. Also, they exhibited lower clearing temperatures than the other compounds; *T*_cl_ = 56.8 °C (99c) < 77.8 °C (99d). Due to the weaker coulombic forces in the unsymmetric and bulky NTf_2_ compounds, they exhibited lower clearing temperatures. The mesophase stability was significantly influenced by the type of counter anion; Δ*T*_SmA_ = 221.3 °C (97b) > 213.1 °C (104b) > 173.9 °C (99b) > 85.4 °C (103b) > 63.8 °C (100b) > 41.4 °C (101b). A significantly wider smectic A phase range was observed in compound 99d (*i.e.* Δ*T*_SmA_ = 234.3 °C) which is broader than those of other reported ionic liquid crystals. Also, compound 102c is the first phenylpyridinium-based ionic liquid crystal. Among the prepared compounds, compound 104b showed room temperature mesophase.

**Scheme 13:**
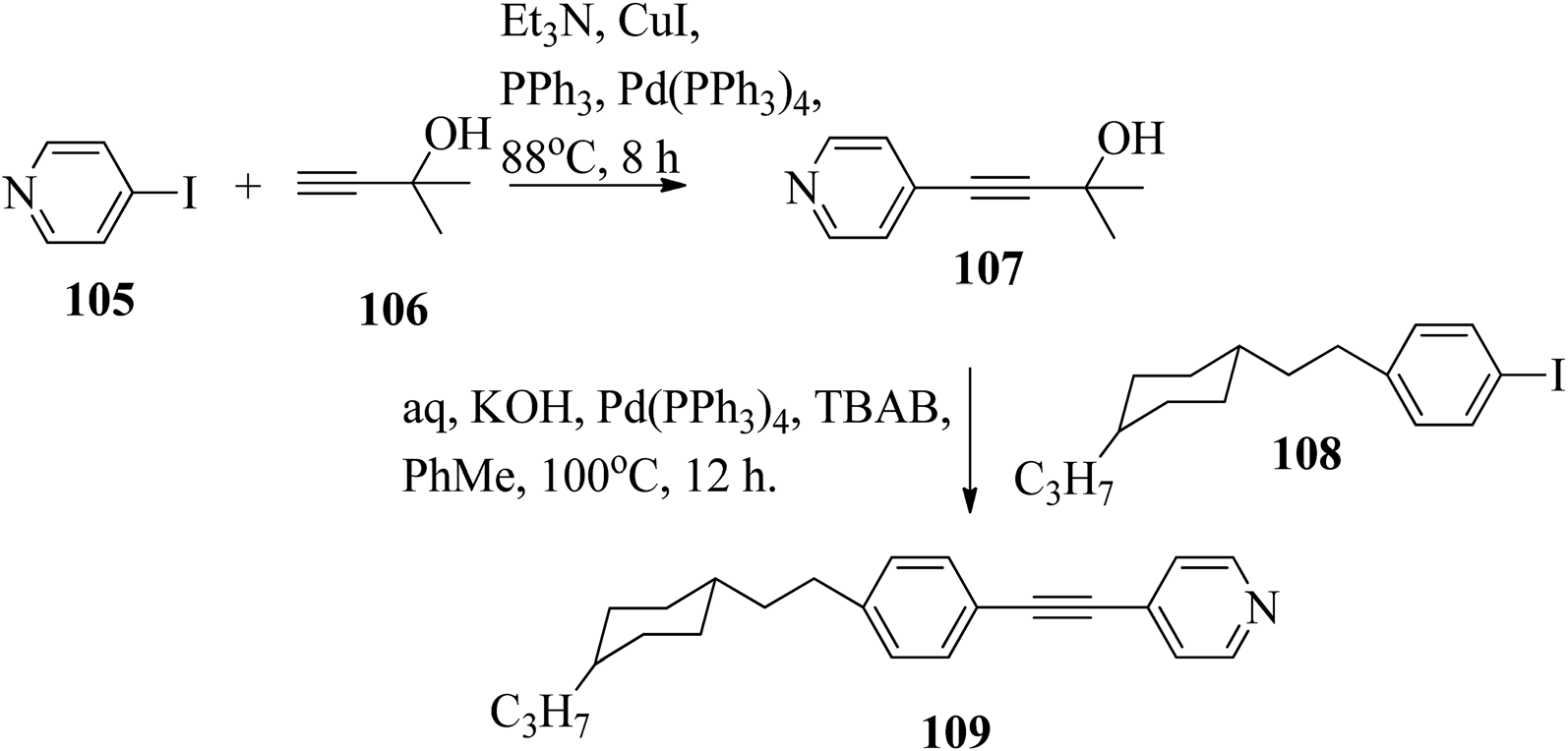
Synthetic route for 109.

Chen *et al.*^49^ synthesized compounds 109 and 116, which are dual functional molecules, and used them to observe rod-like liquid crystals on dye-sensitized solar cells. 4-(2-(*trans*-4-*n*-Propylcyclohexyl)ethyl)iodobenzene (108) was synthesized as described in a previous report by Chen *et al.*,^50,51^ and organic dye 117 was synthesized by referring to a previous report by Wu *et al.*^52^ 2-Methylbut-3-yn-2-ol (106) in triethylamine (Et_3_N) was added to DMF solvent containing 4-iodopyridine (105), CuI, Pd(PPh_3_)_4_, and PPh_3_ in dry Et_3_N at room temperature. After completion of the reaction, the mixture was cooled and filtered; then, DCM was added to it, and the organic layer was washed using saturated ammonium chloride solution and dried using anhydrous MgSO_4_. Later, the solvents were evaporated to yield 2-methyl-4-(pyridin-4-yl)but-3-yn-2-ol (107). A mixture of KOH, acetylenic alcohol 107, and TBAB in PhMe/H_2_O at a ratio of 4 : 1 (v/v) was stirred for 30 min at 60 °C under nitrogen protection. Further, 108 and Pd(PPh_3_)_4_ were added, and the reaction mixture was stirred for 12 h at 100 °C. After completion of the reaction, the solution was filtered over a pad of silica gel. The mixture was diluted using water and extracted using ethyl acetate. The combined organic phase was dried using MgSO_4_. After removal of solvent in vacuum, the product 4-((4-(2-(*trans*-4-*n*-propylcyclohexyl)ethyl)phenyl)ethynyl)pyridine (109) was purified using silica gel column chromatography (Scheme 13). Further, 1-bromo-4-iodobenzene (110) and 4-pyridineboronic acid (111) were dissolved in DMF separately and mixed, then added to the K_2_CO_3_ solution. This reaction mixture was degassed for 10 min under nitrogen atmosphere; then, Pd(PPh_3_)_4_ was added and the mixture was stirred for 15 h at 60 °C. After completion of the reaction, the solvent was removed by rotary evaporation and the compound was dissolved using CH_2_Cl_2_ solvent and washed with water and brine solution. Then, the organic layer was dried using magnesium sulphate, concentrated and purified using chromatography to afford pure 4-(4-bromophenyl)pyridine (112). Ethynyltrimethylsilane (113) in Et_3_N was added to DMF solvent containing 4-(4-bromophenyl)pyridine (112), CuI, Pd(PPh_3_)_4_, and PPh_3_ in dry Et_3_N at room temperature. The reaction mixture was stirred at 60 °C for 8 h under nitrogen atmosphere. After completion of the reaction, the mixture was cooled and filtered; then, DCM was added, and the organic layer was washed using saturated ammonium chloride solution and dried using anhydrous MgSO_4_. The solvents were evaporated to yield 4-(4-((trimethylsilyl)ethynyl)phenyl)pyridine (114). Further, compound 114 and potassium carbonate were dissolved in methanol and stirred for 4 h. After the completion of the reaction, DCM extraction was performed, followed by washing with water and brine. Further, the organic layer was dried, followed by evaporation of the solvent under reduced pressure to yield 4-(4-ethynylphenyl)pyridine (115).

**Scheme 14:**
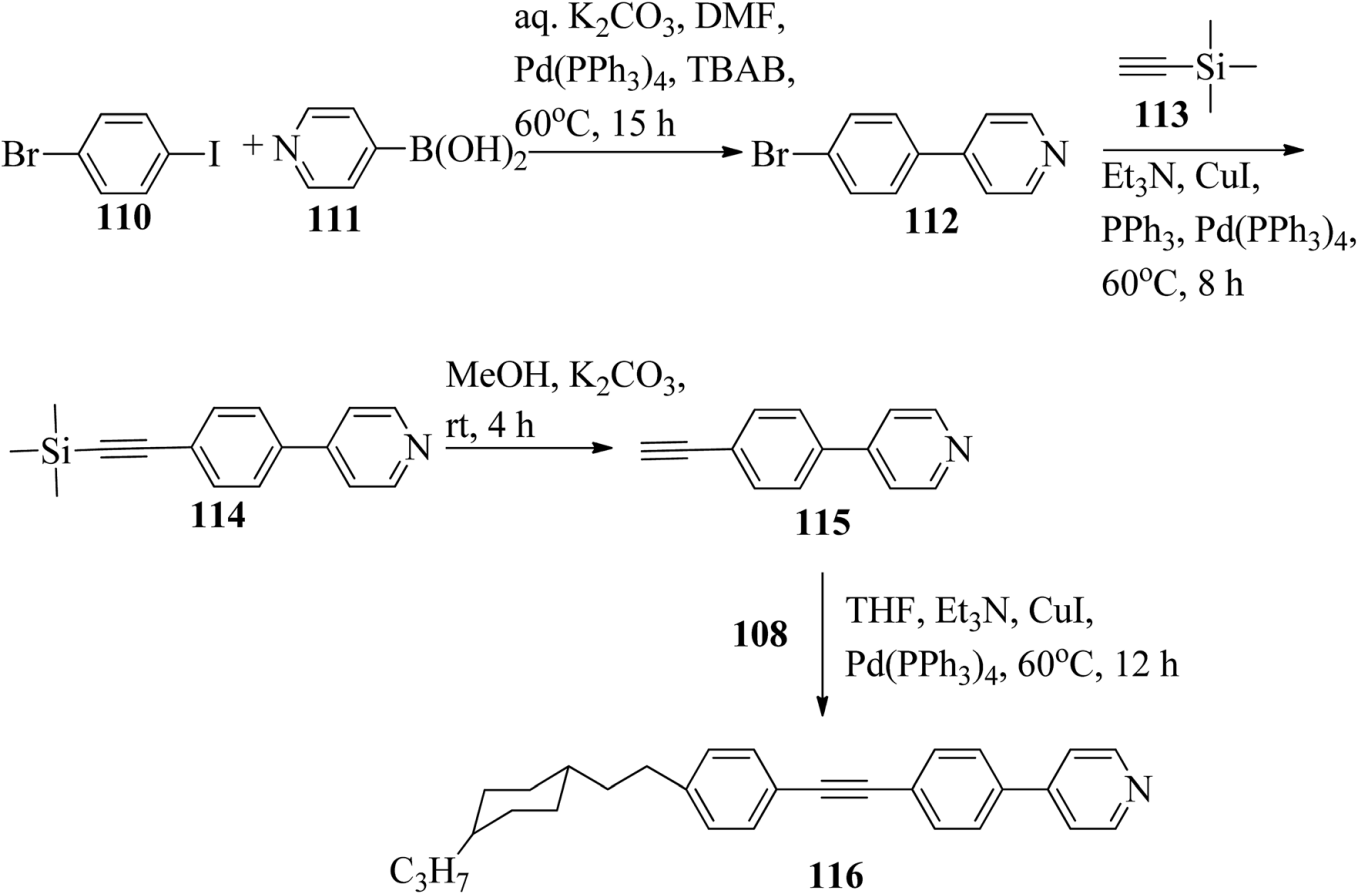
Synthetic route for 116.

**Fig. 9:**
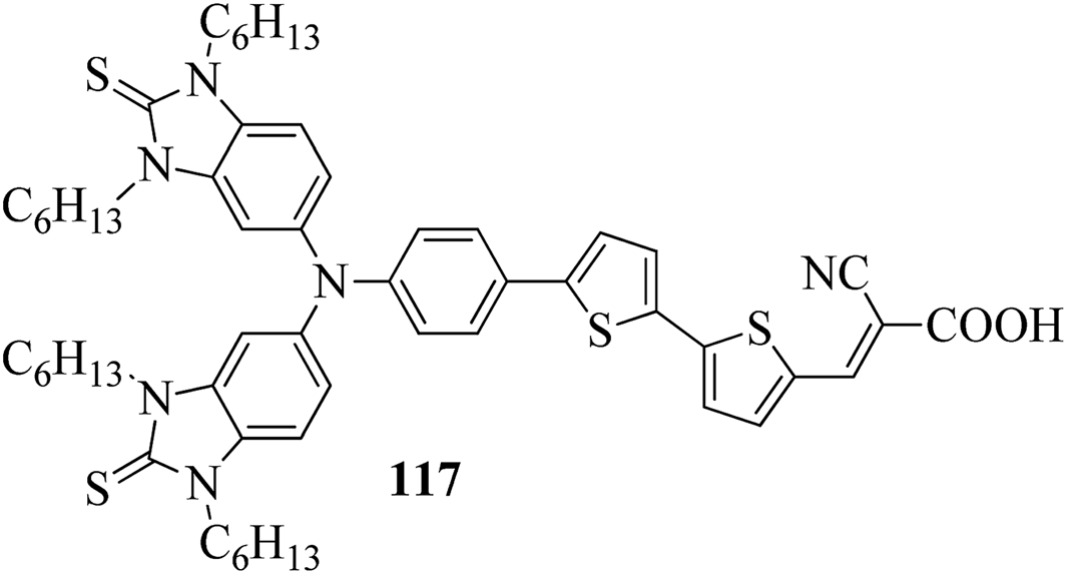
Structure of 117.

In a two-way flask, compound 115 and Et_3_N were dissolved in THF, and the solution was bubbled with nitrogen. 4-(2-(*trans*-4-*n*-Propylcyclohexyl)ethyl)iodobenzene (108), Pd(PPh_3_)_4_, and CuI were taken in another two-way flask containing THF solvent and covered with nitrogen; to this, the previously prepared mixture was added dropwise. This reaction mixture was stirred for 12 h at 60 °C. Saturated ammonium chloride was added to remove CuI. Using water, the reaction mixture was diluted and extracted with ethyl acetate. Using MgSO_4_, the combined organic layer was dried followed by evaporation of solvent in vacuum to yield 4-(4-((4-(2-(*trans*-4-*n*-propylcyclohexyl)ethyl)phenyl)ethynyl)phenyl)pyridine (116), as shown in Scheme 14. Later, the crude product was purified by silica gel column chromatography. Using POM, DSC, and XRD experiments, the mesogenic behavior of 109 and 116 was studied. POM observation revealed that 109 and 116 exhibit nematic mesophase in the cooling scan. Liquid-like ordering with a diffuse peak in the wide-angle region with an intermolecular distance of 4.6 Å was observed when 109 was cooled from isotropic phase. Compound 109 exhibited monotropic phase (*i.e.* it exhibited nematic phase only in the cooling cycle). Compound 116 exhibited enantiotropic nematic phase (*i.e.* it exhibited nematic phase in both the heating and cooling cycles). On the other hand, these two mesogens exhibited different effects on the photo-conversion efficiency and long term stability of co-sensitized dye-sensitized solar cells 109/117 and 116/117. Fig. 9 presents the structure of 117. The molecular structures of 109 and 116 show steric repulsion of molecules and inter-annular twisting, which is due to the additional phenyl ring on 116. Hence, the π–π packing is minimized and nematic mesophase is induced despite the lengthening of the molecular axis.

**Scheme 15:**
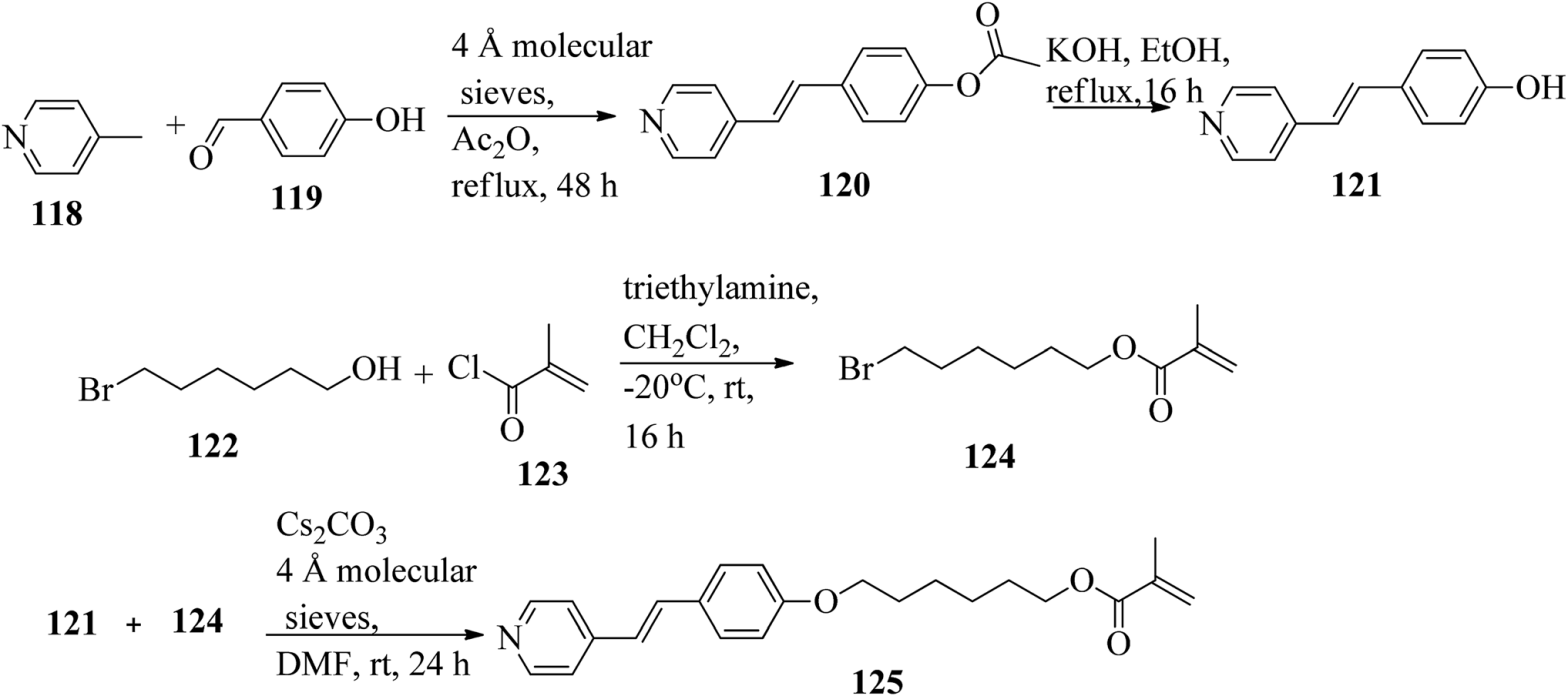
Synthesis of 125.

**Fig. 10:**
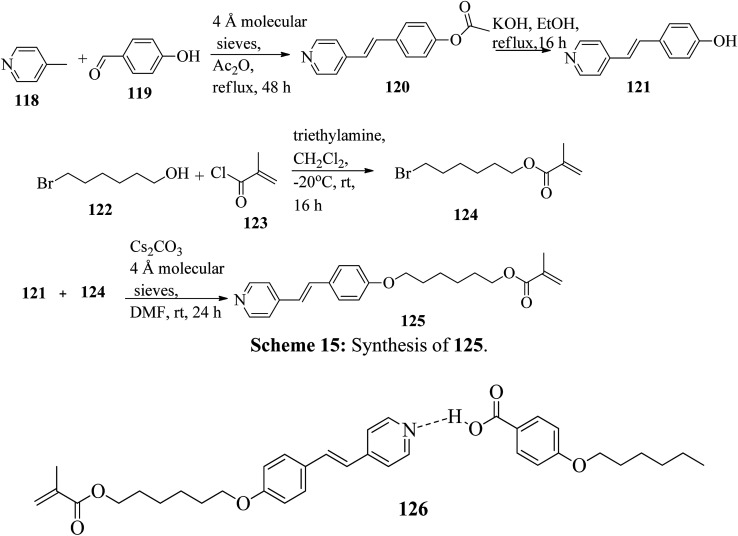
Structure of hydrogen bonded complex 126.

Mulder *et al.*^53^ prepared hydrogen bonded hetero-dimers for the fabrication of a cationic nanoporous smectic liquid crystal network (LCN). The preparation procedures depend on supramolecular aggregations composed of non-reactive benzoic acid rigid materials hydrogen bonded with a pyridyl-bearing reactive mesogen. By referring to procedures given in reports by Shaw *et al.*^54^ and Chiang *et al.*,^55^ they synthesized the compound 4-acetoxystilbazole (120). Afterwards, 4-hydroxystilbazole (121) was synthesized by hydrolyzing the acetic acid ester 120 in ethanolic KOH. In parallel, 6-bromohexyloxymethacrylate (124) was synthesized as described in the previous report by Stumpel *et al.*^56^ Finally, an Williamson ether synthesis reaction of compounds 121 and 124 utilizing caesium carbonate (Cs_2_CO_3_) in anhydrous DMF at room temperature led to the formation of 4′-(6-methacyloxyhexyloxy-4-stilbazole) (125) (Scheme 15). Fig. 10 presents the structure of hydrogen bonded complex 126. The liquid crystal network was prepared by mixing different amounts of cross linker (CL) (*trans*-4-(4-(11-acryloyloxyundecyloxy)cyclohexanecarboxyloxy)phenyl-4-(11-acryloyloxyundecyloxy) with 126. To carry out the photopolymerization, 0.5 wt% thermal inhibitor (butylated hydroxytoluene, BHT) and 1 wt% photo initiator (Irgacure 819) were added. To obtain a homogeneous mixture, all the compounds were dissolved in dichloromethane solvent. After mixing, the solvent was removed in vacuum. 20 μm thick sheets were developed by capillary suction of the liquid crystal-monomer blends in the melt (125 °C) between two exactly separated glass slides (LC cell). After filling the cell, the blend was cooled to 30 °C. Afterwards, the polymerization reaction was performed by UV illumination (provided with a 405 nm cut-off filter) for about 600 s. To acquire homeotropic and planar alignments, the glass slides (LC cell) were provided with rubbed octadecyltrimethoxysilane and polyimide, respectively. Using POM and DSC, they studied the liquid crystal properties of 126. Smectic A phase was observed at 140 °C in 1 : 1 aggregates. Smectic C phase was observed on further cooling from 119 °C to 110 °C. High levels of tilted smectic phases were noted from 80 °C to −4 °C; further lowering the temperature resulted in crystallization. Also, they studied mixtures of 126 with different amounts of cross-linker (CL). The clearing temperature decreases as the amount of CL increases, and the tilted smectic phase vanishes. For example, when 50 wt% CL was used, a narrow nematic phase from 119 °C to 117 °C, a smectic A phase from 117 °C to 59 °C, and a smectic B phase from 59 °C to −15 °C were observed under POM during the cooling cycle from the isotropic phase. Upon addition of a cross-linker, secured smectic phase can be fixed by photopolymerization. However, the lamellar structure was found to be maintained after removal of the template when 25 wt% or more cross-linker bearing a nano-porous LCN was used. Also, anisotropic and high anhydrous proton conductivity were obtained by a cationic 2D nanoporous polymer, which was obtained after immobilization of phosphoric acid (H_3_PO_4_) in the LCN pores. Further, these results revealed that 2D cationic nano-porous polymers can be fabricated with attractive practical properties. It is anticipated that cationic nano-porous polymers which are pH independent can be fabricated by alkylation of the pyridine component. These polymers are attractive to adsorb and sense or separate negatively charged molecules and anions.

**Fig. 11:**
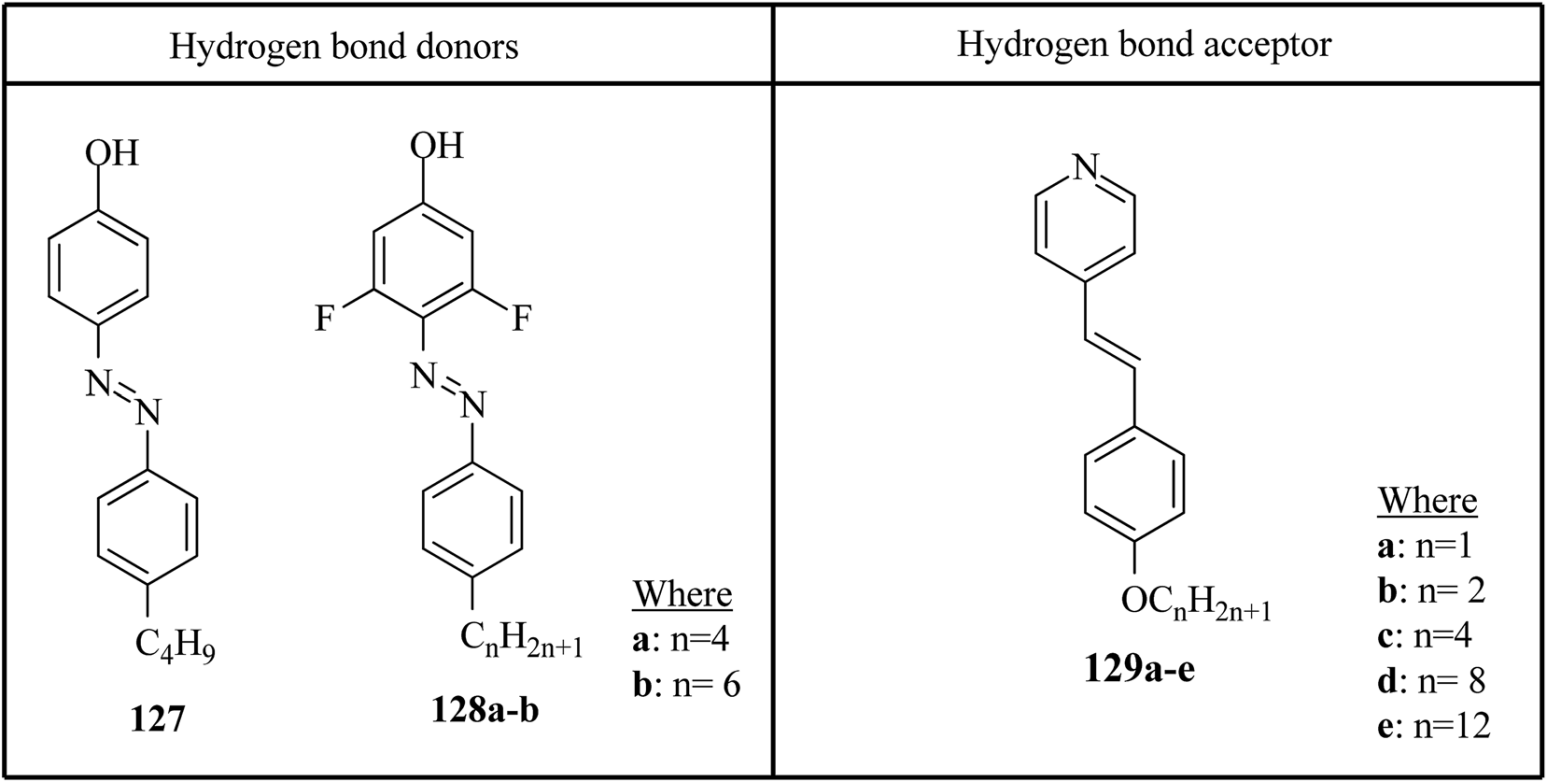
Structures of hydrogen bond donors 127 and 128a–b and acceptors 129a–e.

Saccone *et al.*^57^ prepared fifteen supramolecular complexes using stilbazoles and azophenols as proton acceptors and proton donors, respectively, and studied the effects of *ortho*-fluorination of the azophenol on the mesogenic behavior. By diazotization and azo-coupling reactions, they synthesized di-fluorinated (128a–b) azophenols. By referring to the report by Bruce *et al.*,^58^ they synthesized the proton acceptors 129a–e. Fig. 11 presents the structures of the hydrogen bond donors (127 and 128a–b) and acceptors 129a–e. All the starting compounds were non-mesogenic in nature. Using POM, they studied the aggregations 127/129a–e at a scanning rate of 5 °C min^−1^. This study revealed that only the 127/129e complex exhibited liquid crystalline properties (*i.e.* it exhibited monotropic smectic A phase). Also, different behavior was observed in the *ortho*-difluorinated azobenzenes 128a–b with stilbazoles (128a/129a–e and 128b/129a–e) compared with the 127/129a–e complexes. Further, a decreased transition temperature by about *ca.* 50 °C from the crystalline state to the liquid-crystalline/isotropic state was observed compared to the 127/129a–e complexes. Enantiotropic mesophase was observed in the fluorine-based complexes; they exhibited mesomorphism in a broad range (∼50 °C) during the heating cycle, whereas during the cooling cycle, they exhibited mesomorphism in an even broader range (∼80 °C). As the alkyl chain length at the stilbazoles increased in the 128a- and 128b-based complexes, their transition temperatures from the liquid crystal state to the isotropic state also increased, except in the 128a/129c and 128b/129e complexes, where minute decreases in the clearing temperatures were noted compared to the complexes 128a/129a and 128b/129d, respectively. For the short-alkyl-chain complexes (129a and 129b), nematic phase was spotted; at the same time, smectic A phase was predominantly observed for the long-alkyl-chain complexes (129d and 129e). A dissimilarity was observed for the 129c complexes, which exhibited nematic phase when complexed with 128a and exhibited smectic phase when complexed with 128b. The typical texture of smectic E phase was noted in the 128a/129c, 128b/129c and 128b/129e complexes. Hence, this distinctly organized mesophase is structurally related to the smectic A phase; however, with the maintained orthorhombic lattice, it generally arises between the crystalline phase and smectic A phase. Further, they studied the photo-induced phase transition in the prepared complexes by illuminating with 405 nm LED and viewing *in situ* through POM. Upon illumination, the mesogenic alignment quickly vanished at 90 °C because of the photo-induced phase transition to isotropic phase. This phase transition was due to the *trans*–*cis* isomerization of the azobenzene group, and the mesophase destabilization was due to the bent structures of the *cis*-azobenzene groups. When the irradiation was halted, the mesogenic textures emerged immediately; within a few seconds, the textures were fully retrieved. Also, they obtained similar results when the experiments were performed even at 60 °C and 30 °C. Hence, these complexes can be utilized in photonics.

**Scheme 16:**
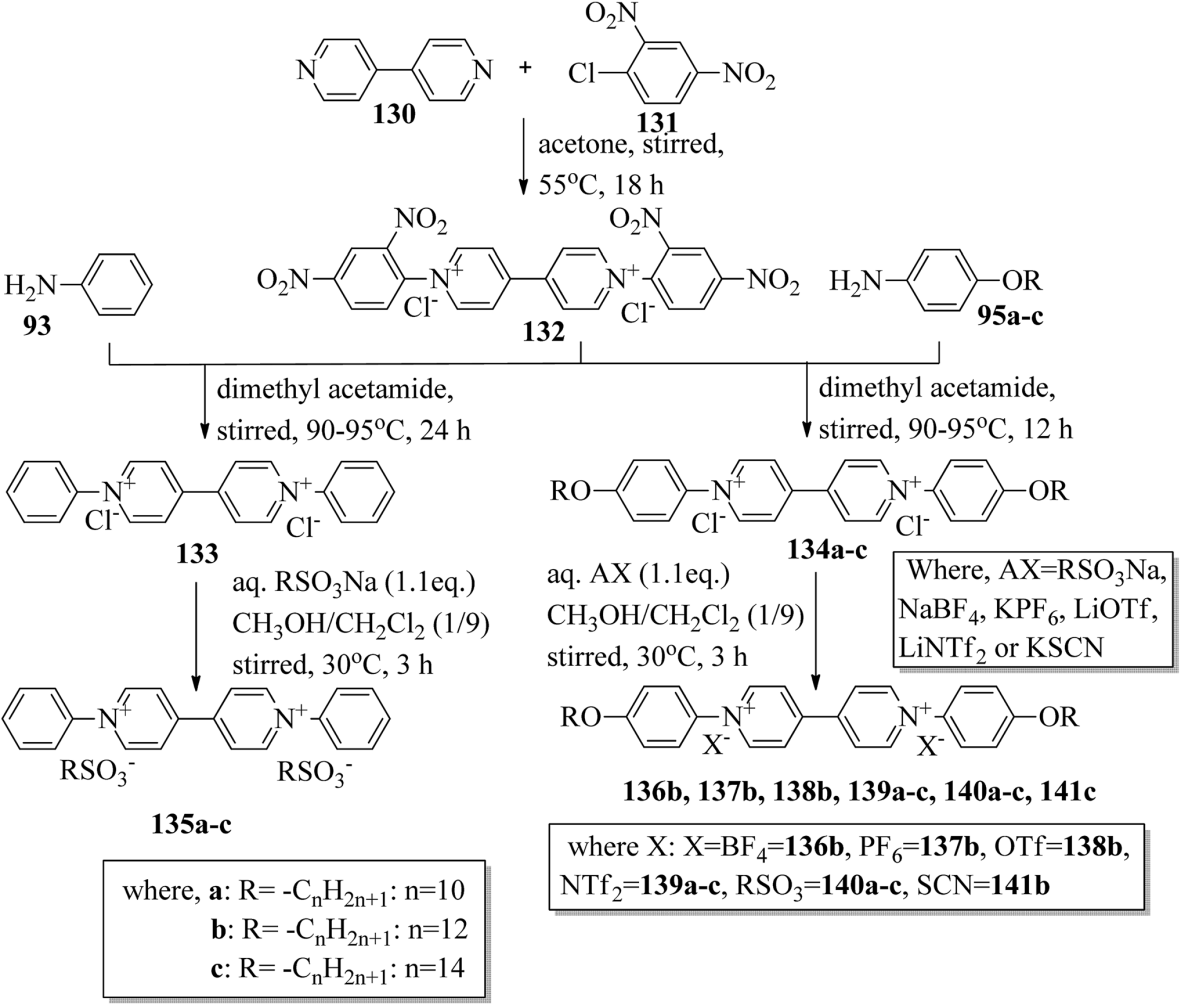
Synthesis of ionic liquids 135–141.

Wang *et al.*^59^ prepared two series of ionic liquid crystals from diphenylviologens. Further, they synthesized the precursors diphenyl viologen dichloride 133 and di(4-alkoxyphenyl)viologen dichlorides 134a–c (R = C_*n*_H_2*n*+1_, *n* = 10, 12, 14) through Zincke reactions by referring to previous literature reports by Nanasawa *et al.*^60^ and Kuo *et al.*^48^ During the metathesis of counter anions, methanol was used as the phase transfer atmosphere. The counter anion salts such as sodium tetrafluoroborate (NaBF_4_), lithium triflate (LiOTf), potassium thiocyanate (KSCN), sodium alkylsulfonate (RSO_3_Na), potassium hexafluorophosphate (KPF_6_), and lithium bis(trifluoromethane)sulfonimide (LiNTf_2_) were dissolved in water and added to a solution of methanol/dichloromethane (CH_3_OH/CH_2_Cl_2_ = 1/5) containing the precursors 133 or 134a–c. The reaction mixture was stirred for 3 h at 35 °C to 40 °C, and the reactions were monitored using alumina TLC plates with CH_3_OH/CH_2_Cl_2_ (5/95) eluent. Finally, the crude product was passed through an alumina (Al_2_O_3_) flash column, followed by recrystallization using an isopropanol/ethyl acetate mixture to afford the pure product (Scheme 16). The crystallographic data of two single crystals of 135a and 136b revealed that the presence of intermolecular hydrogen bonds induced the mono- or bilayer-lamellar structure. All the synthesized ionic liquids exhibited mesogenic behavior. Smectic A phase with a homeotropic texture or focal conic texture was generally observed during the cooling cycle. Powder XRD studies also revealed that smectic A phases exist as monolayer structures for 136–141 or bilayer structures for 135a–c. Further, the melting temperatures (*T*_mp_) remained at *T*_mp_ = 88.7 °C to 113.1 °C in the viologen 135a–c series; however, the clearing temperature (*T*_cl_) increased with the chain length, *i.e. T*_cl_ = 217 °C (135a) < 256 °C (135b) < 275 °C (135c). The extent of smectic A phase increased with the chain length during the cooling cycle: Δ*T*_SmA_ = 118.5 °C (135a) < 196.3 °C (135b) < 223.0 °C (135c). Smectic A phase was observed in viologen derivatives 136–141. However, the clearing point was found to be quite sensitive to the incorporated anions; *T*_cl_ = 337.0 °C (138b) > 336.0 °C (136b) > 312.0 °C (137b) > 304.8 °C (139b) > 274.0 °C (141b) > 220.2 °C (140b) for the *n* = 12 derivative. Substituted alkyl-chains at the anion and cation parts of compounds 140a–c displayed higher melting temperatures Δ*T*_mp_ = 31.7 °C (140a) < 43.8 °C (140b) < 50.0 °C (140c) and lower clearing temperatures Δ*T*_cl_ = 0.3 °C (140a) < 35.8 °C (140b) < 71.3 °C (140c) than compounds 135a–c. This alteration in both the melting and clearing temperatures results in a smaller smectic A phase range, *i.e.* Δ*T*_SmA_ = 92.4 °C (140a) to 86.0 °C (140c); this can be ascribed to the greater number of hydrophobic interactions than of hydrogen bond interactions and coulombic interactions. Similarly, in the viologen series 139a–c, the melting temperatures remained at 103.8 °C to 104.5 °C, but the clearing temperatures increased with carbon chain length, *i.e.*, *T*_cl_ = 254.3 °C (139a) < 304.8 °C (139b) < 328.0 °C (139c). The smectic A phase range increased with the carbon chain length during the cooling cycle: Δ*T*_SmA_ = 156.3 °C (139a) < 203.3 °C (139b) < 228.8 °C (139c). They concluded that elongation of the alkyl chain length increases the clearing and melting temperatures. In order to comprehend the effects of the counter anions on the mesogenic properties in diviologens, they prepared six compounds with different anions (X = BF_4_, PF_6_, OTf, NTf_2_, RSO_3_, SCN). All the prepared compounds exhibited mesogenic properties. They compared these bipyridinium-based mesogens with pyridinium-based mesogens (literature from Wang *et al.*^44^) and found that the bipyridinium-based mesogens exhibited wider Δ*T*_SmA_ ranges and higher *T*_cl_ values than the pyridinium-based mesogens. Decomposition before the clearing point was observed in the ionic salts with small anionic moieties, *i.e.* X = BF_4_, PF_6_, OTf, SCN. In compounds 136b and 141b, they noted more ordered smectic X phase, which was also confirmed using powder XRD analysis.

**Scheme 17:**
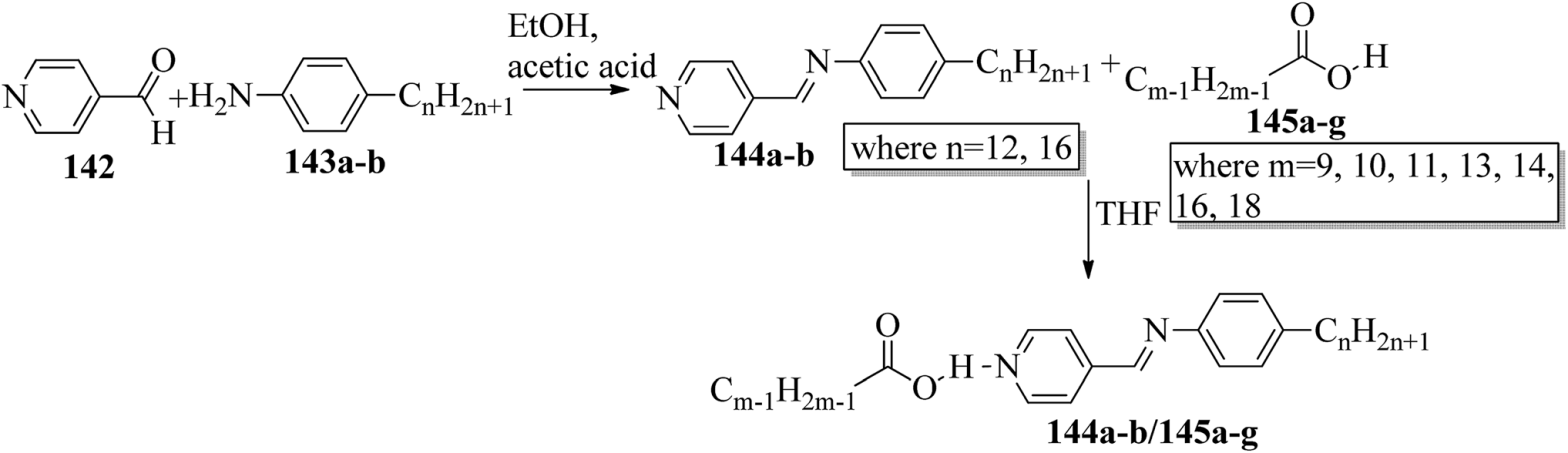
Preparation of hydrogen bonded complexes 144a–b/145a–g.

Bhat *et al.*^61^ prepared two series of hydrogen bonded liquid crystalline materials by mixing non-mesomorphic fatty acids such as nonanoic (145a), capric (145b), undecanoic (145c), tridecanoic (145d), myristic (145e), palmitic (145f) and stearic (145g) acids with (4-pyridyl)-benzylidene-*p-n*-alkylanilines (144a–b; *n* = 12 or 16). Condensation of 4-pyridine carboxaldehyde and the corresponding anilines, such as 4-*n*-dodecylaniline (143a) and 4-*n*-hexadecylaniline (143b), in the presence of a catalytic amount of glacial acetic acid yielded (4-pyridyl)-benzylidene-4′-*n*-alkylanilines 144a–b. The obtained products were recrystallized using ethanol solvent. The proton donors, *i.e.* fatty acids, and proton acceptors were dissolved separately in THF. The two solutions were mixed, refluxed for 1 h and stirred at room temperature overnight. Finally, the solvent was removed by distillation to form the expected hydrogen bonded complexes (Scheme 17). Using FT-IR spectroscopy, they confirmed the formation of hydrogen bonds between the fatty acids (145a–g) and (4-pyridyl)-benzylidene-4′-*n*-alkylanilines (144a–b). All the starting materials were non-mesogenic in nature; however, the prepared complexes exhibited monotropic smectic B phases. Further, they observed that the DSC thermograms of the 144b/145a and 144b/145d aggregations were folded in the direction of the high temperature side. This abnormal characteristic may arise because of the enormous amount of heat released, during which the sample pan temperature was higher than that of the reference pan. It was observed that fatty acid complexes with odd numbers of carbon atoms showed lower mesomorphic thermal ranges than the complexes of fatty acids with even numbers of carbon atoms. The higher thermal ranges of the mesogenic phases in complexes with even-numbered carbon atoms were ascribed to the strong intermolecular interactions which were caused by the large surface areas of the long chains as well as to the beneficial longitudinal dipole moments of the molecules along the long molecular axes of the hydrogen bonded complexes. The thermal ranges of the mesogens of the 144b/145a–g series were found to be slightly lower than those of the 144a/145a–g series. This is because of the long chain length of the proton acceptor, where the projection of the end chain dilutes the core of the molecule and causes disorder in the orientation. In both series, they observed that the clearing temperatures increased as the number of carbon atoms in the fatty acids increased. This was attributed to the increasing molecular weight of the resulting hydrogen bonded complexes. They also compared the mesogenic properties with their previous report (Bhat *et al.*^62^) and concluded that the π⋯C–H interactions and π–π stacking interactions play crucial roles while exhibiting mesomorphic properties. Smaller thermal ranges of mesomorphic phase than those of the pure alkoxy benzoic acid dimeric mesogens were observed in the 144a–b/145a–g complexes with two aromatic rings.

**Scheme 18:**
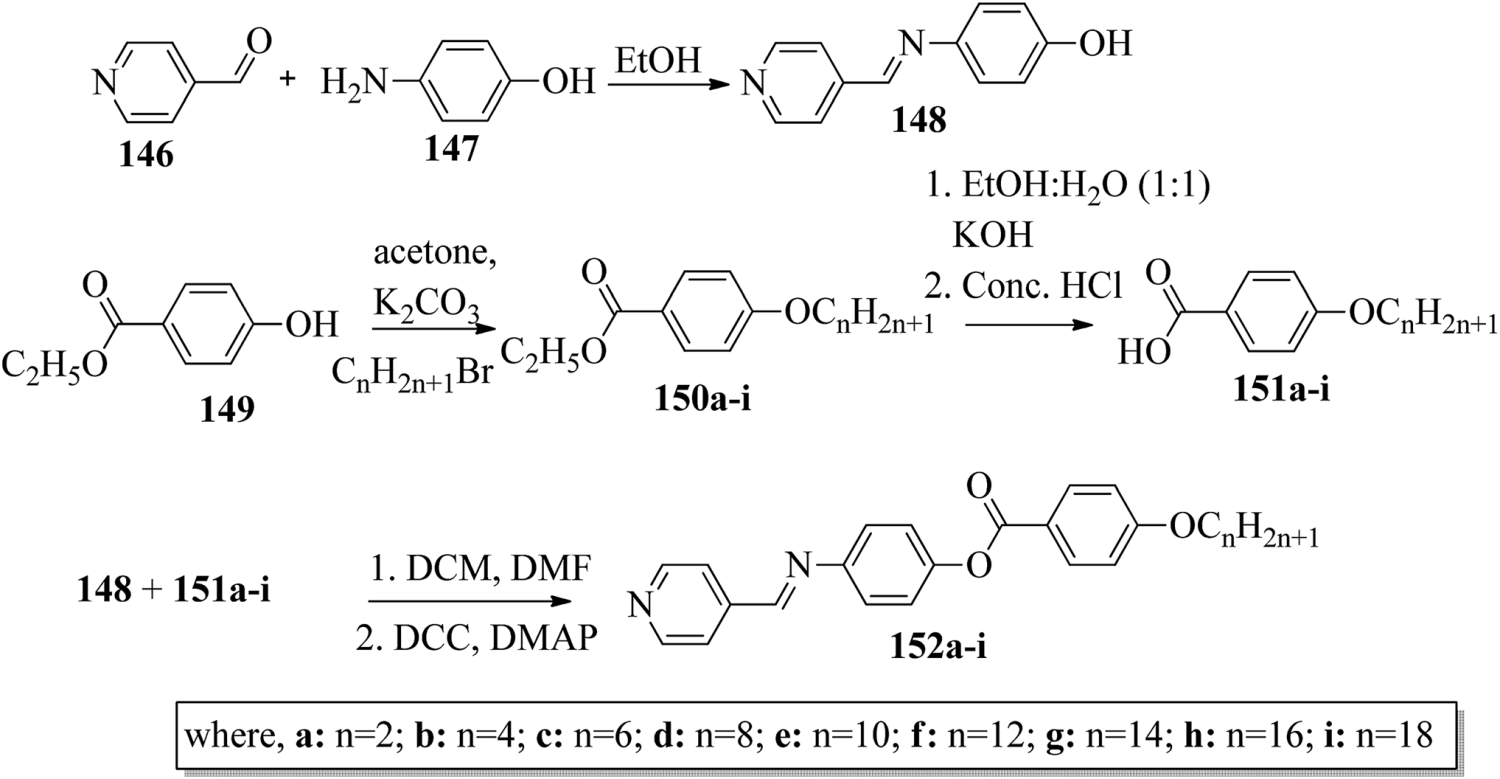
Synthetic route for 152a–i.

**Fig. 12:**
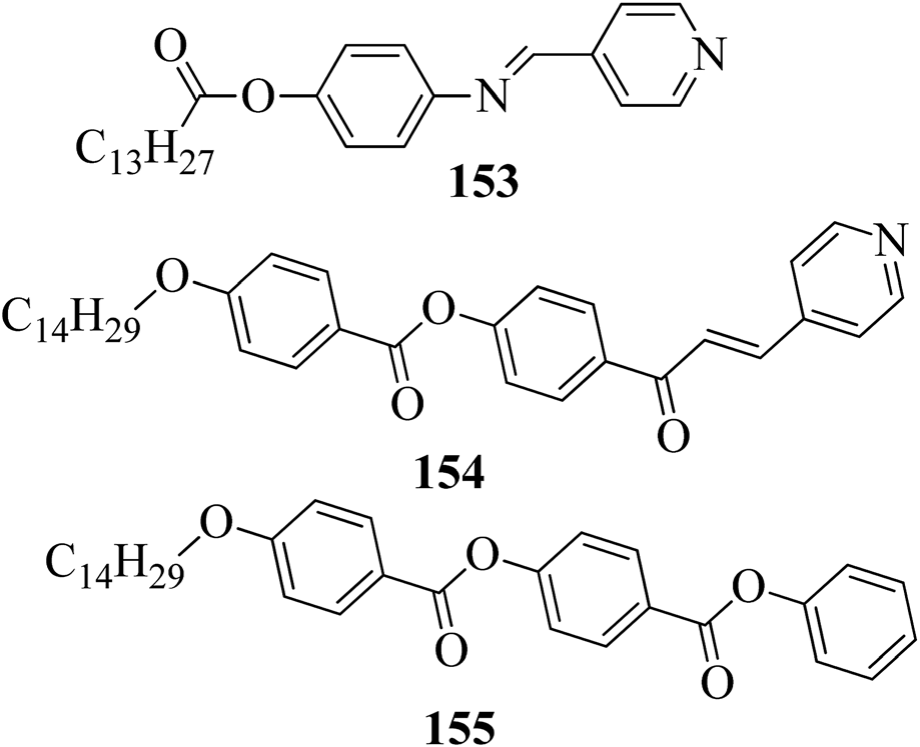
Structures of 153, 154, and 155.

Ong *et al.*^63^ synthesized calamitic mesogens, 4-{[(pyridin-4-yl)methylidene]amino}phenyl 4-alkoxybenzoates (152a–i), containing pyridine heterocycles and a two-phenyl core ring system, imine linkage, ester linkage, and alkoxy chain (which was varied as *n* = 2, 4, 6, 8, 10, 12, 14, 16, 18). Further, they referred to previous reports by Ha *et al.*^64^ and Kadkin *et al.*^65^ to synthesize the compounds 4-[(pyridine-4-ylmethylene)amino]phenol (148) and 4-alkoxybenzoic acid (151a–i), respectively. Compounds 148, 151a–i, and DMAP were dissolved in a mixture of DMF and DCM solvents, and the mixture was stirred at 0 °C. Further, the required amount of DCC was dissolved in DCM and added dropwise to the reaction mixture, which was then stirred at 0 °C for an hour. The mixture was later stirred for 3 h at room temperature. Finally, the reaction mixture was filtered and the solvent was evaporated to obtain the expected products (Scheme 18). The first three compounds, *i.e.*152a–c (*n* = 2, 4 and 6) in the series exhibited nematic mesophase. In addition to nematic phase, smectic A phase was also observed as the alkoxy chain length increased to *n* = 8 and *n* = 10. However, when the alkoxy chain length moved from *n* = 12 to *n* = 18, the nematic phase vanished and only smectic A phase was observed in these complexes. Ester linkages provide greater dipole–dipole interactions, which influences the lateral packing; due to this, smectic phase was generated. Hence, additional smectic A phase was observed along with nematic phase as the carbon chain length increased to C-8. The melting temperatures decreased as the chain length varied from C-2 to C-12 and further increased with increasing chain length from C-12 to C-18. However, the clearing temperature decreased throughout the series because long carbon chains cause dilution of the mesogenic core. The smectic phase range increased as the chain length increased from C-8 and reached the highest mesophase range with C-12; thereafter, the smectic A phase range decreased as the chain length increased from C-14 to C-18. Hence, the widest smectic A phase range was observed in 152f (ΔSmA = 51.3 °C) and the widest nematic phase was observed in 152a (Δ*N* = 53.0 °C). They also compared these compounds with previously reported compounds, *i.e.*153 (Ha *et al.*^64^), 154 (Lim *et al.*^66^) and 155 (Sakurai *et al.*^67^). The structures of 153, 154, and 155 are presented in Fig. 22. Compound 153 with a pyridine ring and aromatic ring did not display mesogenic properties although the two rigid rings were connected by a Schiff base group. When another rigid aromatic ring was added to this compound, it became 152g (the title compound). The addition of this rigid aromatic ring increases the length of the molecule and does not change the width of the compound. Hence, they concluded that for the generation of mesophases, this longer molecule was more suitable. Also, the stability of the phases was found to be higher for the three-ring molecule (152g) than for the two-ring molecule (compound 153). Next, they compared 152g with the three-ring system 154; the connecting unit between the aromatic ring and pyridine ring was the only difference between these two compounds. Compound 154 possesses an enone as the linking unit, whereas a Schiff base is the linking group in 152g. A lower melting point of about 99.2 °C and a wider smectic A phase range of about ΔSmA = 43.8 °C were observed in 152g compared to compound 153, in which a melting point of about 109.8 °C and a smectic A phase range of about ΔSmA = 7.3 °C were observed. The molecular linearity of the title molecule was maintained, although it has a stepped core structure due to the Schiff base linkage; therefore, the compound induces mesophase formation and provides better stability. Hence, they concluded that the Schiff base connecting group performs a notable role in minimizing the melting temperature and then broadens the range of the mesophases. Compound 154 also contains three aromatic rings connected by ester linkages. This aromatic-ester compound exhibited a smectic A phase range of about 20 °C from the starting temperature of 113 °C. When the aromatic-ester complex was changed to a pyridine-Schiff base complex, *i.e.* molecule 152g, its melting temperature decreased; accordingly, this broadened the mesophase range. Hence, they concluded that the superior mesogenic properties in 152g were contributed by the pyridine nucleus complex and Schiff base linking unit (Fig. 12).

**Scheme 19:**
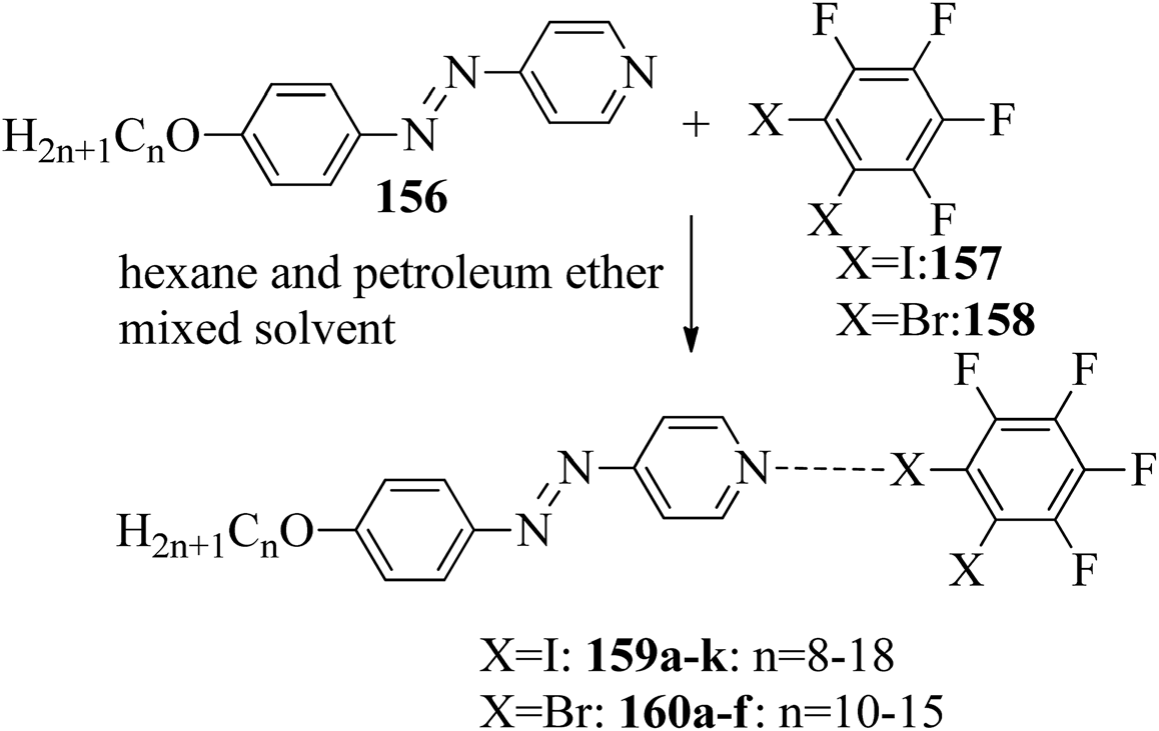
Preparation of halogen bonded complexes 159a–k and 160a–f.

Du *et al.*^68^ prepared halogen bonded complexes using 1,2-dibromotetrafluorobenzene (158) or 1,2-diiodotetrafluorobenzene (157) and azopyridine derivatives (156). Halogen bond acceptors, *i.e.* azopyridine derivatives (156), were synthesized by diazotization and azo coupling reactions. The authors used commercially available halogen bond donors 157 and 158. Further, they prepared supramolecular complexes by mixing equimolar ratios of halogen-bonded acceptor 156 and halogen-bonded donors 157 or 158 in *n*-hexane and petroleum ether mixed solvent. The mixture was stirred for 7 to 8 h at room temperature. Finally, the expected complexes were obtained by evaporating the solvents (Scheme 19). Monotropic mesophases were observed in the iodine-bonded complexes 159c–h (*n* = 10 to 15), *i.e.* on heating, the complexes directly melted to the isotropic state from the crystalline state; however, during the cooling cycle they exhibited the focal conic fan texture of smectic A phase in the temperature range of about 45 °C to 30 °C. Further, they prepared the bromine-bonded complexes for comparison with the iodine-bonded complexes. Unfortunately, the bromine-bonded complexes did not exhibit any significant mesogenic character; this may be because of the weak N⋯Br interactions in the liquid state, which is similar to a previous literature report of halogen-bonded aggregates of 1,4-dibromotetrafluorobenzene and alkoxystilbazoles (González *et al.*^69^). To study the photo-induced phase transitions in the prepared complexes, further, they illuminated them with UV light of 365 nm (80 mW cm^−2^). Upon illumination at 41 °C, the birefringent smectic A phase vanished after 3 s because of the photo-induced phase transition to the isotropic phase. This phase transition is due to the *trans*–*cis* isomerization of the azopyridine group, and the mesophase destabilization was due to the bent structures of the *cis*-azopyridine groups. When the irradiation was halted, mesogenic textures emerged.

**Scheme 20:**
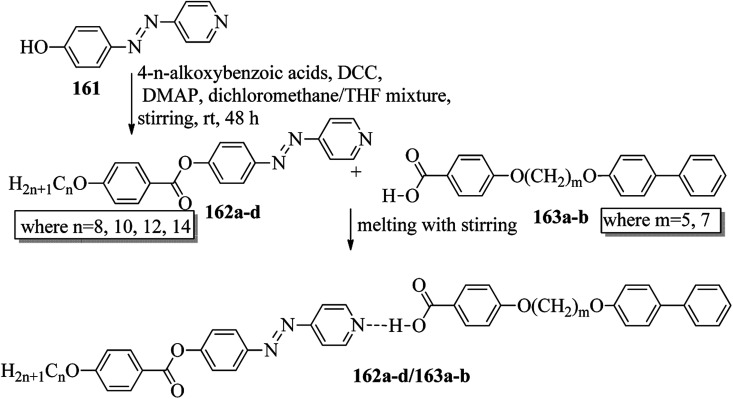
Preparation of hydrogen bonded complexes 162a–d/163a–b.

Alaasar and Tschierske^70^ prepared hydrogen bonded supramolecular mesogenic complexes using 4-(4′-pyridylazophenyl)-4′-alkoxybenzoates (162a–d) as proton acceptors and 4-[5-(4′-cyanobiphenyl-4-yloxy)alkyloxy]benzoic acids (163a–b) as proton donors. According to the synthetic route given in a previous report by Naoum *et al.*,^71^ they synthesized the pyridine-based components (162a–d) by esterification reactions between 4-*n*-alkoxybenzoic acids and 4-(4′-pyridylazo)-phenol (161). By referring to previous publications, *i.e.* reports by Alaasar and Tschierske,^72^ they synthesized the proton acceptor compounds (163a–b). The non-reported compound 4-(4′-pyridylazophenyl)-4′-octyloxybenzoate (162a) was synthesized by stirring the compounds 161, octyloxybenzoic acid, DCC, and DMAP catalyst in a mixture of CH_2_Cl_2_ and THF (0.75 : 0.25) for 48 h at room temperature. The obtained solid was filtered and washed with CH_2_Cl_2_. After removal of the solvents under vacuum, the product was purified by column chromatography using CH_2_Cl_2_ as the eluent. The expected complexes were prepared by melting 162a–d and 163a–b in an equimolar ratio with stirring. The solid obtained after cooling was ground and the process was repeated until a homogeneous mixture formed (Scheme 20). Initially, they studied the mesogenic properties of the pure parent compounds; this study revealed that the transition temperatures of compound 163a are in good accord with the same compounds previously reported by Jansze *et al.*^73^ They observed slightly different transition temperatures of the pyridine-based components (162b–d) than the same compounds which were previously reported by Naoum *et al.*;^71^ hence, they reported new transition temperatures in addition to those of the non-reported pyridine-based component 162a. The prepared acids (163a–b) exhibited enantiotropic nematic phase, which was ascribed to dimerisation of the acid molecules. The short chain pyridine-based components (162a–b) exhibited nematic and smectic A phases; however, as the chain length increased (162c–d), only smectic A phase was observed by quenching the nematic phase. The intermolecular hydrogen bond formation between the acid and base elements induces broad-ranged nematic phases by quenching the smectic A phases of the pyridine-based derivatives in all the prepared complexes. The nematic phase was extended over a wide range of temperature; this range even overshot ∼75 K for most of the aggregates and reached ∼90 K for 162b/163b. This indicates that in these supramolecular structures, the nematic phase is stabilized by hydrogen bonding. For all the prepared aggregations, the nematic mesophases could be super-cooled prior to crystallization, with the highest value of ∼48 K for 162d/163b and the lowest value of ∼23 K for 162a/163b.

**Scheme 21:**
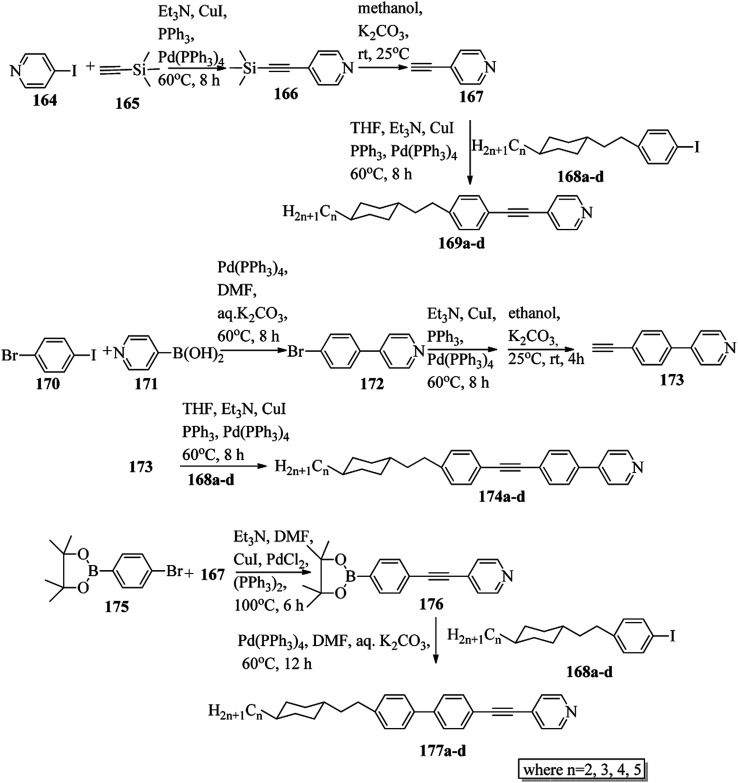
Synthesis of 177a–d.

Chen *et al.*^50^ synthesized mesogens with 4-pyridyl end groups containing phenylenes and one triple bond. The compounds 4-(2-(*trans*-4-*n*-alkylcyclohexyl)ethyl)iodobenzene (168a–d) were synthesized according to the previous literature strategy used by Chen *et al.*^74^ To a suspension of Pd(PPh_3_)_4_, 4-iodopyridine (164), PPh_3_, and CuI in dry Et_3_N and THF, ethynyltrimethylsilane (165) in dry Et_3_N was added dropwise at room temperature. The reaction mixture was stirred at 60 °C for 8 h under nitrogen protection. After that, it was cooled and filtered; then, DCM solvent was added and the organic layer was washed using saturated ammonium chloride solution. Later, the solution was dried using anhydrous MgSO_4_ and the solvents were evaporated. The obtained crude product was directly utilized for the next steps. This crude product was dissolved in methanol and taken in a nitrogen-flushed round-bottom flask; then, K_2_CO_3_ was added. The reaction mixture was stirred for 3 h and filtered through Celite. Then, the solvent was dried under reduced pressure to yield 4-ethynylpyridine (167), and it was further purified by column chromatography using petroleum ether as the eluent. To a mixture of K_2_CO_3_, water and 4-pyridineboronic acid (171) in DMF, 1-bromo-4-iodobenzene (170) in DMF was added. This reaction mixture was freed from unwanted gas using N_2_ for 10 min, and then Pd(PPh_3_)_4_ was added to it. This reaction mixture was maintained with stirring under N_2_ at 60 °C for 15 h. After the reaction was complete, the solvent was evaporated using a rotary evaporator; then, the precipitate was dissolved in CH_2_Cl_2_ solvent and washed with water and brine solution. The organic layer was then dried using magnesium sulphate, filtered and concentrated to yield 4-(4-bromophenyl)pyridine (172). Similar to the synthetic process of 166, compound 172 was used as the raw material to afford the product 4-(4-((trimethylsilyl)ethynyl)phenyl)pyridine. Then, this product was dissolved in methanol, and K_2_CO_3_ was added to it. The reaction mixture was then stirred for 4 h, and the reaction mixture was extracted using DCM, washed with brine and water, and dried using MgSO_4_. Under reduced pressure, the solvent was evaporated to obtain the compound 4-(4-ethynylphenyl)pyridine (173). A suspension of PdCl_2_(PPh_3_)_2_, 2-(4-bromophenyl)-4,4,5,5-tetramethyl-1,3,2-dioxaborolane (175), and CuI in a mixture of dry Et_3_N and DMF was prepared; to this, 4-ethynylpyridine (177) in dry Et_3_N was added dropwise, and stirring was maintained at 100 °C for 8 h. After the reaction was complete, the solvent was removed to obtain 4-((4-(4,4,5,5-tetramethyl-1,3,2-dioxaborolan-2-yl)phenyl)ethynyl)-pyridine (176) as the product. A mixture of compound 177, THF and Et_3_N was prepared in a two-way flask and bubbled with N_2_ gas. 4-(2-(*trans*-4-*n*-Propylcyclohexyl)ethyl)iodobenzene, CuI, Pd(PPh_3_)_4_, THF were taken in another two-way flask, which was filled with N_2_. To this reaction mixture, they added the previous liquid mixture. Later, this reaction mixture was stirred for 8 h at 60 °C. They used saturated ammonium chloride solution to remove CuI from the reaction mixture. Using water, the mixture was diluted and extracted with ethyl acetate. The organic layer was then dried using MgSO_4_ and the solvents were evaporated in vacuum to yield 4-((4-(2-(*trans*-4-*n*-propylcyclohexyl)ethyl)phenyl)ethynyl)pyridine (169b); further, it was purified by column chromatography. Also, the authors synthesized 4-(4-((4-(2-(*trans*-4-npropylcyclohexyl)ethyl)phenyl)ethynyl)-phenyl)pyridine (174b) similarly to 169b by replacing 167 with 173. To a mixture of K_2_CO_3_ in water and 176 in DMF, they added 168b in DMF. Using N_2_, this solution was degassed for 10 min, and then Pd(PPh_3_)_4_ was added to it. This reaction mixture was maintained with stirring for 12 h under N_2_ at 60 °C to obtain the product 4-((4′-(2-(*trans*-4-npropylcyclohexyl)ethyl)-[1,1′-biphenyl]-4-yl)-ethynyl)pyridine (177b). Using similar procedures, the other target compounds were synthesized (Scheme 42). From the study of these synthesized compounds, they observed that compounds 169a–d exhibited nematic phase ranges of 8.8 °C to 17.9 °C and 9.1 °C to 39 °C on heating and cooling, respectively, while compounds 174a–d exhibited ranges of 82.9 °C to 95.5 °C and 83.7 °C to 111.5 °C on heating and cooling, respectively, and compounds 177a–d exhibited ranges of 73.6 °C to 77.8 °C on heating and cooling, respectively. The small molecular length-to-width ratios led to non-mesogenic natures of compounds 169a–d with short terminal chains (*n* < 4). The clearing temperatures of compounds 169a–d increased with increasing terminal alkyl chain length (*n* = 4, 5), and the melting temperatures of compounds 169a–d displayed an even–odd effect: higher melting temperatures were observed for compounds 169a–d with odd terminal alkyl chains (*n* = 3, 5) than for those with even terminal alkyl chain lengths (*n* = 2, 4). At the same time, compound 174a exhibited a lower clearing point and melting point than compound 174b, but the longer terminal chain lengths (*n* = 4, 5) showed lower melting and clearing temperatures. Also, they observed that the alkyl chain length exhibited a small impact on the clearing and melting points of compounds 177a–d. In addition, an opposite even–odd effect was observed in the clearing and melting points of compounds 177a–d. They also investigated the impact of another phenyl rigid core on the mesogenic properties. The stability of the nematic phase was improved by adding a phenyl rigid core to the mesogen core to afford compounds 174b and 177b, in which the nematic phase ranges increased from 0 °C to 94.7 °C and 0 °C to 77.9 °C, respectively. Further, these results revealed that the introduction of another phenyl rigid system induces nematic phase formation and enhances the nematic phase interval. Moreover, there is a 38.3° twist in the biphenyl rings of compound 177b and twist angles of about 36.9° in the phenyl and pyridine rings in compound 174b; this influences the broadening of the nematic mesophase intervals. In addition, nematic mesophase stability was favored by the adequate dipole–dipole interactions of compound 174b (Scheme 21).

**Scheme 22:**
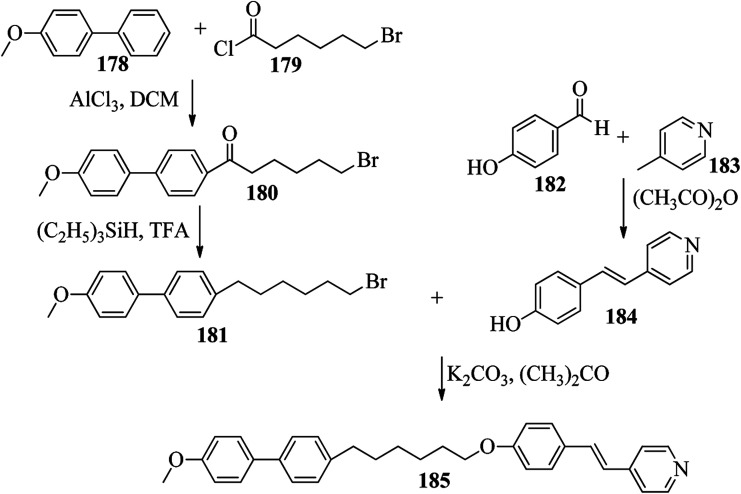
Synthesis of 185.

**Scheme 23:**
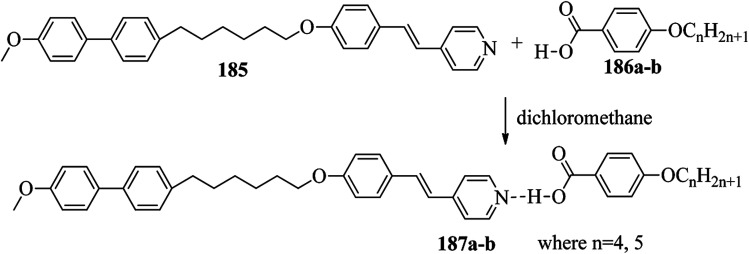
Preparation of hydrogen bonded complexes 187a and b.

Walker *et al.*^75^ prepared hydrogen bonded complexes using alkoxy benzoic acids (186a–b) and a stilbazole-based template (185) as the proton donor and acceptor, respectively. Referring to the procedures described by Abberley *et al.*,^76^ they synthesized the compound 4-(6-bromohexyl)-4′-methoxy-1,1′-biphenyl (181). Then, they prepared 4-[(*E*)-2-(pyridin-4-yl)ethenyl]phenol (184) by refluxing a reaction mixture containing 4-methylpyridine (183) and 4-hydroxybenzaldehyde (182) in acetic anhydride for 23 h. After completion of the reaction, the reaction mixture was cooled to room temperature, poured into ice water and stirred for 1 h. The precipitate was filtered and refluxed for 2 h in the presence of alcoholic potassium hydroxide. To obtain compound 184 as a precipitate, they added acetic acid; the precipitate was then recrystallized using ethanol solvent. Compound 184, compound 181 and potassium carbonate were dissolved in acetone and refluxed for 96 h at 65 °C. After completion of the reaction, the reaction mixture was cooled to room temperature; the formed precipitate was filtered and purified using column chromatography to yield 4-[(*E*)-2-(4-{[6-(4′-methoxy[1,1′-biphenyl]-4-yl)hexyl]oxy}phenyl)ethenyl]pyridine (185). The synthetic route for compound 185 is illustrated in Scheme 22. Finally, the expected hydrogen bonded binary mixtures were prepared by dissolving equimolar ratios of 185 and 186a or 186b in dichloromethane, and the solvent was evaporated at room temperature. Then, the mixture was maintained in a vacuum oven for 16 h at 50 °C for drying (Scheme 23). The aggregations exhibited various mesophases across a wide range of temperatures even though the parent molecule 185 is a non-mesogen and 186a–b, with *n* = 4 or 5, display only narrow temperature ranges of achiral nematic mesophase. During the cooling cycle, the 187a aggregation displayed two nematic mesophases that were differentiated by their optical textures: in cells with planar securing, the higher-level temperature nematic mesophase displays an absolutely uniform texture, and at the lower transition temperature of mesophase, a stripe-like texture develops in typical twist-bend nematic mesophase. The emergence of the twist-bend nematic mesophase was accompanied by the termination of the optical glimmering ascribed to the director variations noted in the nematic mesophase. The nematic to twist-bend nematic mesophase transition was observed in DSC analysis as a minute leap in the heat capacity. This was in agreement with the normal result that as the temperature range preceding nematic mesophase increases, the nematic to twist-bend nematic mesophase transition tends to become second order in nature, *i.e.* Δ*H* ≈ 0. In thin cells, the sample crystallization was arrested and a reversible twist-bend nematic mesophase to smectic X transition was observed. Unfortunately, the monotropic character of this phase hindered its analysis using XRD; however, given the high enthalpy variation linked with the transition, it was presumed to be a smectic phase of high order. For the 187b aggregation, the temperature range of the twist-bend nematic mesophase decreased to ∼5 K, which was inadequate for the development of the stripe-like texture. Further, smectic A and smectic X phases were noted below the twist-bend nematic mesophase.

**Scheme 24:**
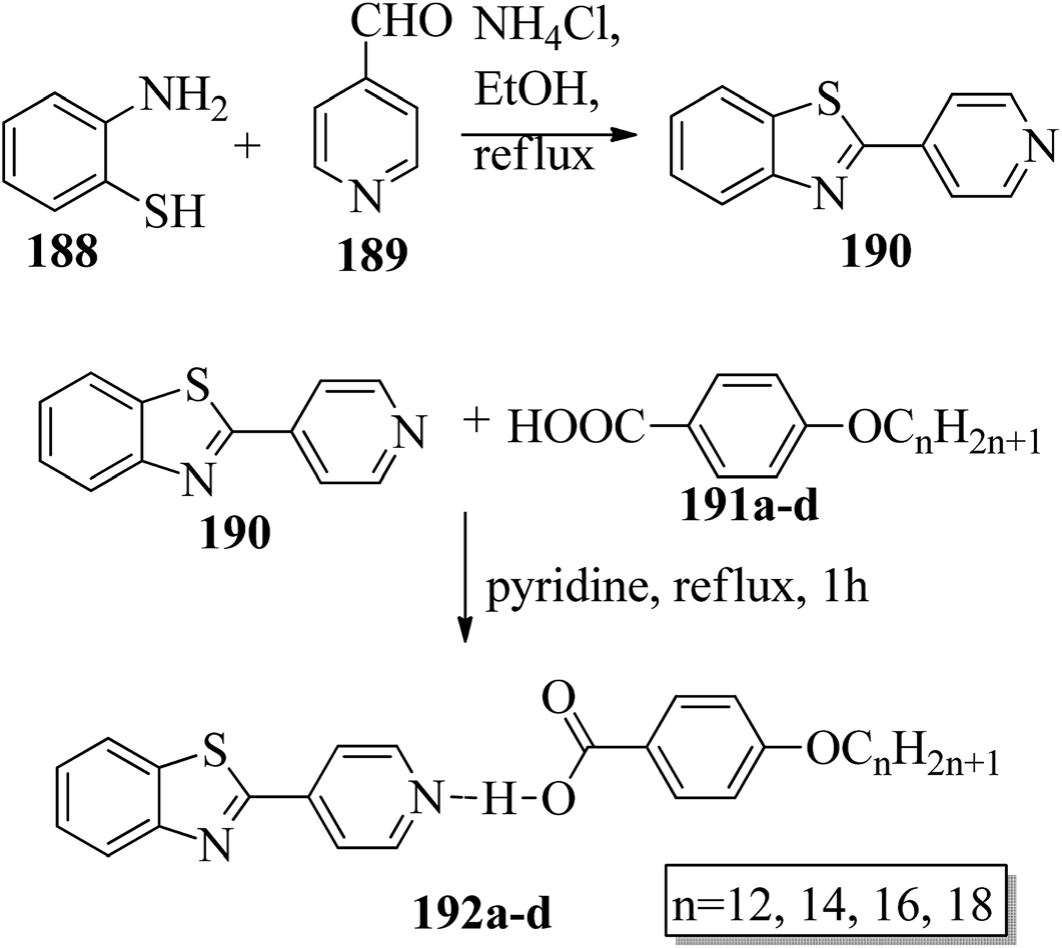
Preparation of hydrogen bonded complexes 192a–d.

Chakraborty *et al.*^77^ prepared hydrogen bonded complexes using alkoxy benzoic acid as the proton donor and pyridenyl benzothiazole as the proton acceptor. The synthetic route for the preparation of the hydrogen bonded complexes is illustrated in Scheme 24. The derivative of benzothiazole, *i.e.* 2-(pyridin-4-yl)benzothiazole (190), was synthesized by an altered strategy based on the previous literature report by Maleki and Salehabadi.^78^ According to this procedure, the *o*-amino thiophenol (188), aldehyde (189), and ammonium chloride catalyst were refluxed in ethanol solvent. On the other hand, the hydrogen donors, *i.e.* alkoxy benzoic acids (191a–d) with different chain lengths, were synthesized by necessary modification of the strategy described by Spring *et al.*^79^ During synthesis, *para*-hydroxyl benzoic acid, alkyl bromide, and KOH were refluxed in ethanol, followed by acidification using HCl solution. Finally, the expected hydrogen bonded aggregations were prepared by dissolving equimolar amounts of 2-(pyridin-4-yl)benzothiazole (190) and alkoxybenzoic acids (191a–d) in pyridine and refluxing them. The solvent (pyridine) was reduced and removed in vacuum, and the obtained product was dried using a desiccator (Scheme 24). Among the prepared complexes, only 192a displayed monotropic nematic mesophase on super-cooling. The complexes displayed notably lower emergence temperatures for the nematic and isotropic phases compared to the parent alkoxy benzoic acids. None of the parent alkoxy benzoic acids displayed fluorescence properties when scanned from the crystalline state to mesogenic phase to isotropic phase. Interestingly, the hydrogen bonded complex 192a displayed phase-dependent fluorescence. The aggregates displayed green fluorescence in the crystalline state upon excitation (Ex 300 to 360 nm filter). During the heating cycle, complex 192a underwent a transition from liquid crystalline to isotropic phase at 97 °C with completely quenched fluorescence behaviour. During the cooling cycle, moderately increased order in the arrangement of the molecules was observed during the liquid to nematic phase transition stage as well as the appearance of exceptionally low-intensity red fluorescence. Upon further cooling from nematic mesophase to the crystalline state, the complex displayed bright green fluorescence. Also, further heating/cooling of this mesogen exhibited OFF/ON fluorescence switching. In the crystalline phase, green fluorescence was observed, which is due to the highly ordered alignment of the compound supplied by the strong hydrogen bonds between 190 and 191a. Moderate collapse of the hydrogen bonding between 190 and 191a occurs at high temperature, which causes the intense change in luminescence from bright green to exceptionally low-intensity red fluorescence or non-luminescence.

Goossens and co-workers^80^ published a review article on the design, synthesis, and characterization of certain pyridinium and 4,4′-bipyridinium (viologen)-based thermotropic ionic liquid crystals and also highlighted their applications. Further, Bhowmik *et al.*^81^ prepared a series of symmetric viologen triflimides obtained by reacting viologen tosyalates with lithium triflimide through metathesis reactions. Furthermore, they noted relatively low melting points of viologen salts that bear two or three carbon atoms in their alkyl chains. In addition, viologen salts carrying alkyl chains with four or five carbon atoms showed ionic liquid behavior at 88 °C and 42 °C, respectively. Also, the salts of alkyl chains comprising nine, ten or eleven carbon atoms formed high-melting salts. However, mesogenic phases such as smectic C, smectic A and an unidentified smectic phase (smectic X) were observed only in the viologen salts with higher alkyl chain lengths (*i.e.* 16, 18 and 20 carbon atoms). In another report (Bhowmik *et al.*^82^), a series of viologens composed of 4-*n*-alkylbenzenesulfonates were prepared by respective metathesis reactions between viologen dibromide and sodium 4-*n*-alkylbezenesulfonates or 4-*n*-alkylbenzenesulfonic acids. These ionic molecules exhibited focal conic or Schlieren textures (smectic A phases) and undefined smectic X phases with good thermal stability.

Casella and co-workers^83^ synthesized symmetric viologen dimer salts with bistriflimides as counter-ions and investigated the reliance of the mesogenic properties on the lengths of both the lateral and spacer chains. An ordered smectic mesophase (smectic X) was noted for all the compounds. In addition to this, fluid smectic A mesophase was observed when the spacer chain contained less than six carbon atoms and the lateral chains contained more than twelve carbon atoms. Then, Pibiri *et al.*^84^ prepared two different types of ionic mesogens comprising non-symmetrically substituted polyfluorinated viologen salts (linear molecules) and bent-shaped symmetrically substituted dialkyl-oxadiazolyl-bipyridinium salts of bistriflimide ion. Most of the linear molecules displayed smectic X mesophase; in some of the molecules, smectic A mesophase was observed in addition to smectic X mesophase. Moreover, the inserted bent oxadiazolyl spacer between the two pyridinium moieties significantly altered the mesophase properties and exhibited dendritic textures of the banana mesophases.

### 2.2. Pyridine-based discotic mesogens

Discotic liquid crystals are disc-like molecules where the molecules are stacked one above the other to form columns. Discotic nematic and columnar mesophases are the two main mesophases that are generally observed in this type of mesogen. In the case of discotic nematic mesophase, the molecules possess no positional order but have a least orientational order. On the other hand, the arrangement of molecules in the columns of different lattices gives rise to different types of columnar mesophase patterns, such as columnar rectangular and columnar hexagonal mesophases. This section covers the available synthetic roots for the preparation of different pyridine-based discotic/columnar mesogens and their properties.

**Scheme 25:**
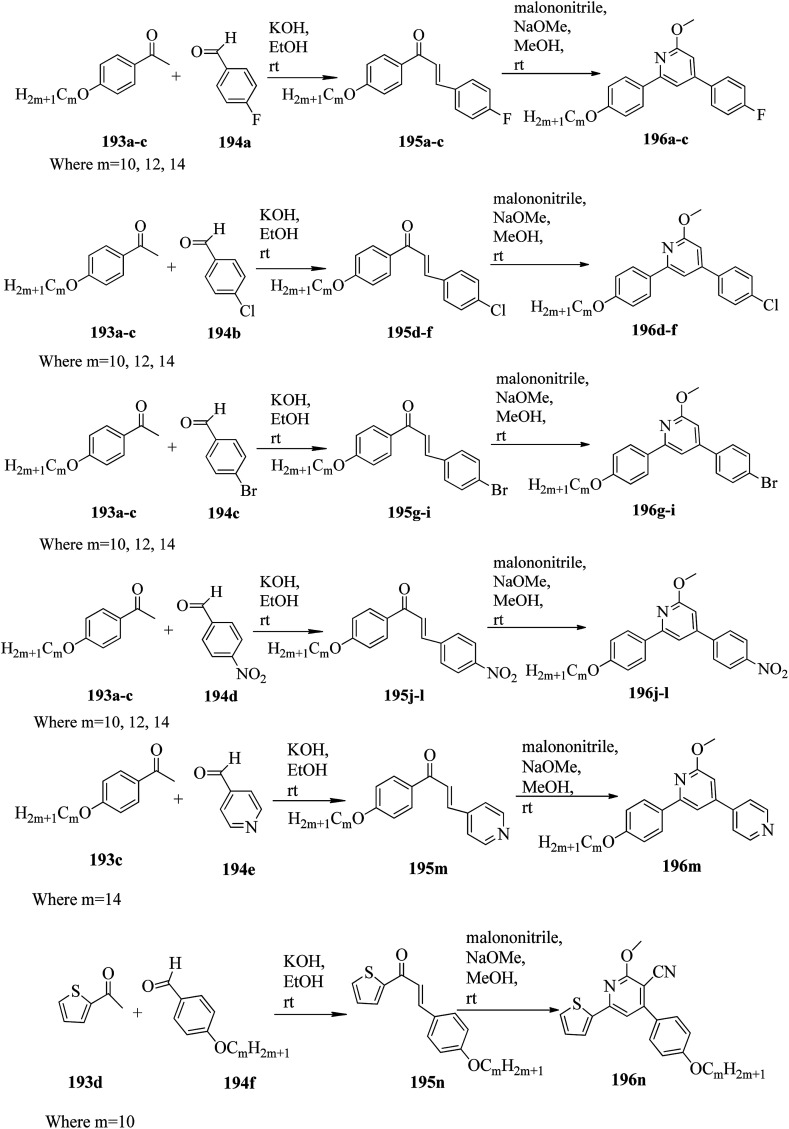
Synthetic route for the fourteen blue luminescent mesogens 196a–n.

Ahipa and Adhikari^85^ synthesized fourteen new blue luminescent mesogens with a core unit of 2-methoxy-3-cyanopyridine or 2-methoxypyridine and variable substituted aryl/heteroaryl rings as well as alkoxy phenyls as terminal substituents. The synthesis of the target compounds involves the Claisen–Schmidth reaction, *i.e.* chalcone formation by reacting ketone (193a–d) and aldehyde (194a–f) compounds in ethanol solvent in the presence of base (KOH) followed by stirring the reaction mixture for 4 h at room temperature in order to obtain the crude chalcones (195a–n), which are later purified by recrystallization using ethanol solvent. Further, the prepared chalcones were added slowly to freshly prepared sodium methoxide solution and stirred. Then, malononitrile was added, with continuous stirring at room temperature until precipitation of the products (196a–n). Later, the crude products were filtered and washed with methanol, then recrystallized using ethanol (Scheme 25). Further, photophysical studies indicated that these compounds are promising blue emissive materials. Single crystal studies revealed the bent-shaped structure of these compounds, with a marginal non-planar arrangement and various intermolecular interactions. Also, these compounds exhibited liquid crystalline properties, which was confirmed by POM, DSC, and powder XRD techniques. In these compounds, the wide thermal ranges of the mesogens were influenced by the nature of the polar substituents. Nematic phase was also observed in the compounds with a lateral –CN group attached to the pyridine core and a terminal –F or –Cl substituent. In the presence of the terminal –NO_2_, –Br or 4-pyridyl group and in the absence of the lateral –CN group in the pyridine core, the formation of a rectangular columnar phase was observed. Finally, they concluded that these molecules are emerging blue emissive mesogens with good charge transporting ability and can be utilized further for applications in optoelectronics.

**Scheme 26:**
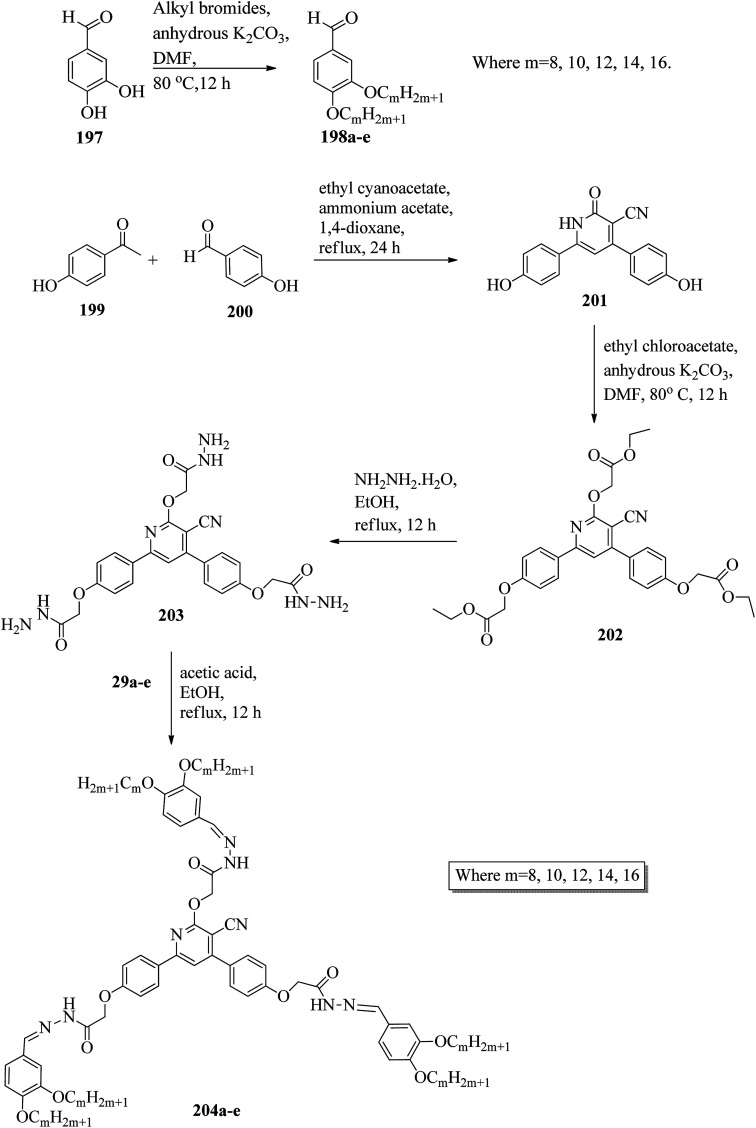
Synthetic route for the series of cyanopyridine derivatives 204a–e.

In another report, Ahipa and Adhikari^86^ synthesized a series of cyanopyridine derivatives 204a–e carrying a 3,4-dialkoxyphenyl group and studied their optical and mesogenic properties. The synthetic route involves a one-pot synthesis of cyanopyridone compound 201 using 4-hydroxy benzaldehyde (200) and 4-hydroxy acetophenone (199) with ethylcyanoacetate and excess ammonium acetate in 1,4-dioxane solvent. Followed by the preparation of the tri-ester functionalized compound 202 using synthesized compound 201 and ethyl chloroacetate in anhydrous K_2_CO_3_ using DMF as a solvent, the formed product was then converted into trihydrazide compound 203 by refluxing with hydrazine hydrate in ethanol solvent. On the other hand, alkylation of 3,4-hydroxy benzaldehyde (197) with various alkyl bromides afforded the 3,4-dialkoxybenzaldehydes 198a–e. Finally, the target products 204a–e were obtained by condensing the prepared 203 and 198a–e in the presence of glacial acetic acid as a catalyst (Scheme 26). The synthesized compounds formed columnar assemblies due to the presence of intermolecular hydrogen bonds and π–π (core to core) interactions. These compounds exhibited hexagonal columnar mesophase from ambient temperature to 110 °C. The optical studies revealed that all the synthesized compounds exhibited strong blue fluorescence emissions with maxima at around 389 nm, and it was also observed that the photophysical properties of the target compounds were not greatly affected by the terminal alkoxy chain length. An interesting fact is that these target compounds are emitters of blue fluorescence in both the liquid crystalline state and solution state; hence, these compounds are potential candidates for OLED applications.

**Scheme 27:**
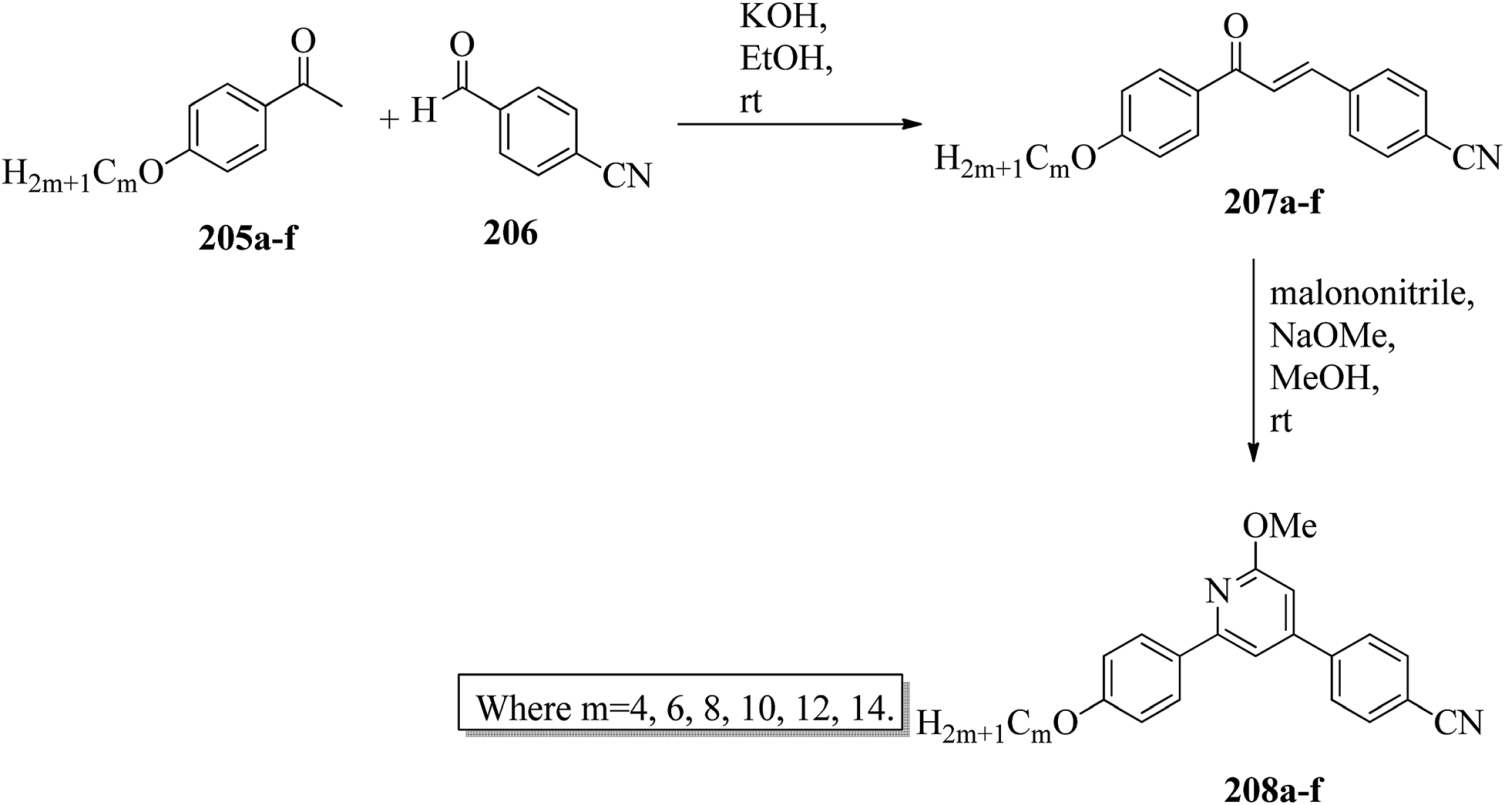
Synthesis of methoxypyridines 208a–f.

Further, Ahipa *et al.*^87^ designed and synthesized compounds with a bent core structure with three rings, *i.e.* 4-(2-(4-alkoxyphenyl)-6-methoxypyridin-4-yl)benzonitriles (208a–f), with variable alkoxy chain lengths. The synthetic route for the synthesis of 208a–f is shown in Scheme 27. The first step involves alkylation of 4-hydroxy acetophenone using alkyl bromides [*m* = 4 to 14 (only even)]. The second step involves the preparation of the required chalcones (207a–f) *via* Claisen–Schmidt reactions between the alkylated compounds (205a–f) and 4-cyano benzaldehyde (206); finally, the target methoxypyridine derivatives (208a–f) were obtained by cyclising the chalcones and malononitrile at room temperature in the presence of sodium methoxide. During cyclization, the cyano group (electron withdrawing group) attached to the phenyl ring does not facilitate the dehydrogenation process; rather, it undergoes dehydrocyanation. Because of this, the obtained products were methoxy pyridine derivatives, not 4-(4-cyanophenyl)-2-methoxy-6-(4-(alkoxy)phenyl)nicotinonitriles (Scheme 27). Further, POM, DSC and powder XRD studies of these synthesized compounds revealed that the compound with the shortest chain length, 208a [*i.e. m* = 4], exhibited nematic phase, while all the other derivatives exhibited orthorhombic columnar phase. Furthermore, the bent-shaped molecular structure of the target compound was readily confirmed by single crystal X-ray analysis. From the optical studies, it was observed that these compounds are blue emitting materials showing absorption and emission bands in the ranges of 335 to 345 nm and 415 to 460 nm, respectively. From the electrochemical studies, a band gap of 1.89 eV and HOMO and LUMO energy levels of −5.06 and −3.17 eV, respectively, were observed for the compound 4-(2-(4-octyloxyphenyl)-6-methoxypyridin-4-yl)benzonitrile (208c).

**Scheme 28:**
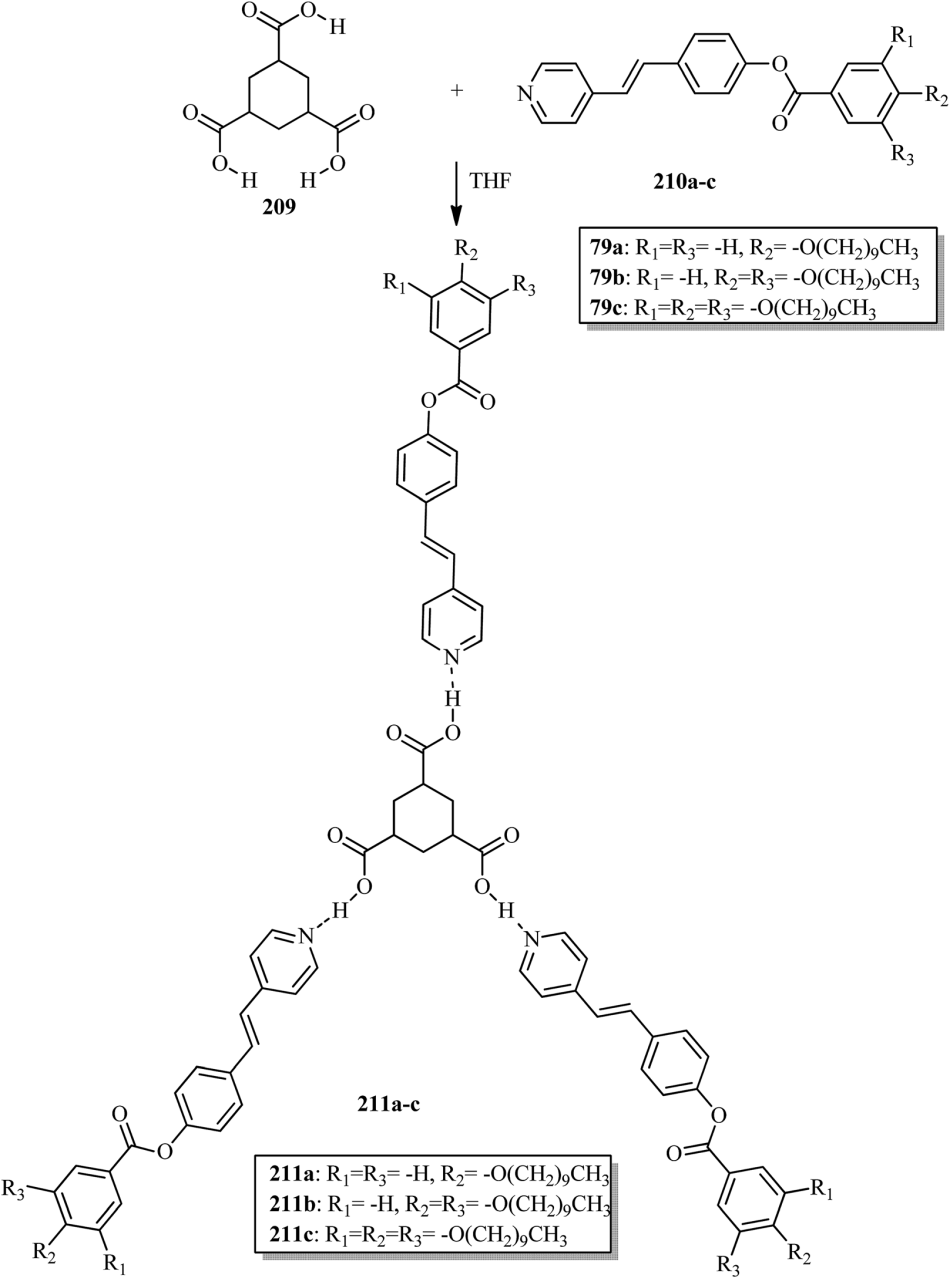
Synthesis of hydrogen bonded complexes 211a–c.

Lee *et al.*^88^ prepared discotic liquid crystals (211a–c) by the formation of hydrogen bonding between the discotic core 1,3,5-cyclohexanetricarboxylic acid (209) and the peripheral units of stilbazole derivatives (210a–c). The three stilbazole derivatives, *i.e.* 4-(4-decyloxybenzoyloxy)-4′-stilbazole (210a), 4-(3,4-didecyloxybenzoyloxy)-4′-stilbazole (210b), and 4-(3,4,5-tridecyloxybenzoyloxy)-4′-stilbazole (210c), were synthesized by referring to the report by Lee *et al.*;^89^ then, the hydrogen-bonded discotic complexes were prepared by mixing the stilbazole derivatives with 209 in THF solvent, and the solvent was removed slowly under reduced pressure (Scheme 28). Molecule 209 was non-mesogenic; among the three stilbazole derivatives, 210a exhibited smectic phase, while the others were non-mesogens. The phase transitions of the synthesized discotic complexes (211a–c) were found to be different from those of 210a–c. High enthalpy changes and monotropic phases were observed in complexes 211a and 211b; however, in the case of 211c, enantiotropic phase was observed. A small enthalpy change was observed in the case of complex 211c because the number of flexible alkyl chains decreased the π–π interactions of stilbazole arms in the discotic mesogen. Complex 211a exhibited rectangular columnar phase, whereas complexes 211b and 211c exhibited hexagonal columnar phases. Along the column axes of these complexes, dipole–dipole interactions occur between the carbonyl groups; hence, they exhibited rectangular columnar and hexagonal columnar mesophases. These derivatives may be useful in electronic applications.

**Scheme 29:**
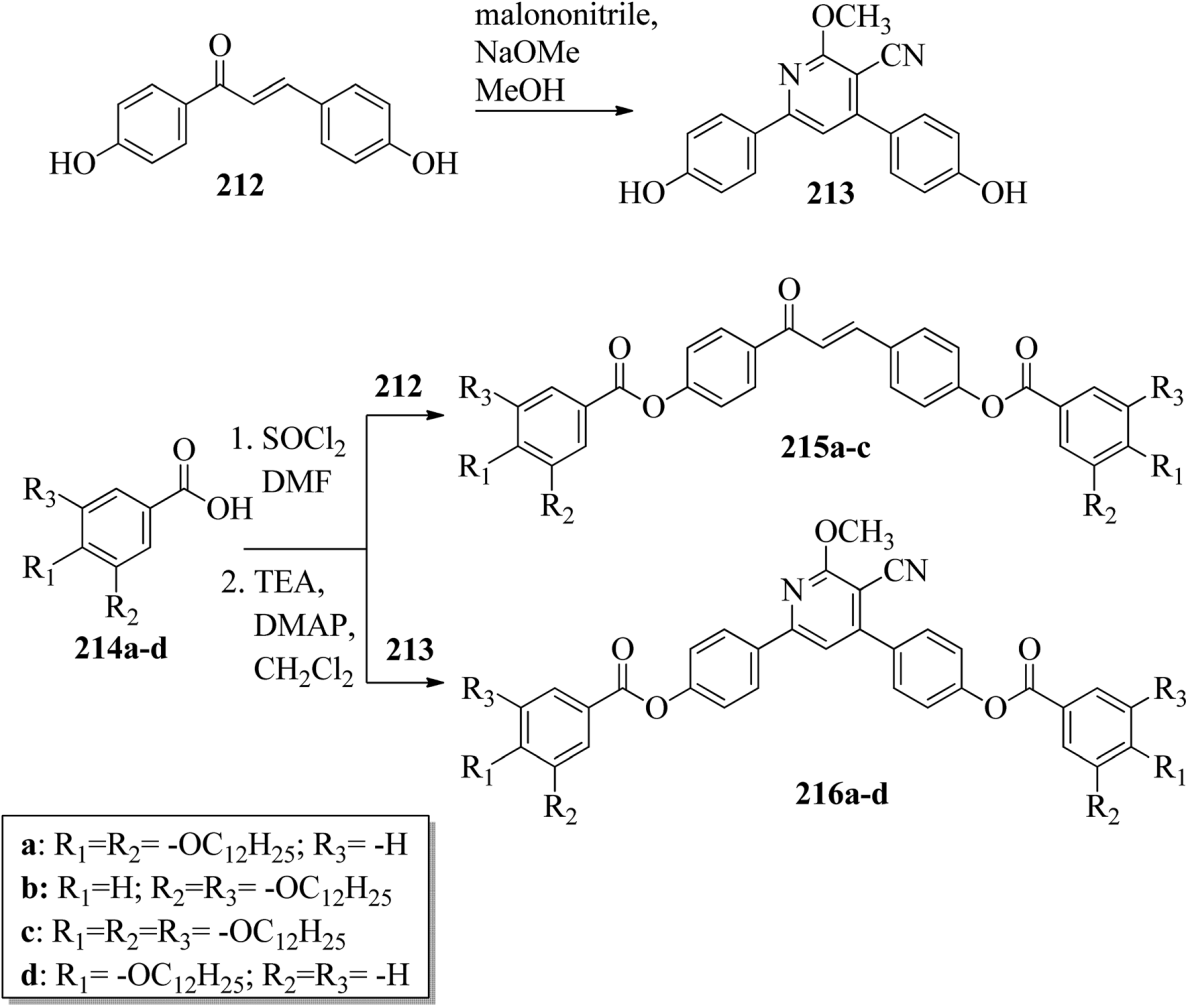
Synthetic route for compounds 215a–c and 216a–d.

Coelho *et al.*^90^ synthesized seven new bent-shaped and polycatenar bent-shaped compounds which are derivatives of chalcone and cyanopyridine. Using POM, DSC, and XRD, the mesomorphic behavior of the compounds was investigated and correlated with their molecular structures. First, substituted benzoic acids were synthesized by referring to the previous report by Dal Bó *et al.*^91^ Further, the acid group was protected by Fischer esterification, followed by alkylation of the hydroxyl group (Williamson etherification) using 1-bromododecane, butanone and K_2_CO_3_. Also, TBAB (tetrabutylammonium bromide) was used to ensure complete alkylation in cases of alkylation of more than one hydroxyl group. Finally, the ester-to-acid conversion was performed through base hydrolysis. At the same time, chalcone 212 was prepared through a Claisen–Schmidt condensation reaction of 4-hydroxybenzaldehyde and 4-hydroxyacetophenone in the presence of boron trifluoride diethyl etherate in dioxane.^92^ Further, to obtain the cyanopyridine centre (213), chalcone was cyclised using sodium methoxide and malononitrile in methanol.^93^ Finally, the substituted carboxylic acids were converted to their respective benzoyl chlorides using SOCl_2_; then, they were esterified with the dihydroxyl bent core 212 or 213 (Scheme 29). Only two bent-core hexacatenar molecules (215c and 216c) exhibited liquid crystal properties at room temperature, and all the other molecules (215a–b and 216a–b,d) were non-mesogens. POM studies revealed that compound 215c exhibited monotropic liquid crystalline behavior, *i.e.* it exhibited a fan-shaped focal conic texture (hexagonal columnar) on cooling, whereas 216c exhibited enantiotropic liquid crystalline behavior, *i.e.* it exhibited the fan-shaped focal conic texture (hexagonal columnar) on cooling as well as on heating. Also, it was revealed that as the number of the alkoxy chains increased, the melting points of compounds 215a–b, 216a–b, and 216d and the clearing temperatures of 215c and 216c decreased. This is because the molecular packing was hindered by the additional alkoxy chains. Further, it was noted that chalcone core derivatives 215a–c had higher transition temperatures than cyanopyridine core derivatives 216a–d, even though 216a–d contain one extra ring in their structures, because of the decrease in the co-planarity between the benzene ring and the pyridine heterocycle due to steric effects. The cyanopyridine derivatives showed average luminescence quantum yields (*i.e.* ranging between 18% and 27%) with an emission maximum at 371 nm. Fluorescence was induced in the target molecules because of the inclusion of the cyanopyridine core between π-conjugated structures. This study also indicated that the length and position of the alkyl chains does not influence the luminescence properties of these compounds.

**Scheme 30:**
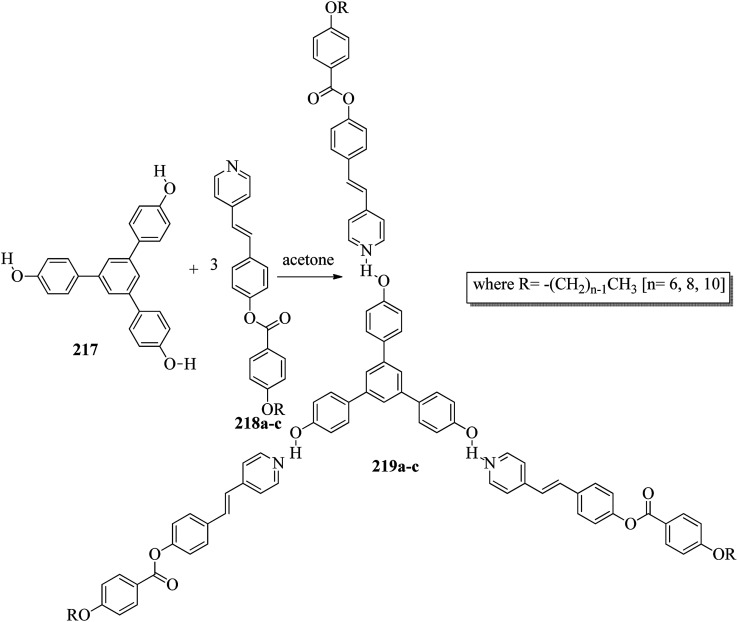
Preparation of supramolecular discotic liquid crystals (219a–c).

Lee^94^ prepared hydrogen bonded supramolecular discotic liquid crystals using mesogenic stilbazole derivatives (218a–c) and non-mesogenic 1,3,5-*tris*(4-hydroxyphenyl)benzene (217). The three stilbazole derivatives (218a–c) were synthesized according to previous literature, *i.e.* Lee *et al.*^89^ The discotic complexes were prepared by dissolving stilbazole derivatives 218a–c and 217 in anhydrous acetone and sonicating for 1 h; then, the solvents were evaporated under reduced pressure (Scheme 30). The prepared discotic complexes (219a–c) exhibited monotropic mesophases by quenching the enantiotropic smectic mesophase behavior of the stilbazole derivatives; also, the prepared complexes showed lower mesophase transition temperatures than the stilbazole derivatives. Nematic columnar mesophases were observed in complexes 219a and 218b, whereas discotic nematic mesophase was observed in complex 219c. This is because of the long alkyl chains attached to the central core, which decrease the packing efficiency of the asymmetric discotic core.

**Fig. 13:**
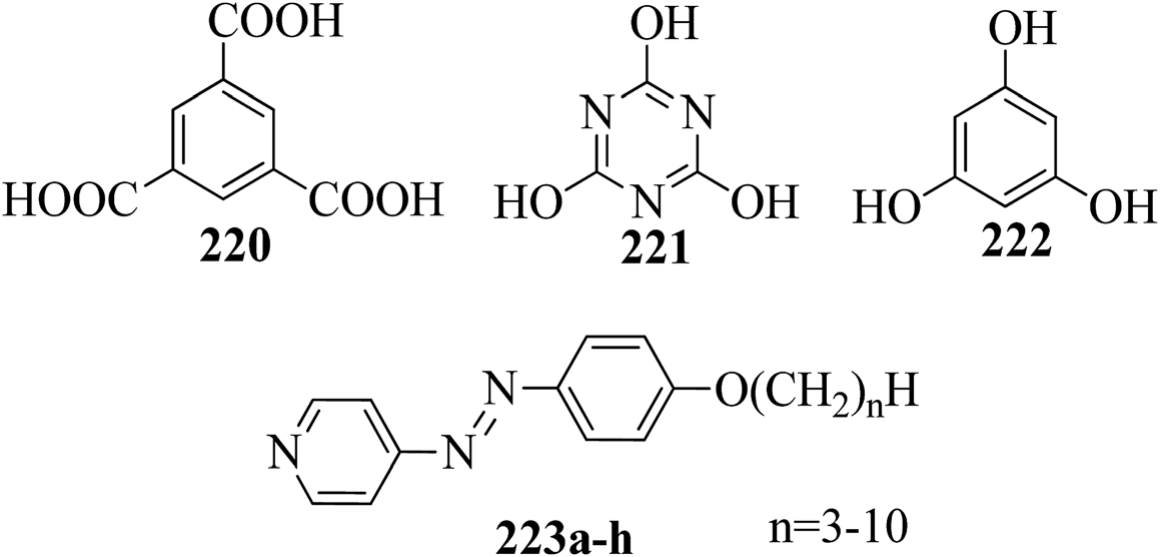
Structures of proton donors 220, 221, and 222 and the proton acceptors 223a–h.

Pfletscher *et al.*^95^ prepared a series of C3-symmetric assemblies by combining simple non-mesogenic azopyridines and commercially available core units (cyanuric acid (221), trimesic acid (220) and phloroglucinol (222)) and studied their liquid crystalline properties as well as their fast and reversible responses to irradiation with light. The commercially available core units and azopyridine derivatives (223a–h) were dissolved in acetone solvent, followed by heating in a microwave oven; then, slow evaporation of the solvents afforded the final expected complexes. The structures of proton donors 220, 221, and 222 and proton acceptors 223a–h are presented in Fig. 13. All the individual starting materials were non-mesogenic in nature. However, selected core units displayed C3-symmetry and common potential to form clusters with the azopyridine moieties; great variations were noted in the mesomorphic behavior of the assemblies. The authors expected weaker interactions in the hydrogen bonded phloroglucinol complexes and in the hydrogen bonded trimesic and cyanuric acid complexes. However, they observed inhomogeneous melting in the hydrogen bonded trimesic and cyanuric acid complexes at the time of the POM study. In contrast, inhomogeneous melting formed isotropic phase in the hydrogen bonded phloroglucinol complexes. This finding was ascribed to the disaggregation of the hydrogen bonded complexes of cyanuric and trimesic acid clusters with azopyridine in favour of powerful hydrogen bonding within the phase-partitioned homo-crystals of trimesic and cyanuric acid. This is in line with the notably higher melting points of cyanuric acid (320 °C) and trimesic acid (380 °C) with respect to phloroglucinol (215 °C). During the cooling cycle of the phloroglucinol series, they observed Schlieren textures (characteristic of nematic mesophase). Hence, they focused only on the phloroglucinol series. In the phloroglucinol series, 222/223a was found to be non-mesogenic in nature, *i.e.* it melted isotropically at 142 °C. Upon comparing the thermal properties, they observed that increasing the alkyl chain length at the peripheral unit stabilizes the mesophases. Δ*T* varied from 5.6 °C for 222/223b to 26.7 °C for 222/223g. In addition, the samples were illuminated with a laser pointer (405 nm, 5 mW) to study the photo-responsive properties of the hydrogen bonded liquid crystals. The same effect was observed for all the complexes. Originally, the complexes were in the *trans* state, which shows the Schlieren texture of nematic phase. However, the liquid crystal texture disappeared on illumination at 405 nm, which provides evidence for photo-induced phase transition from mesophase to isotropic phase. The phase shift was ascribed to the photo-isomerisation of the azopyridine units from the *trans* to *cis* configuration because the bent shape of the *cis* isomers leads to mesophase destabilization. Mesophase was observed within a second by cutting off the irradiation. The sample was illuminated with a red (650 nm, 5 mW) and a green (532 nm, 5 W) laser pointer to repeat the *cis*/*trans* isomerization of the azopyridine and thereby cause the photo-induced phase transition. No effect on the mesophases was noted. Hence, they proposed that the formerly noted phase transition was due to azopyridine moiety isomerisation and not to local thermal excitation. Due to these properties of the complexes, they are attractive for opto-electronic applications.

**Fig. 14:**
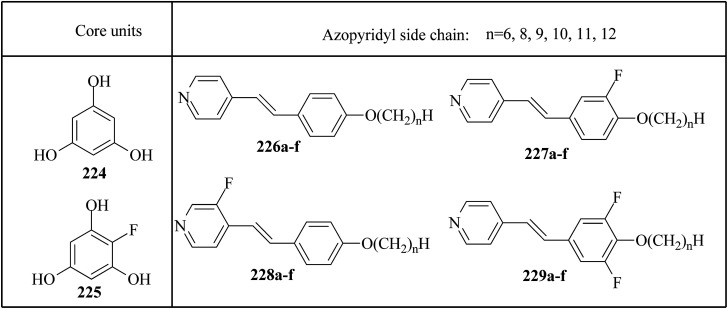
Structures of the core units and azopyridyl side chains.

Spengler *et al.*^96^ prepared hydrogen bonded complexes using phloroglucinol (224) and its mono-fluorinated derivative (225) as the hydrogen bond donors and a series of azopyridyl side chains with different fluorination patterns as the hydrogen bond acceptors. The core unit and azopyridyl (Ap) side chain were taken in 1 : 3 molar ratios and separately dissolved in acetone solvent followed by mixing of the solutions. After solvent evaporation, the clusters were dried under vacuum. Fig. 14 presents the structures of the core units and azopyridyl side chains. None of the individual starting materials exhibited liquid crystal properties. In contrast, most of the prepared hydrogen bonded complexes exhibited mesogenic properties on cooling. In addition, they used the 224/226a–f series as a reference to compare their mesomorphic behavior with that of the fluorinated complexes. The complexes of 226a–f and 227a–f with 224 as well as with 225 exhibited the characteristic Schlieren appearance of a nematic mesophase; the complexes of 228a–f with 224 or 225 did not exhibit mesophase, *i.e.* they crystallized directly from isotropic liquid phase. Similar behavior was noted for the complexes of 229a–f with 225. However, the typical conic texture of smectic phase was observed in the complexes of 229a–f with 224. The mesophase range moderately broadened from Δ*T* = 8.9 °C for 224/226a to Δ*T* = 21.0 °C for 224/226c. Further increasing the alkyl chain length caused the mesophase ranges to decrease. A slight even–odd effect was observed on the transition temperatures. Similar morphologies were observed in the 225/226a–f aggregates when compared to the 224/226a–f aggregates. However, fluorination on the core unit affects the transition temperature. While *T*_c_ was unaffected, *T*_N-Cr_ was notably lowered, leading to moderate widening of the mesophase ranges. The widening of the mesophase was ascribed to the stabilization of the hydrogen bonded complexes by non-classical hydrogen bonds between the Ap side chains (C–F⋯H–C) and the core unit. 224/228a–f and 225/228a–f did not exhibit mesophases. However, shifting of their melting points towards lower temperatures was observed with respect to the non-fluorinated Ap analogues. This is because repulsive interactions between the electronic environment of the diazo bond and the fluoro substituent decrease the co-planarity of the Ap system and, as a result, the stability of the mesophase. 224/227a–f and 225/227a–f displayed liquid crystal behavior, in contrast to 224/228a–f and 237/240a–f. By increasing the alkyl chain length in the 224/227a–f complexes, the mesophase range was found to increase from Δ*T*_meso_ = 8.1 °C (*n* = 6) to Δ*T*_meso_ = 13.3 °C (*n* = 12). Up to a chain length of *n* = 9, the mesophase ranges of the 224/226a–f complexes increased and then gradually decreased again. *T*_c_ of the 224/227a–f complexes notably decreased compared to those of the 224/226a–f complexes, which is a well-known effect of lateral fluoro substitution. Different mesomorphic behavior was observed in the 224/229a–f and 225/226a–f complexes than in previously described systems, *i.e.* they exhibited characteristic textures of smectic phase, while nematic phases were observed in all other systems. Illumination by a laser pointer (405 nm, 5 mA) of the complexes resulted in photoinduced phase transition from mesophase to isotropic phase. In the complex 224/227b, N–I switching occurred instantaneously (1 to 2 s); after the laser was turned off, the complex required around ∼8 s to return to its original state. Longer times (∼3 to 5 s) were observed in 224/229b for the mesophase to isotropic phase transition and also for the reverse process (∼12 s). The remarkably longer response of the difluorinated compound can be ascribed to the high molecular order of the smectic phase.

**Scheme 31:**
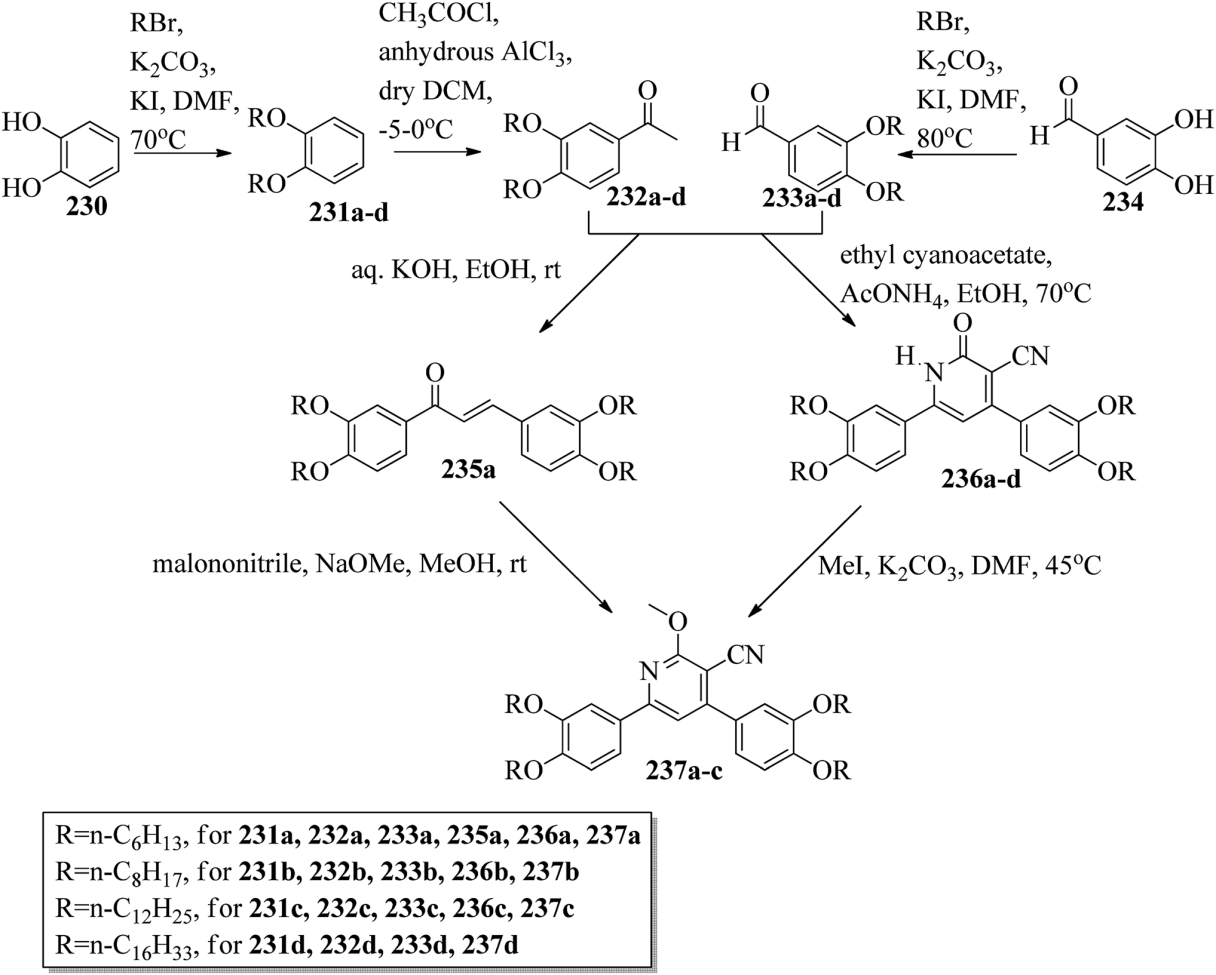
Synthesis of 237a–c.

Vinayakumar^97^ synthesized mesogenic bird-shaped cyanopyridone derivatives and studied their mesogenic properties. The synthetic route for the preparation of the cyanopyridone-based mesogens is illustrated in Scheme 31. Williamson etherification of catechol 230 followed by Friedel–Crafts acylation with acetyl chloride yielded 3,4-dialkoxyacetophenones as intermediates (232a–d). Alkylation of 3,4-dihydroxybenzaldehyde 234 using *N*-alkyl bromides yielded the other intermediates, 3,4-dialkoxybenzaldehydes 233a–d. Symmetrically substituted compounds 236a–d were synthesized by reacting intermediates 233a–d with precursors such as 232a–d in the presence of excess ammonium acetate and ethyl cyanoacetate. Using different synthetic protocols, they separately synthesized the 2-methoxy-3-cyanopyridine derivatives 237a–c. Here, compound 237a was prepared by Claisen–Schmidt condensation of ketone 232a with aldehyde 233a to yield the chalcone, followed by chalcone cyclization with malononitrile in the presence of sodium methoxide. In this method, they obtained poor yield of the product; hence, they used an alternate method to obtain 237a. In this method, using methyl iodide, they alkylated compound 236a directly in the presence of base to obtain compound 237a in good yield. Using a similar process, they prepared the other two compounds, 237b and 237c. The thermotropic mesophase action of 236a–d showed that increasing the peripheral chain length led to destabilization of their mesogenic properties. Columnar oblique phase was observed in 236a with four hexyloxy chains. In contrast, two distinct columnar hexagonal mesophases were observed as the chain length in 236b increased by two methylene groups, which may be due to the increase in the diameter of the overall disc. According to the order of molecules along the columnar axis, dodecyloxy-substituted compounds 236b and 236c displayed intra-columnar transitions from ordered hexagonal columnar phases to disordered hexagonal columnar phases. Hence, in this type of mesogen, the symmetry of the columnar phases is decided by the chain length. Changes in enthalpy of about 4.06 kJ mol^−1^ and 2.49 kJ mol^−1^ were observed for the transition from ordered to disordered columnar phases in the cases of 236b and 236c, respectively. For the weaker columnar transitions, they noted higher values of enthalpy, which was ascribed to dipolar interactions as well as the strong intermolecular hydrogen bonding possessed by the mesocompounds in oblique hexagonal columnar phase. Thus, significant energy is required to disturb the order of the molecules. Hence, this intra-columnar transition was assigned as the first order transition. The XRD results revealed that the columnar mesophases in compounds 236a–d comprise two molecules per slice of column. Therefore, they presumed that the central cyanopyridone core participates in dimer formation through intermolecular hydrogen bonding to afford the disc-shaped structures. They synthesized another series of molecules in order to prove this assumption, 237a–c, by replacing the central core cyanopyridone with 2-methoxy-3-cyanopyridine, which lacks hydrogen bonding sites. However, the compounds failed to exhibit any mesomorphic character, which supports that the part of the central core forms the dimer arrangement. Finally, they concluded that mesophase formation in the bird-shaped cyanopyridones was decided by hydrogen bonding interactions.

Recently, Wang *et al.*^98^ synthesized a series of star-shaped mesogens comprising a [1,1′-biphenyl]-4,4′-diyl diisonicotinate moiety carrying different counter ions, such as Br^−^, dodecylbenzenesulphonate anion, (+)-10-camphorsulfonate anion, H_2_PO_4_^−^ and BF_4_^−^. Further, these compounds exhibited nematic phase, and it was noted that the mesophase temperature range increased with increasing alkyl chain length and that the clearing point decreased with increasing anion size. These star-shaped liquid crystals displayed good ionic conductivity and properties, which was further attributed to electrostatic attraction of ions, electron delocalization of the π–π stacking conjugation effect and the long and ordered molecular structure.

### 2.3. Pyridine-based bent-shaped mesogens

In bent-shaped mesogens, the two mesogenic groups are connected through a bent-shaped rigid core, *e.g.*, resorcinol or isophthalic acid. In this section, the synthetic routes available for the preparation of different pyridine-based bent-shaped mesogens and their properties are summarized.

**Scheme 32:**
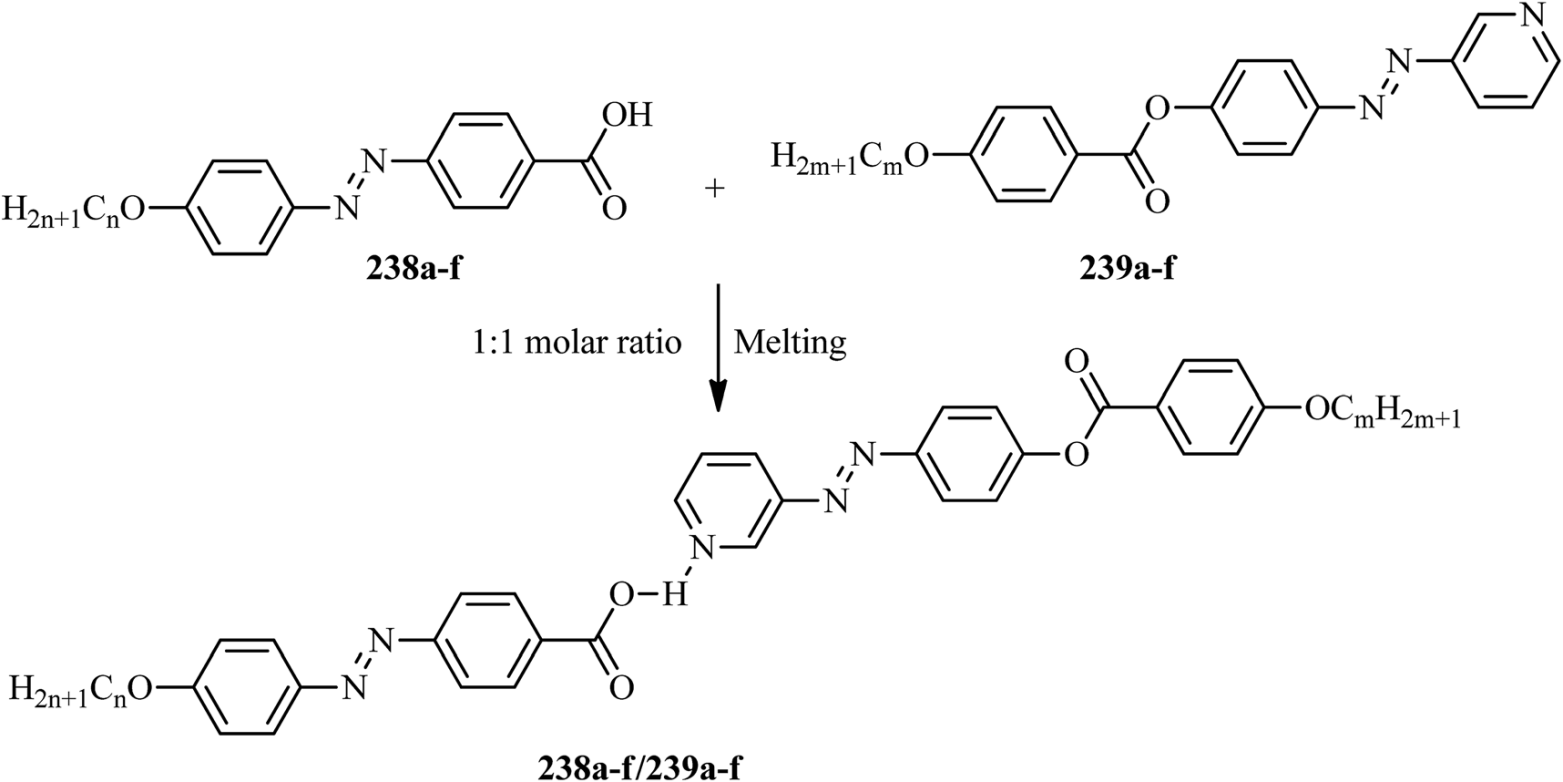
Preparation of supramolecular complexes 238a–f/239a–f.

Ahmed and Naoum^99^ prepared new hydrogen bonded supramolecular complexes using 1 : 1 molar ratios of 4-(3′-pyridylazo)-4′′-alkoxybenzoates (239a–f) and 4-alkoxyphenylazo benzoic acids (238a–f) with terminal alkoxy chain lengths varying from 6 to 16 carbons; they studied their mesophase behavior by DSC and polarizing light microscopy (PLM). Pyridine-based derivatives 239a–f and 4-*n*-alkoxyphenylazo benzoic acids 238a–f were prepared as described in previous work by Naoum *et al.*,^100^ Janietz *et al.*,^42^ and Lizu *et al.*^101^ The supramolecular complexes 238a–f/239a–f were prepared by melting of 238a–f and 239a–f followed by stirring and drying (Scheme 32). All the complexes exhibited smectic C and nematic mesophases. The formation of hydrogen bonds was confirmed by FT-IR and UV-visible absorption spectroscopy. This study revealed that increasing the length of the alkoxy chain decreases the nematic transition enhancement (Δ*T*) in the case of the base component; on the other hand, Δ*T* increases with increasing chain length in the acid component, so the nematic phase stability is dependent on the alkoxy chain length of the acid component. The entropy associated with the smectic transition (Δ*S*_C–N_) is greater than that associated with the nematic transition (Δ*S*_N–I_). The entropies of both types vary unevenly with both alkoxy chain lengths *m* and *n*. Due to the nonlinear shape of the supramolecular complexes, a low entropy change occurs. The stability of the mesophases (TC) of the prepared complexes (238a–f/239a–f) was found to be higher than that of their corresponding angular complexes prepared by Naoum *et al.*;^100^ however, smectic A phase was quenched and smectic C and nematic phases were formed for all complexes.

**Scheme 33:**
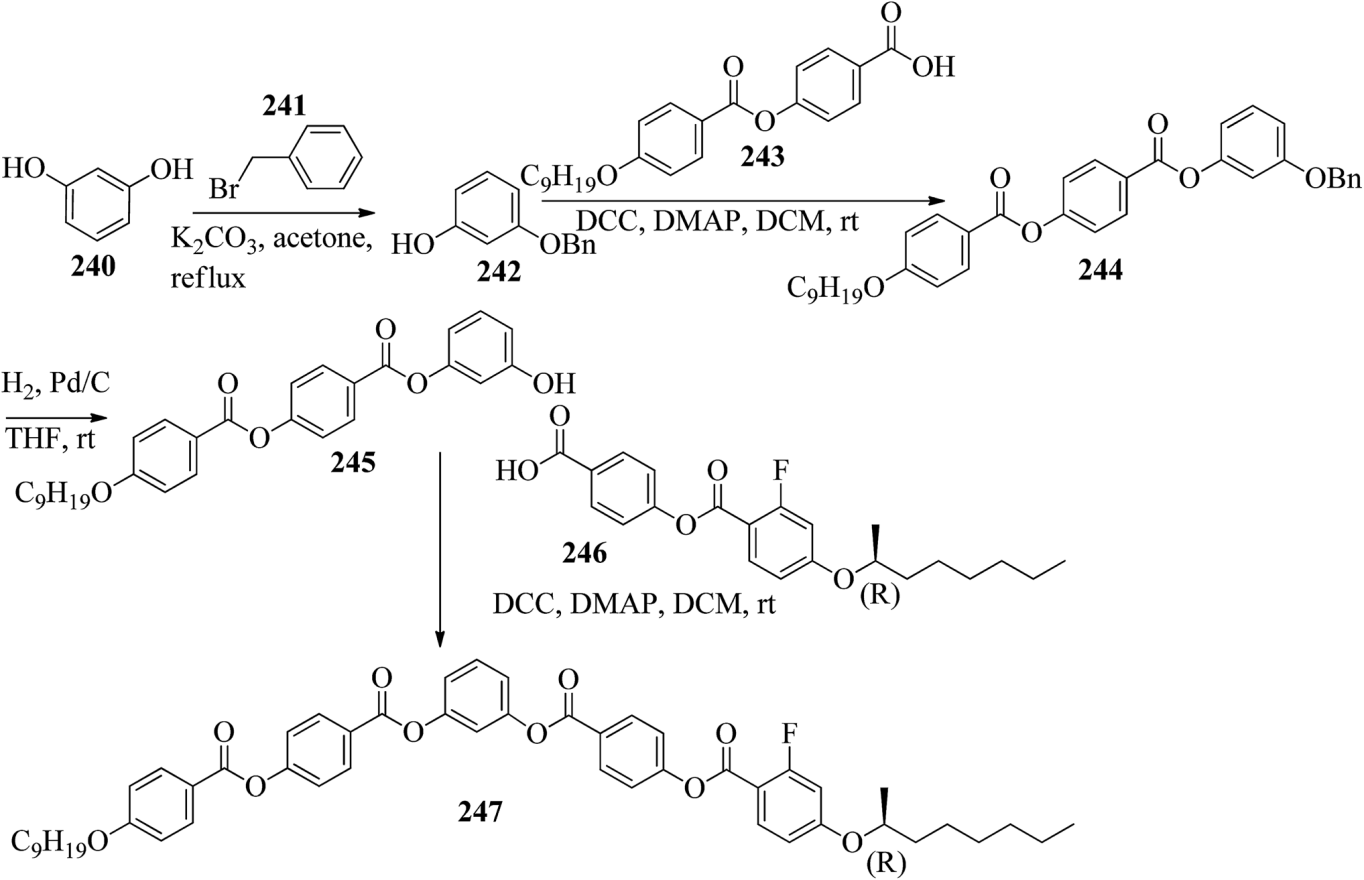
Synthetic route for the covalent bent core 247.

**Scheme 34:**
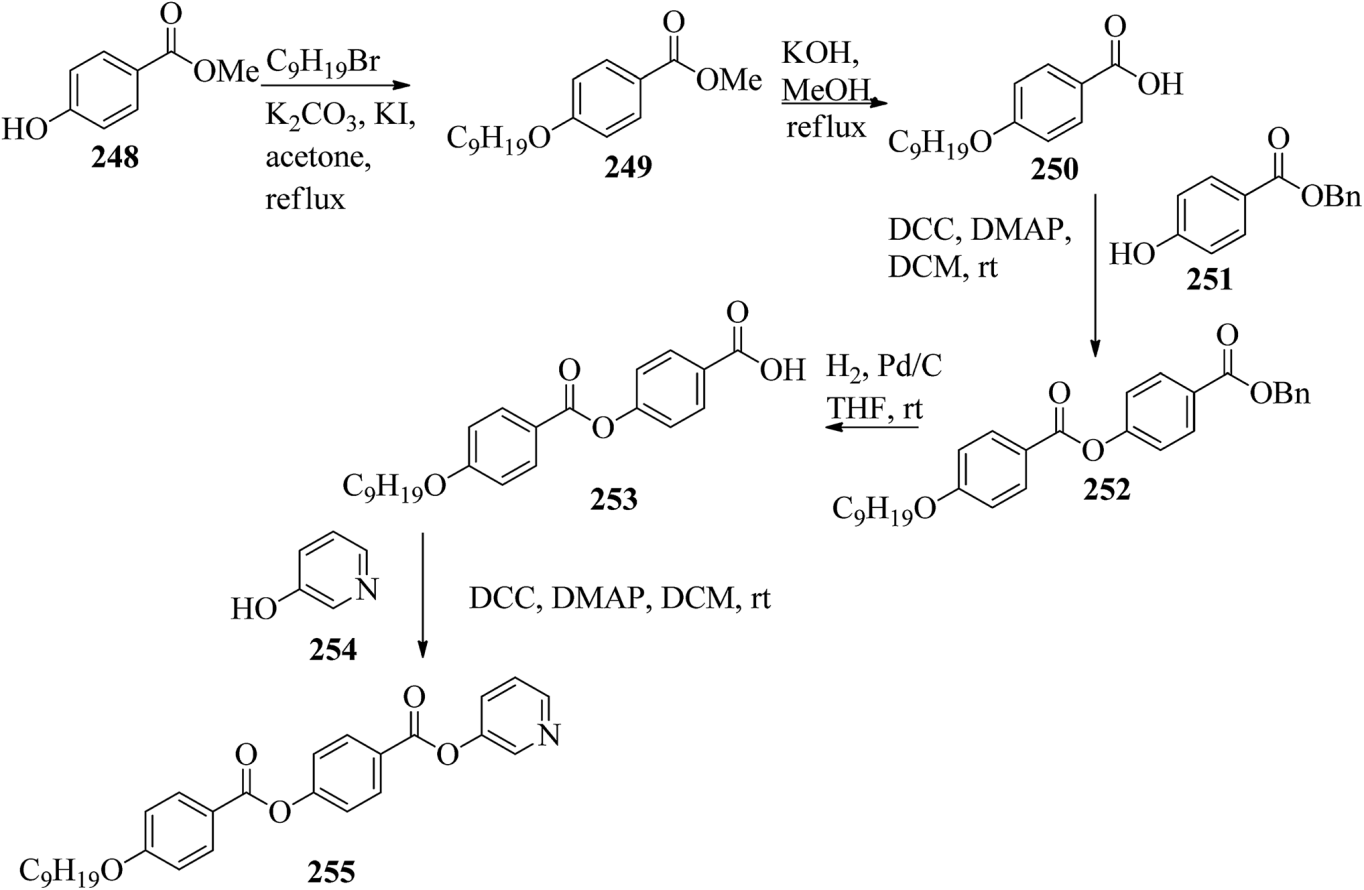
Synthetic route for the proton acceptor 255.

**Scheme 35:**
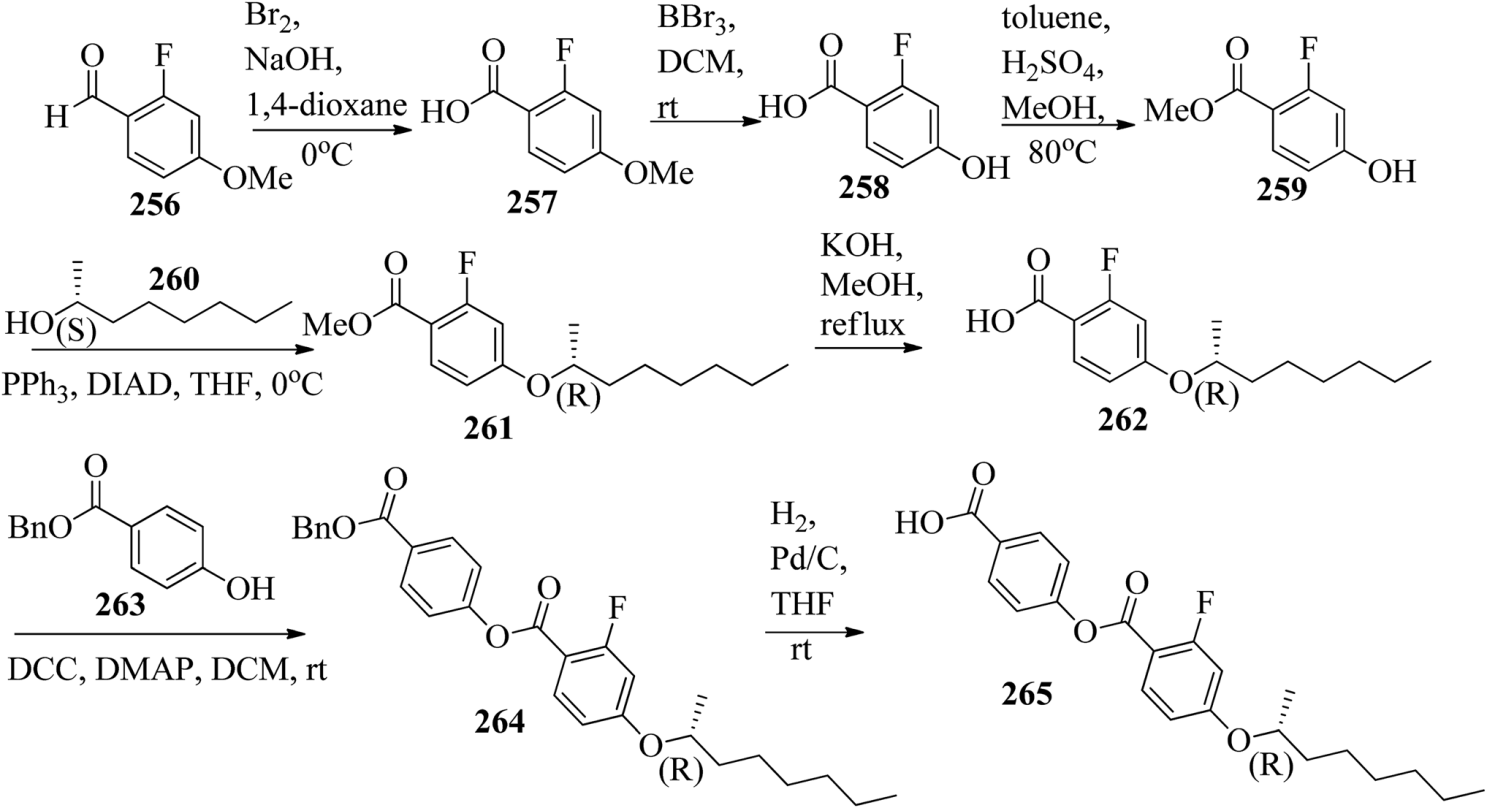
Synthetic route for the proton donor 265.

**Fig. 15:**
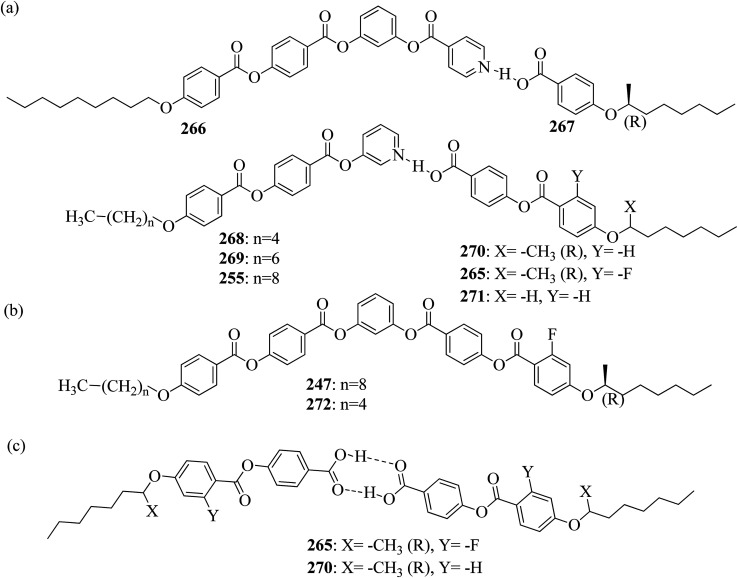
(a) Structures of the hydrogen bonded complexes; (b) structure of the covalent bonded analogue; (c) structures of the hydrogen bonded dimers.

Han *et al.*^102^ prepared hydrogen bonded liquid crystal complexes with bent cores and compared them with their covalent analogues. Compounds 245 and 246 along with DCC and DMAP reagents were dissolved initially in dry DCM and maintained under nitrogen at room temperature for 16 h. After completion of the reaction, the product was extracted using a water/DCM solvent mixture and the organic layer was dried using anhydrous magnesium sulphate. Further, the slow evaporation of solvent under reduced pressure afforded 247 as the product (Scheme 33). In a similar way, hydrogen acceptor 255 was synthesized using compound 253 and 3-hydroxypyridine (254) instead of compounds 245 and 246 (Scheme 34). Hydrogen donor 265 was synthesized by stirring compound 264 and 15% Pd/C as a catalyst at room temperature in THF under hydrogen overnight. The catalysts were removed through Celite filtration, and the solvent was evaporated under reduced pressure to afford the product (Scheme 35). Further, appropriate molar ratios of hydrogen acceptors and hydrogen donors were dissolved in THF, and evaporating the solvents yielded the final hydrogen bonded complexes. The structures of the hydrogen bonded complexes, covalent bonded analogues, and hydrogen bonded dimers are represented in Fig. 15. Complexes 266/267 did not exhibit mesogenic properties, whereas complexes 255/265 and 255/270 showed nematic phases; also, their covalent analogue 247 was found to be non-mesogenic. This is because it has a more rigid bent core than the others. The hydrogen bonded diad 255/270 showed a higher phase transition temperature and narrower chiral nematic phase range than 255/265 with lateral fluoro-substitution. They also studied the effects of changing the molar ratio of the proton donor in the complexes. Complexes 255/265, 268/265, and 269/265 with a 1 : 1 molar ratio did not exhibit blue phases; however, blue phases started to form with increasing molar ratio of 265. Above 55 mol%, blue phase started to appear, but after 80 mol%, the blue phase disappeared; this is due to biaxial dilution of the over-supplied uniaxial rod-like dimer 265. Furthermore, they mixed the covalent analogue 247 with different molar ratios of 265 and checked for blue phases. Blue phase was induced at 75.0 to 82.4 mol% of 265. They also observed that increasing the alkyl chain length widens the blue phase range, *i.e.*268/265 < 269/265 < 255/265. Due to the narrow ranges of blue phase in the covalent-bonded bent-core mixtures, they concluded that introduction of hydrogen bonds in the bent-core effectively stabilizes the blue phase. The widest blue phase range of Δ*T* = 12.0 °C was observed in the 255/265 (3/7 mol mol^−1^) complex. To compare the effects of fluoro groups and the chiral centres of the H donors on the hydrogen bonded complexes, they prepared two more hybrid hydrogen bonded complexes with dual hydrogen donors, *i.e.* complex 255/(265 + 271) and 255/(265 + 270). As the 271 mol% in 255/(265 + 271) complex increased, the blue phase range decreased and vanished above 70 mol% of 271. In 255/(265 + 270), as the 270 content increased, the blue phase range decreased faster than in the previous case; it vanished above 30 mol% of 270 in the complex.

**Scheme 36:**
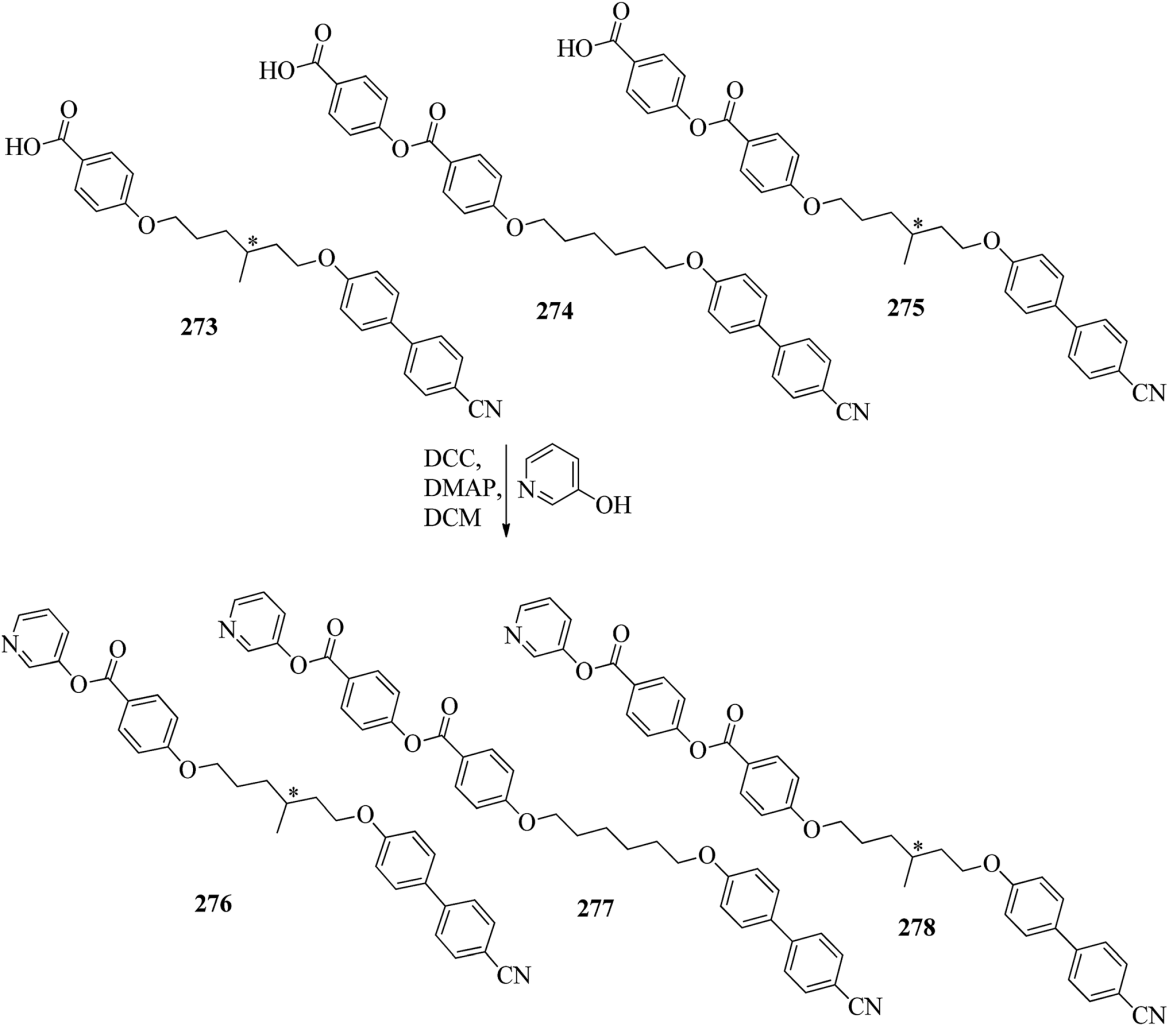
Synthesis of hydrogen acceptors 276–278.

**Scheme 37:**
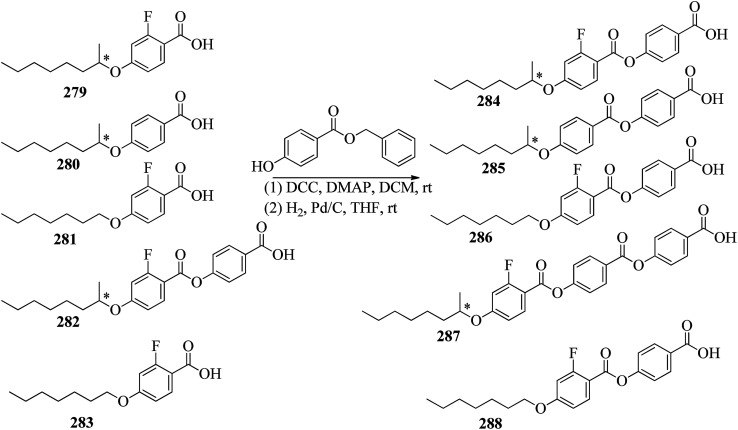
Synthesis of hydrogen donors 284–288.

**Fig. 16:**
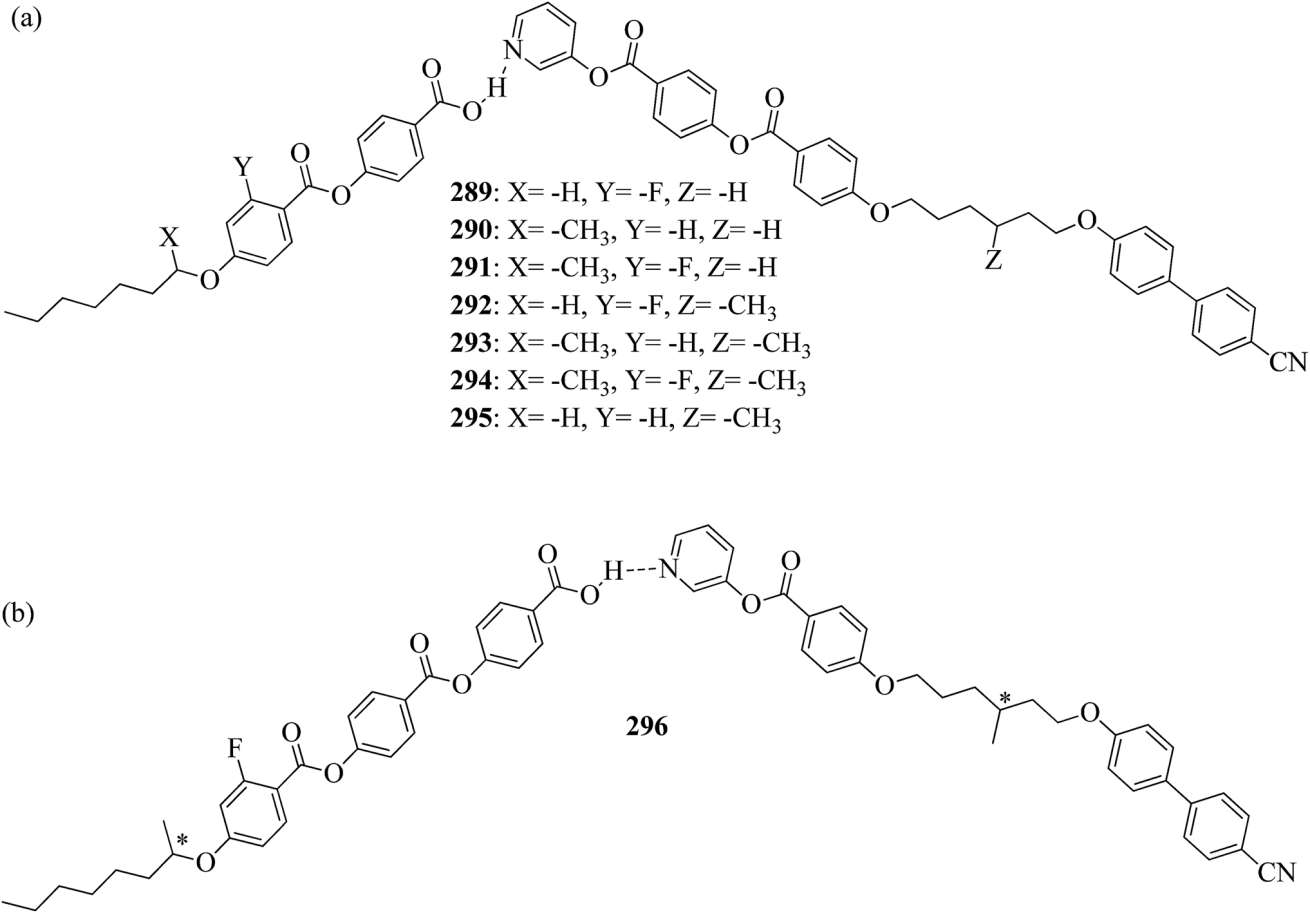
(a) Structures of hydrogen bonded complexes 289–295; (b) structure of hydrogen bonded complex 296.

Han *et al.*^103^ synthesized a hydrogen bond acceptor and donors, prepared their complexes and studied their mesogenic properties. Compound 273 and pyridin-3-ol were dissolved in DCM, and DMAP was also added and dissolved under nitrogen; then, DCC was added to this solution, and the reaction was maintained for 16 h at room temperature. The reaction mixture was filtered and washed with DCM solvent, followed by extraction with deionized water/DCM; then, the organic phase was dried using anhydrous MgSO_4_ and concentrated using a rotary evaporator. Finally, the obtained product was purified by silica gel column chromatography to afford the pure hydrogen bond acceptor (276). Similarly, using compounds 274 and 275, the other hydrogen bond acceptors (277 and 278) were synthesized (Scheme 36). Compound 279 and benzyl 4-hydroxybenzoate were dissolved in DCM, and DMAP was also added and dissolved under nitrogen; further, DCC was added to the solution, and the reaction mixture was maintained for another 16 h at room temperature. The reaction mixture was filtered and washed with DCM solvent, followed by extraction with deionized water/DCM; then, the organic phase was dried using anhydrous MgSO_4_ and concentrated using a rotary evaporator. By silica gel column chromatography, the intermediate was collected. In THF, the intermediate and 15% Pd/C were dissolved, and this reaction was maintained at room temperature overnight under hydrogen. After completion of the reaction, the mixture was filtered and washed with THF solvent. This was followed by solvent evaporation and recrystallizing in an *n*-hexane/DCM solvent mixture, which resulted in pure hydrogen donor 284. Similarly, other hydrogen donors (285–288) were obtained using compounds 280–283 (Scheme 37). Finally, the hydrogen bonded complexes were prepared by dissolving appropriate amounts of proton acceptor and donor compounds in chloroform/THF (∼3 : 1 vol) and evaporating the solvent slowly. The structures of hydrogen bonded complexes 289–295 and the structure of hydrogen bonded complex 296 are presented in Fig. 16. Only complex 293 exhibited a wide range of blue phases in addition to smectic A and cholesteric phases; all the other complexes exhibited smectic A and cholesteric phases only. Due to the more symmetric hydrogen bonded bent-core structure of 294 and its large helical twisting power (HTP), this complex exhibited lower phase transition temperatures and a wider cholesteric phase range than the supramolecular diad 295. Further, studying the effects of various numbers of the chiral centers on the mesogenic properties of complexes 292, 294 and 291 revealed that a wider cholestric phase 
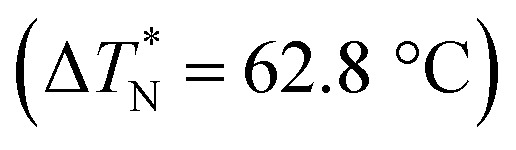
 was observed for 294 than for the other two complexes. Therefore, they concluded that by introducing double chiral centers on both the proton acceptor and donor, the cholestric phase was stabilized. On the other hand, a narrower smectic A phase range of Δ*T*_SmA_ = 26.2 °C was observed for complex 294 than for the other two complexes on cooling. Studies of the effects of the location of the chiral center on the mesogenic properties of complexes 292 and 291 revealed that the chiral center on side chain 291 has a narrower cholestric phase and smectic A phase than 292, where chirality was present on the flexible spacer. They also compared supramolecular diads with lateral fluoride substitution (*i.e.*, 291, 292, and 294) with those without lateral fluoride substitution (*i.e.*, 290, 293, 295) to study the effects of fluoride on the supramolecular diads. In the double chiral centred complexes, blue phase was observed in 293 but was not observed in 294. Smaller dipole moments and lower HTP values induced lower phase transition temperatures in all supramolecular diads without lateral fluoride substitution compared with the lateral fluoride substituted complexes. Due to the stronger helical twisting power HTP = 4.2 μm^−1^ of 293, blue phase was observed. They also studied the effects of the molar ratio of the proton acceptor/proton donor on the blue phase of supramolecular diad 293; it was revealed that the widest blue phase range was observed at 50 mol% of the hydrogen donor in 293 (*i.e.*, proton acceptor/proton donor = 1 : 1). This is due to the presence of rod-like and hydrogen bonded bent-core mesogens rather than when the hydrogen donor = 75 mole% where the mesogen possesses a linear hydrogen bonded rod-like structure.

**Scheme 38:**
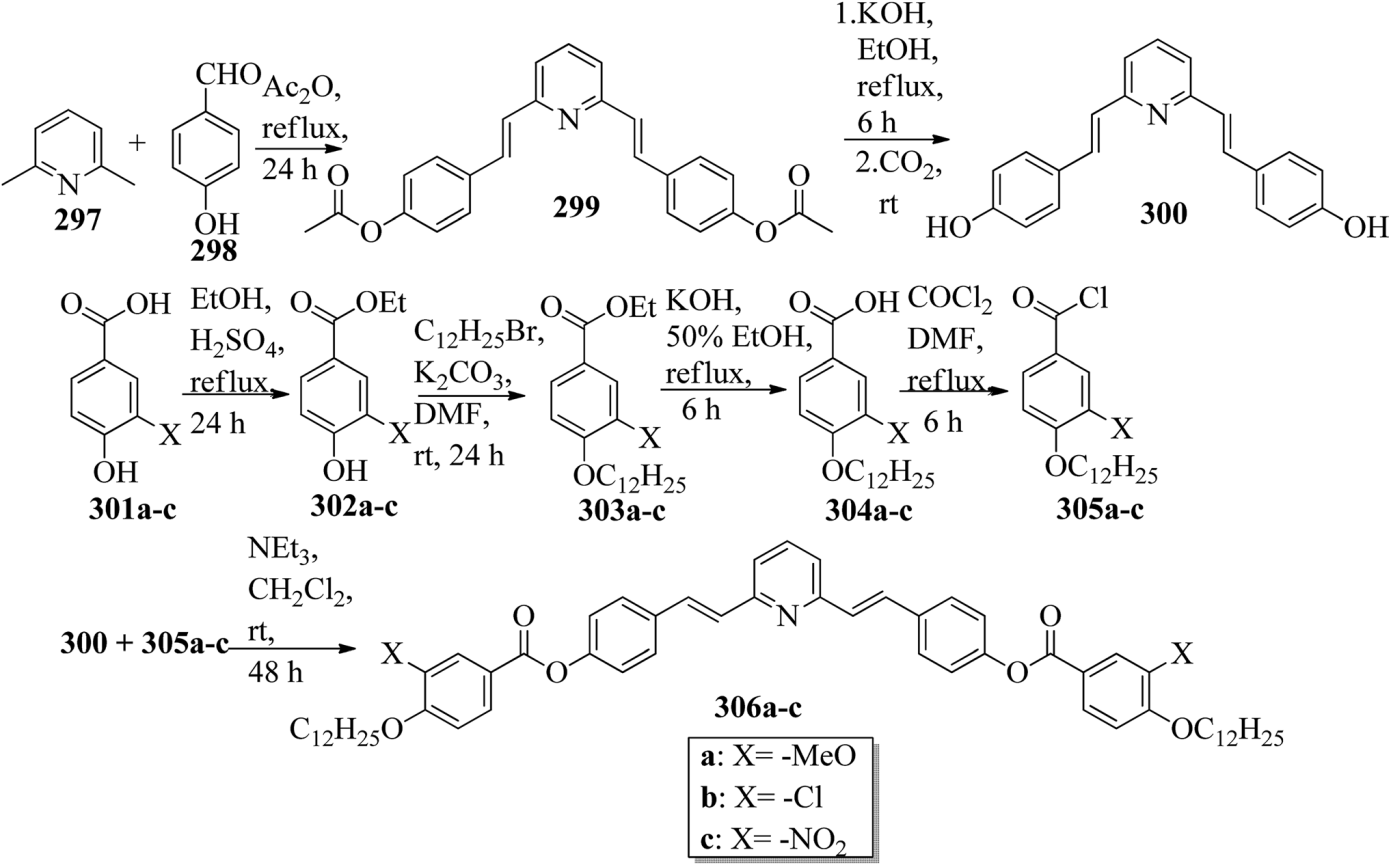
Synthetic route for compounds 306a–c.

**Fig. 17:**
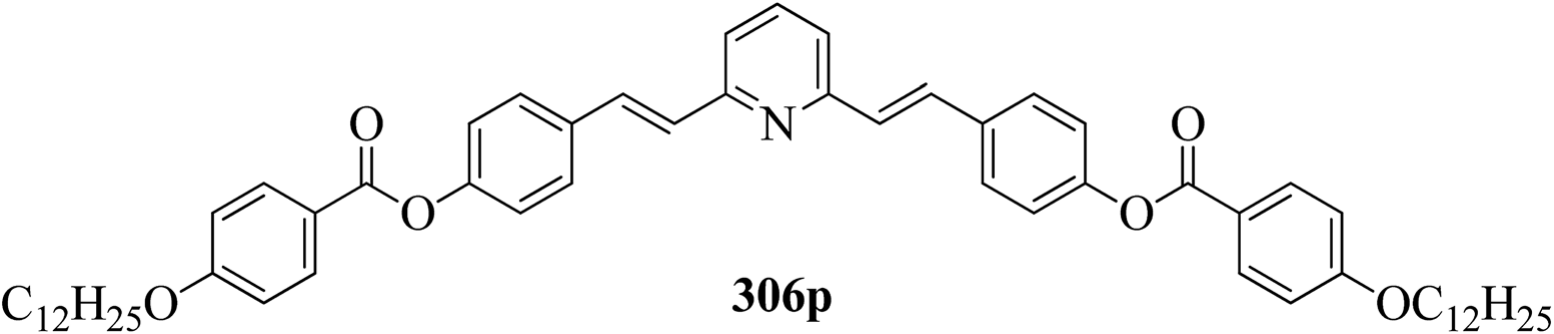
Structure of compound 124p, which was reported by Marković *et al.*^107^

Trišović *et al.*^104^ synthesized photoactive liquid crystals containing five-ring pyridine-based bent-cores with different substituents at the peripheral phenyl rings (–OCH_3_, –Cl and –NO_2_). 2,6-Bis[2-(4-(4-dodecyloxy-3-substituted benzoyloxy)phenyl)ethenyl]pyridines with different substituents, namely methoxy (306a), chloro (306b) and nitro (306c), in the 3^rd^ position of the peripheral phenyl were synthesized as shown in Scheme 38. 2,6-Bis[2-(4-hydroxyphenyl)ethenyl]pyridine (300) was prepared by referring to the procedure by Bergmann and Pinchas.^105^ 2,6-Lutidine (297) was refluxed with excess 4-hydroxybenzaldehyde (298) and acetic anhydride to yield compound 299, of which base hydrolysis led to the formation of compound 300. 4-Dodecyloxy-3-substituted benzoic acids (304a–c) were synthesized by referring to a previous report by Sivakumar *et al.*,^106^*i.e.* protection of the acid group by esterification and alkylation of the hydroxyl group using 1-bromododecane followed by deprotection of the acid group through base hydrolysis. Finally, the target molecules (306a–c) were synthesized by acylation of compound 300 with substituted benzoyl chlorides, *i.e.*305a–c (2 equivalents) in the presence of triethylamine base. The liquid crystal properties of the synthesized compounds were studied using POM, DSC, and XRD and compared with those of the unsubstituted parent compound (Fig. 17), which was reported by Marković *et al.*^107^ Studies revealed that on introducing the methoxy group, the mesogenic properties were quenched and the melting point decreased (110 °C) compared to that of the parent compound. On the other hand, enantiotropic B1-like phase was observed in the chloro- and nitro-substituted compounds. Chloro-substituted compound 306b exhibited a broader mesomorphic range than the parent compound 306p; however, the clearing point was found to be unchanged. On introducing strong electron-withdrawing nitro groups, they noted that the clearing temperature increased by *ca.* 15 °C and a somewhat narrower range of mesomorphic properties than the parent compound was obtained. The smectic phase formation suggests that these compounds exist in s-*cis*/s-*cis* conformation. Further, significant changes in the transition temperatures and textures of the mesophases were observed when the compounds were irradiated by UV light.

**Scheme 39:**
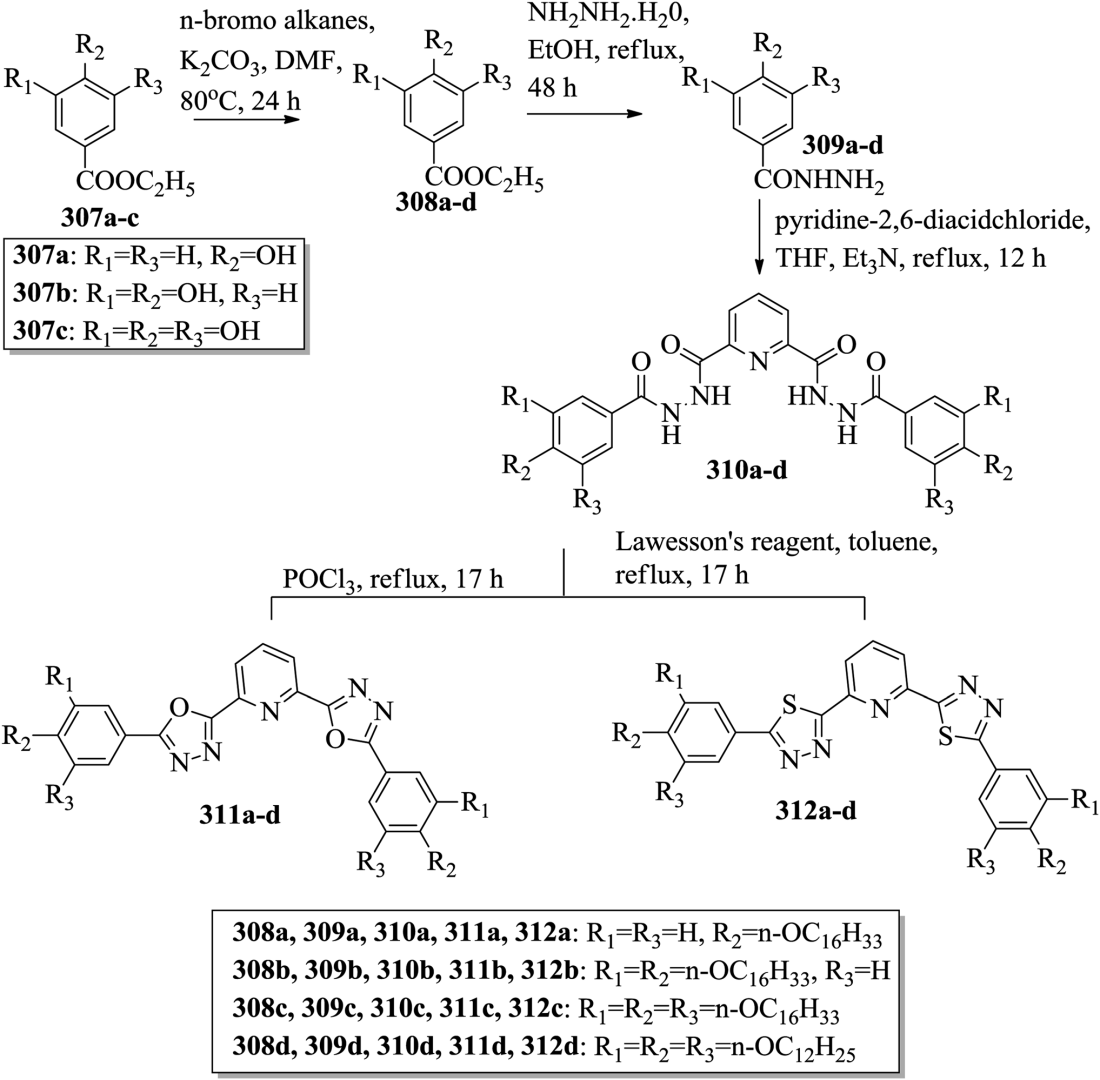
Preparation of 311a–d and 312a–d.

**Fig. 18:**
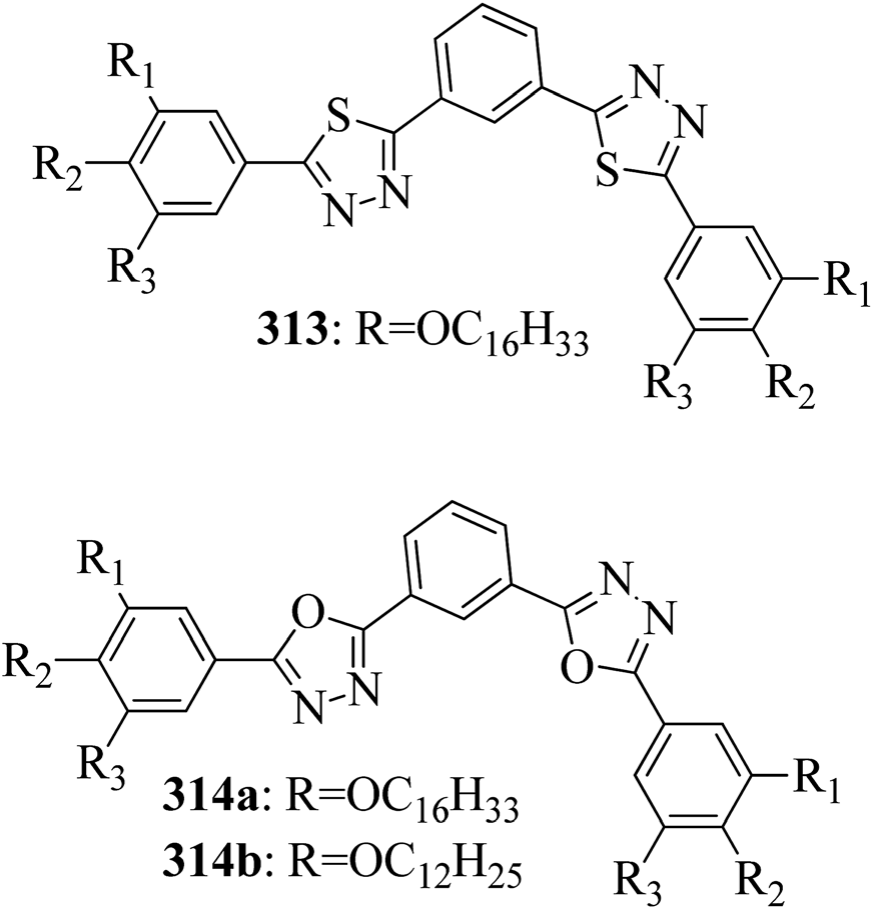
Structures of previously reported benzene-based molecules 313 and 314a–b.

Pradhan *et al.*^108^ synthesized a bent-shaped molecule containing a pyridine core at the centre which was also substituted with thiadiazole or oxadiazole derivatives with different numbers and lengths of alkoxy chains. The expected molecules 311a–d and 312a–d and their precursors were prepared as illustrated in Scheme 39. *O*-Alkylation of the corresponding hydrazides (309a–d) was performed by referring to a previous report by Pradhan *et al.*^109^ 2,6-Di-*N*-pyridinebenzohydrazides 308a–d were synthesized by refluxing these hydrazides with 2,6-pyridine dicarboxylic acid chloride and triethylamine base. Then, cyclization in the presence of phosphoryl chloride (POCl_3_) yielded the dioxadiazole derivatives (311a–d) and cyclization in the presence of Lawesson's reagent in toluene yielded the dithiadiazole derivatives (312a–d). Among dioxadiazole derivatives 311a–d, the derivatives 311a and 311b are non-mesogenic in nature; however, the other two derivatives, *i.e.*311c and 311d, exhibit columnar rectangular and columnar hexagonal phases, respectively. After the first heating cycle, derivative 311c does not form a crystal phase, *i.e.* it continues to exist in columnar phase after cooling to room temperature. Among dithiadiazole derivatives 312a–d, derivative 312a is non-mesogenic in nature, and all the other derivatives exhibit mesophases. Derivatives 312b and 312d exhibited columnar rectangular phases, whereas compound 312c exhibited columnar hexagonal and columnar rectangular phases. In this series, derivatives 312c and 312d exhibited columnar rectangular phases at room temperature after the first heating cycle. They also compared these derivatives with the benzene-based bent-shaped molecules which were reported by Tang *et al.*^46b^ and Pathak *et al.*^110^ The structures of benzene-based molecules 313 and 314a–b are shown in Fig. 18. Benzene-based hexacatenar compound 313 exhibited a narrow range of hexagonal columnar phase and was crystalline at room temperature, while the corresponding hexacatenar 311a displayed a wide range of rectangular columnar phase, including room temperature. Similarly, compound 314a exhibited monomesomorphic oblique columnar phase, in contrast with the compound 312c, which displayed high-stability rectangular columnar and hexagonal columnar mesophases. Oblique columnar phase was exhibited by the benzene-based hexacatenar compound 314b, while rectangular columnar phase was observed in hexacatenar 312d. Finally, they concluded that the stability of the mesophase was enhanced by introducing pyridine as a central core, which also decreased the melting temperature. High stability and a wider range of mesophase were observed in thiadiazole-based hexacatenars in both the pyridine and benzene-based bent molecules.

**Fig. 19:**
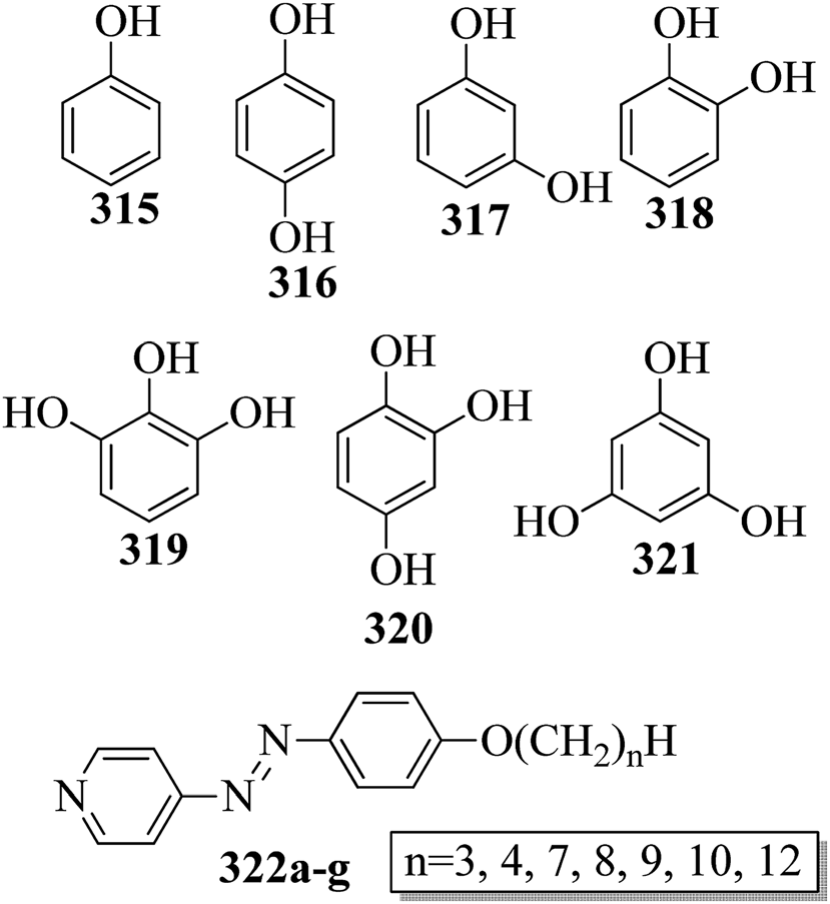
Structures of the core units 315–321 and the proton acceptors 322a–g.

Pfletscher *et al.*^111^ prepared forty-nine hydrogen bonded complexes by mixing hydrogen donor phenol derivatives such as phenol (315), resorcinol (317), catechol (318), hydroquinone (316), hydroxyhydroquinone (320), pyrogallol (319), and phloroglucinol (321) with the corresponding molar ratios of hydrogen acceptor alkoxyazopyridines (322a–g) and dissolving them in acetone solvent, followed by evaporation of the solvent. The structures of the core units (315–321) and the proton acceptors (322a–g) are presented in Fig. 19. None of the starting materials exhibit liquid crystalline properties. Complexes 316/322a–g, 319/322a–g and 320/322a–g do not exhibit mesogenic properties. Because of successive disaggregation of the hydrogen bonded 320/322a–g complexes, they do not exhibit liquid crystalline properties. In complexes 319/322a–g, mesophase was not observed due to incomplete formation of the 1 : 3 complexes. The crystal structures of complexes 316/322a–g revealed a decreased capacity to slip along each other due to the strongly interdentate packed assemblies, which also decreases the required flexibility for the origin of the liquid crystalline state. The linear arrangements of 316/322a–g and 317/322a–g favor the formation of crystalline and smectic phases, respectively. Dissimilarly bent-shaped structures of 318/322a–g and 319/322a–g resulted in nematic mesophases. When *n* = 7, all the complexes are non-mesogens except for complex 321/322c. In complexes 315/322a–g, only the complex with *n* = 9 (315/322e) exhibited smectic phase; all the other complexes are non-mesogens. As the chain length increases in the 318/322a–g complexes, the mesophase stability decreases from Δ*T* = 13.6 °C to Δ*T* = 6.8 °C. However, in the 317/322a–g complexes, the mesophase stability was found to increase from Δ*T* = 6.3 °C to Δ*T* = 21.7 °C as the chain length increased. They also studied the photo-responsive behavior of the hydrogen bonded liquid crystals, which revealed that on irradiation of light at 405 nm, a reversible phase transition occurred from the mesophase into the isotropic phase, where the speed of interchange depends on the morphologies of the phases. Smectic mesophase responded remarkably more slowly than the nematic mesogens. This outcome indicated that separation of the aromatic and aliphatic fragments, as noted in the solid state structure, sustained the generation of stable mesophases.

### 2.4. Pyridine-based polycatenar mesogens

Polycatenar mesogens are a hybrid class of thermotropic liquid crystalline materials which have molecular features of both calamitic and discotic mesogens. Here, the central core has a rod-type molecular structure and two half disc-like structures at both ends. There are different types of polycatenar LCs based on the number of aliphatic chains on both sides of the molecules. The numbers of chains on both sides of the core vary from two to six. In this section, we discuss the available synthetic routes for the preparation of pyridine-based polycatenar mesogens and their properties.

**Scheme 40:**
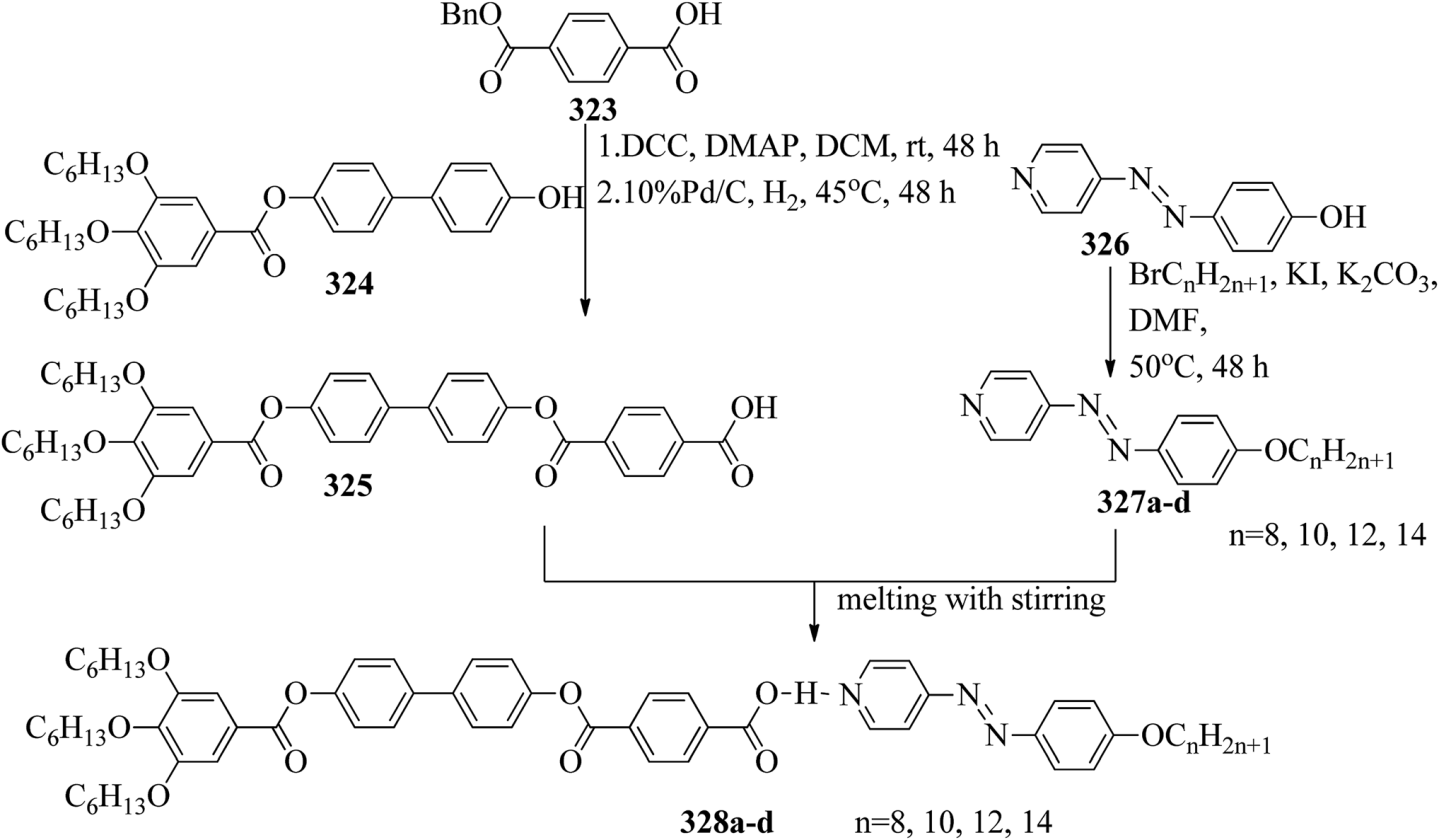
Synthetic route for the preparation of hydrogen bonded complexes 328a–d.

Alaasar *et al.*^41^ designed and synthesized hydrogen bonded supramolecular complexes with polycatenar structures exhibiting high-powered mirror-symmetry shattering by chirality synchronization in chiral “*Im*3̄*m* type” cubic phase (Cub^[^*^]^/*I*432) as well as in a liquid conglomerate (Iso_1_^[^*^]^) at the liquid–liquid transition. Benzoic acid derivatives 325 and azopyridines 327a–d were synthesized as shown in Scheme 40. The benzoic acid 325 displays a hexagonal columnar phase between 162 °C and 246 °C, and 4-(4-alkyloxyphenylazo)pyridines 327a–d are non-mesomorphic in nature. Hexagonal columnar phase was observed in the case of 325, which was ascribed to the formation of dimers by intermolecular hydrogen bonding between the –COOH groups. An optical negative hexagonal columnar phase was developed due to alignment of the rod-like cores perpendicular to the long axis in the columns. The hydrogen bonded supramolecular complexes 328a–d were prepared by mixing equimolar amounts of 327a–d and 325 followed by melting with stirring. After the crystallization, the product was ground and the process was repeated to afford a homogeneous mixture. All the supramolecular complexes 328a–d exhibited reproducible transition temperatures and homogenous melting. In the supramolecular complexes, a broad cubic liquid crystal phase was induced by suppressing the columnar phases. The complexes 328b–d exhibited cubic phases, whereas in complex 328a, an additional liquid–liquid transition (Iso–Iso_1_ transition) was observed. The liquid phases Iso and Iso_1_ appeared uniformly dark between the crossed polarizers. However, a gentle rotation of the analyzer by negligible degrees (*ca.* −7°) out of the 90° direction in the Iso_1_ phase range with respect to the polarizer results in the occurrence of dark and bright zones; by gyration of the analyzer at the same angle in the opposite direction (*ca.* +7°), the brightness of the zones is the opposite. No changes were observed when rotating the sample between crossed polarizers; this indicated that distinct regions exhibit chiral domains. This provides obvious evidence for chirality synchronization in the Iso_1_ phase (Iso_1_^[^*^]^). In the Iso phases of achiral complexes 328b–d and the Iso phase of 328a at higher temperature, this type of domain was not observed. On melting the crystalline phase at *T* = 124 °C, achiral *Ia*3̄*d* phase was formed, which transformed into chiral *I*432 phase on heating at *T* = 180 °C to 187 °C. Optical studies revealed that increasing the alkyl chain length decreases the helical pitch, which becomes irreconcilable with the structure of *Ia*3̄*d* and leads to the emergence of *I*432 cubic phase. In the cases of molecules with shorter chain lengths, liquid miscellany was formed, and the desegregation in the midst of the chain and core units was adequately fragile to hinder the development of the long range cubic lattice.

Recently, Pană *et al.*^112^ prepared flexibly linked bis(pyridinium) salts bearing different counter ions such as Br^−^, PF_6_^−^, BF_4_^−^ and OTf^−^ using *N*-alkylated 4-pyridones and 3,4,5-tris(alkyloxy)benzyl moieties. All the prepared bis(pyridinium) salts exhibited an enantiotropic hexagonal columnar mesophase except the salt of triflate ion, which contains a shorter terminal carbon chain.

### 2.5. Pyridine-based polymeric mesogens

Polymeric mesogens are compounds that blend the properties of polymers with those of mesogens. These mesogens exhibit the anisotropic properties of liquid crystals but retain the unique properties of polymer compounds, such as ultra-thin film forming ability and high strength. Compared to conventional polymers, these materials exhibit excellent mechanical properties and chemical stabilities. In this section, we discuss the available synthetic routes for the preparation of different pyridine-based polymeric mesogens and their properties.

**Scheme 41:**
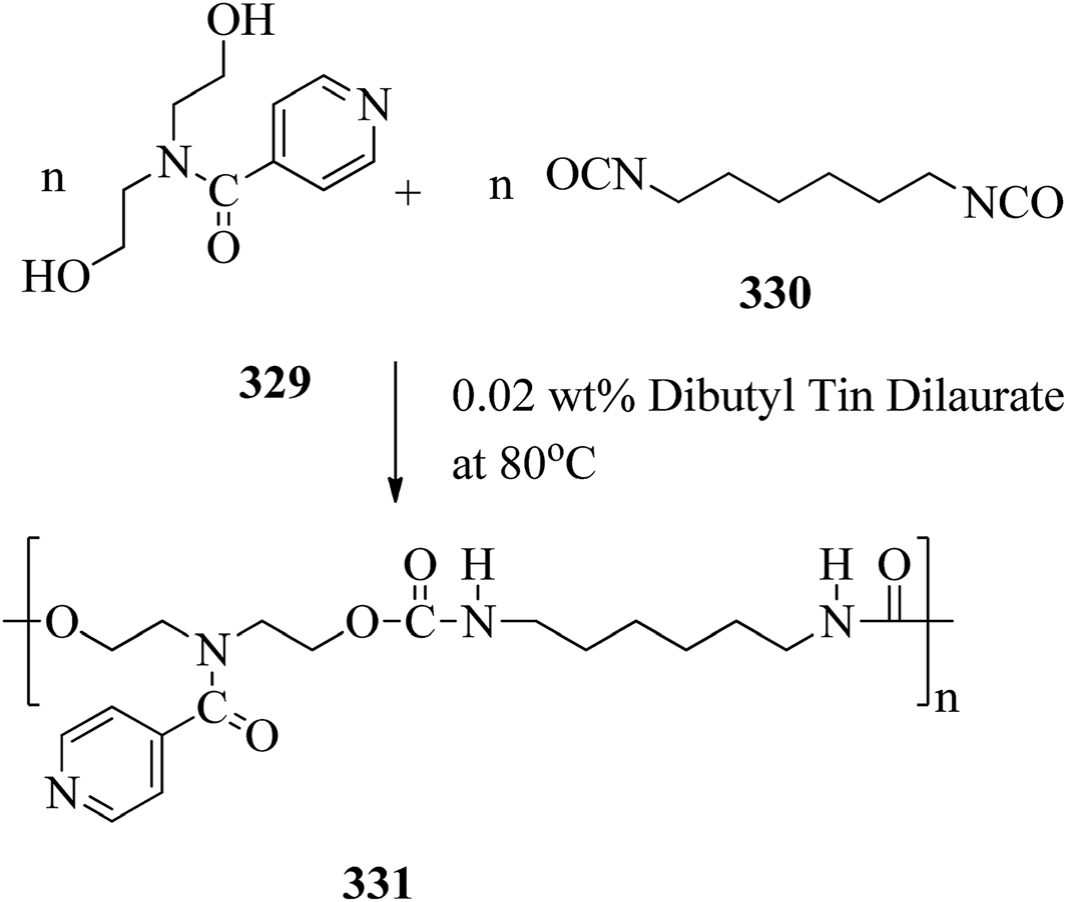
Synthetic route for 331.

**Fig. 20:**
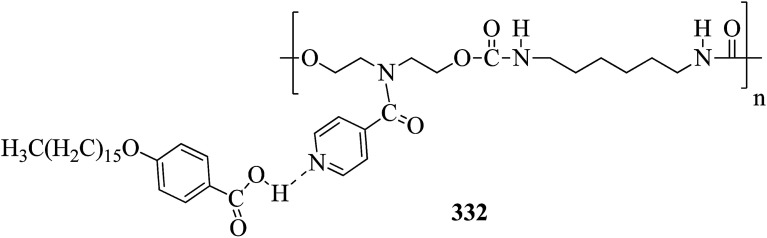
Supramolecular structure of 332.

**Fig. 21:**
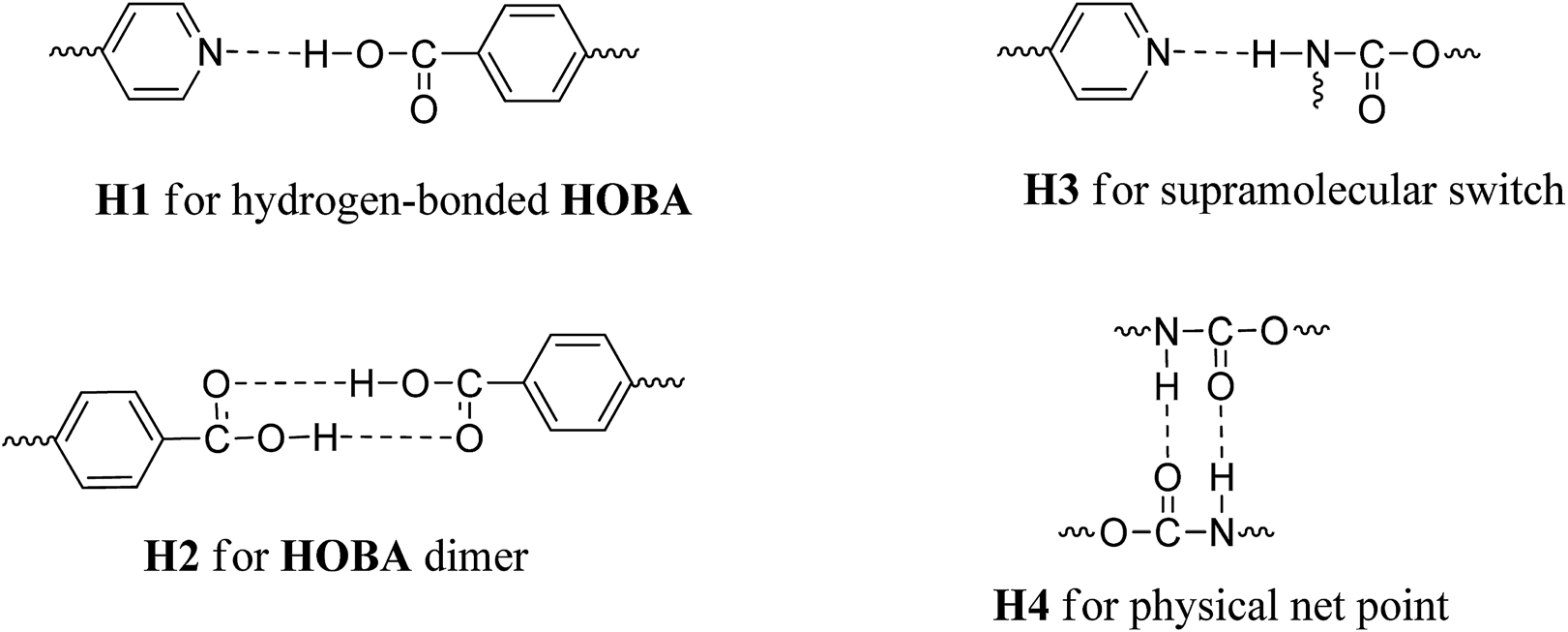
Other possible hydrogen bonds in supramolecular complex 332.

Chen *et al.*^113^ synthesized a polymer formed by amide linkages containing pyridine and a shape memory polyurethane (331) polymer by treating 1,6-hexanediisocyante (330) with *N*,*N*-bis(2-hydroxylethyl)isonicotinamine (329) at 80 °C in dimethyl formamide (DMF) solvent using 0.02 wt% dibutyltin dilaurate as a catalyst (Scheme 41). Further, a series of hydrogen bonded complexes (332) were prepared by mixing 331 and 4-hexadecyloxybenzoic acid (HOBA) at different mole ratios of HOBA/331 in DMF solvent for a specified time, followed by solvent drying under vacuum. The formed supramolecular structure of 332 is shown in Fig. 20. Furthermore, the formation of hydrogen bonds between 331 and HOBA was confirmed by comparing the FT-IR spectra of the parent compounds as well as the prepared hydrogen bonded complexes. These complexes exhibited both shape memory and liquid crystalline properties. The hydrogen bonded complexes maintained the intrinsic smectic C phase of HOBA at all the mole ratios; a high mole ratio resulted in clearer smectic C phase and crystalline phases because the sample not only contained hydrogen-bonded HOBA complexes but also HOBA dimers. The morphological examinations showed that complex 332 forms two-phase separated structures due to the presence of an amorphous polyurethane matrix and a HOBA crystalline phase; this may originate from the hydrogen-bonded interactions and free HOBA. Fig. 21 shows the other hydrogen bonds which may occur in supramolecular complex 332.

**Scheme 42:**
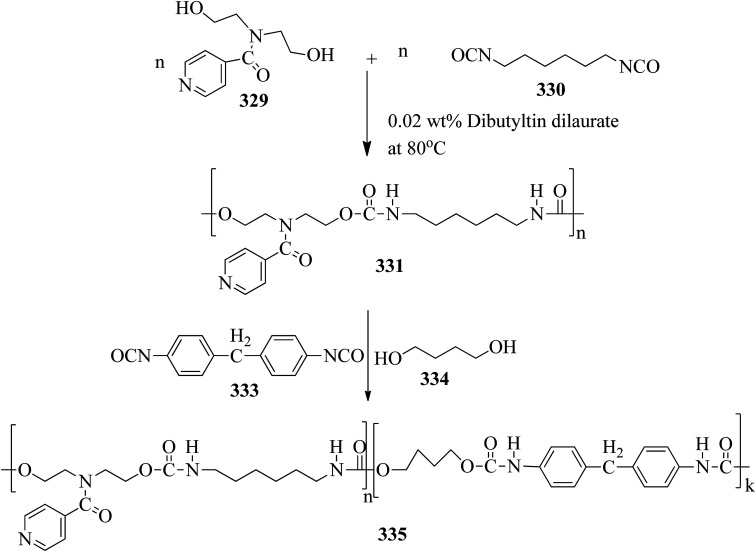
Synthesis of polymer 335.

**Fig. 22:**
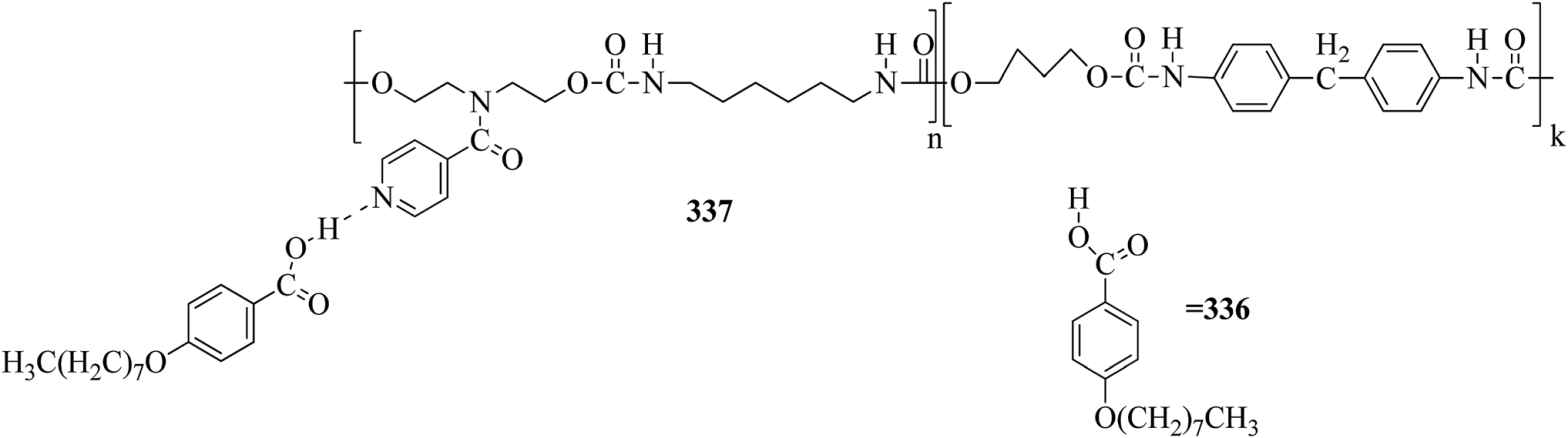
Hydrogen bonded complex 337.

Chen *et al.*^114^ designed and synthesized a supramolecular liquid crystalline shape-memory polyurethane complex (337) by fusing 4-octyloxybenzoic acid (336) with a polymer (335) by hydrogen bonding interactions; they also prepared complexes with different mole ratios (0.10, 0.23, 0.38, 0.58, and 0.86 wt%) of 336/335. These complexes exhibited both multi-shape memory and liquid crystalline properties. The synthesis of compound 335 is given in Scheme 42. The synthesis of polymer 335 was accomplished using 1,6-hexanediisocyante (330) with *N*,*N*-bis(2-hydroxylethyl)isonicotinamine (329) at 80 °C in DMF solvent using 0.02 wt% dibutyltin dilaurate as a catalyst, followed by extension of the chains by adding diphenylmethane diisocyanate (333) and 1,4-butanediol (334) to the reaction mixture; to control the viscosity of the reaction, DMF was added occasionally for another 2 h, and the reaction was maintained for 4 h to obtain a solution of 10-wt% 335/DMF. Further, this compound was used to form hydrogen bonds with 336 at different mole ratios of 336/335 (0.10, 0.23, 0.38, 0.58, and 0.86 wt%). These mixtures were stirred for 2 h to form homogenous solution-phase mixtures; finally, each mixture was added to a Teflon pan and incubated for 24 h at 80 °C, then dried under a vacuum of 0.1 to 0.2 kPa at 80 °C for 24 h. Fig. 22 presents the hydrogen bonded complex 337. The crystallization peak appropriately increases as the content of 336 increases. In the higher 336 content complexes, there is a phase transition from nematic to smectic phase; this was identified at 102 °C to 115 °C, in addition to a 2^nd^ crystallization peak at 94 °C. Thus, it is confirmed that in the prepared complexes, both the liquid crystalline and crystalline properties of 336 were maintained. Shape memory investigation indicated that these complexes have good multi-shape memory effects, incorporating triple-shape memory and quadruple-shape memory behaviors. Because of the combination of multi-shape memory effects and liquid crystalline properties in these complexes, they are potential candidates for applications in smart sensors, smart electronics and smart optical devices.

**Scheme 43:**
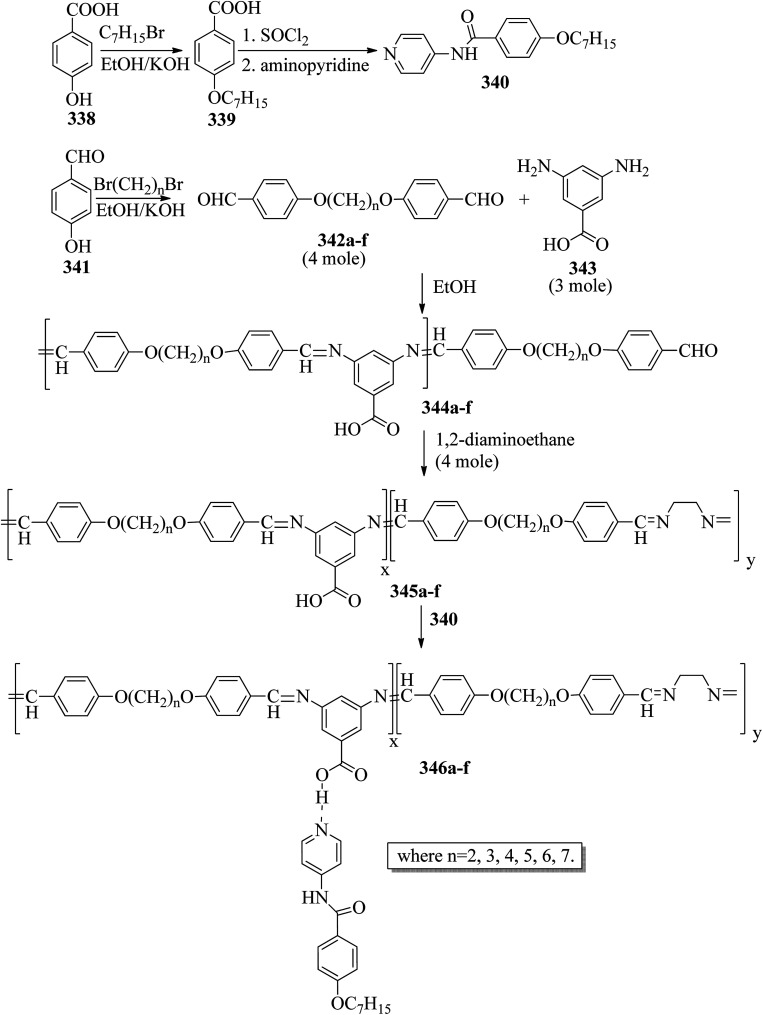
Synthesis of hydrogen bonded complexes 346a–f.

Al-Lami^115^ prepared two block copolymers which show supramolecular liquid crystalline properties. 4-Heptyloxy benzoic acid (339) was synthesized using 1-bromoheptane and 4-hydroxy benzoic acid (338). 4-Heptyloxy-*N*-pyridine-4-yl-benzamide (340) was synthesized by refluxing 339 with excess thionyl chloride in dry N_2_ atmosphere for 5 h. Under reduced pressure, the excess thionyl chloride was removed. The obtained acid chloride and 4-aminopyridine were later dissolved in dry pyridine and stirred at room temperature for 24 h, followed by pouring the reaction mixture into dilute hydrochloric acid to obtain the crude product. The obtained crude product was filtered and recrystallized using ethanol. On the other hand, α,β-bis(4-formyl phenyl-4-oxy)alkanes (342a–f) were prepared by refluxing the required dibromo alkane, 4-hydroxy benzaldehyde (341) and KOH for 24 h. The crude product was filtered and recrystallized using ethanol. Further, by referring to the report by Ignatious *et al.*,^116^ the authors prepared a block copolymer series (344a–f) by refluxing the monomer 2,3,5-diamino benzoic acid (343) and a catalytic amount of glacial acetic acid in ethanol solvent for 2 h. To the same reaction mixture, 1,2-diaminoethane was added slowly, and the mixture was refluxed for 2 h to afford the block polymers 345a–f. The obtained products were filtered and re-crystallized using ethanol solvent. Furthermore, the hydrogen bonded complexes were prepared by mixing carboxylic acid containing the block copolymer (345a–f) and pyridine-containing monomer (340) in an equimolar ratio; this mixture was dissolved in pyridine, followed by removal of the solvent under vacuum for several days at 80 °C (Scheme 43). These prepared copolymers have two different flexible spacer lengths, in which the first spacer is fixed at two methylene groups while the other spacer varies from two to seven groups. Using DSC and POM, the mesophase properties of the copolymer were studied. These studies revealed that all the copolymers exhibit nematic phase. It was also found that even-numbered chain lengths have higher transition temperatures than odd-numbered chain lengths. Further, the clearing temperature and melting point were found to gradually decrease as the flexible spacer length increased, in which even members showed slightly higher values. The decreases in the melting point and isotropic temperature are due to the increase in the flexible spacer chain length. Also, the number of possible conformations increased, which resulted in distortion of the cylindrical shapes of the mesogens.

**Fig. 23:**
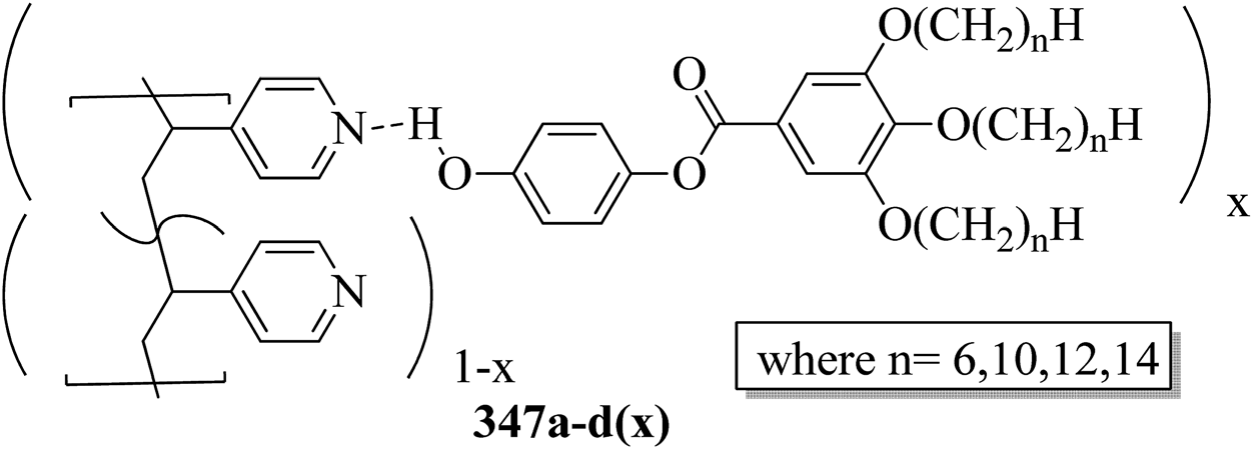
Hydrogen bonded complexes 347a–d(x).

Wang *et al.*^117^ prepared a series of supramolecular polymer complexes, 347a–d(x). These complexes were prepared by dissolving polymer poly(4-vinylpyridine) (P4VP) and dendron-like small molecules containing phenolic end groups (*n*CTB, *n* = 6, 10, 12, 14) separately in chloroform; then, these two solutions were mixed to form 5 wt% solutions and stirred for 12 h at room temperature. The resulting complexes were maintained at 30 °C for 24 h in a vacuum oven. Fig. 23 presents the hydrogen bonded complexes 347a–d(x). These complexes exhibited supramolecular lamellar and hexagonal columnar structures, the stability of which depends on the alkyl chain length, the blending ratio of *n*CTB per vinylpyridine unit (x) and the thermal treatment. 347a(x) exhibits only poorly ordered lamellar phase. However, in the other complexes 347b–d (*n* = 10, 12, 14), the lamellar structure transformed into a hexagonal columnar structure as x increased. When the volume fraction of the alkyl tails was about 0.56 to 0.58, a transition occurred from lamellar to hexagonal columnar phase (a non-reversible order-to-order transition) during the thermal annealing. This is due to the conformational changes in P4VP and the thermal dynamic nature of the hydrogen bonds.

**Scheme 44:**
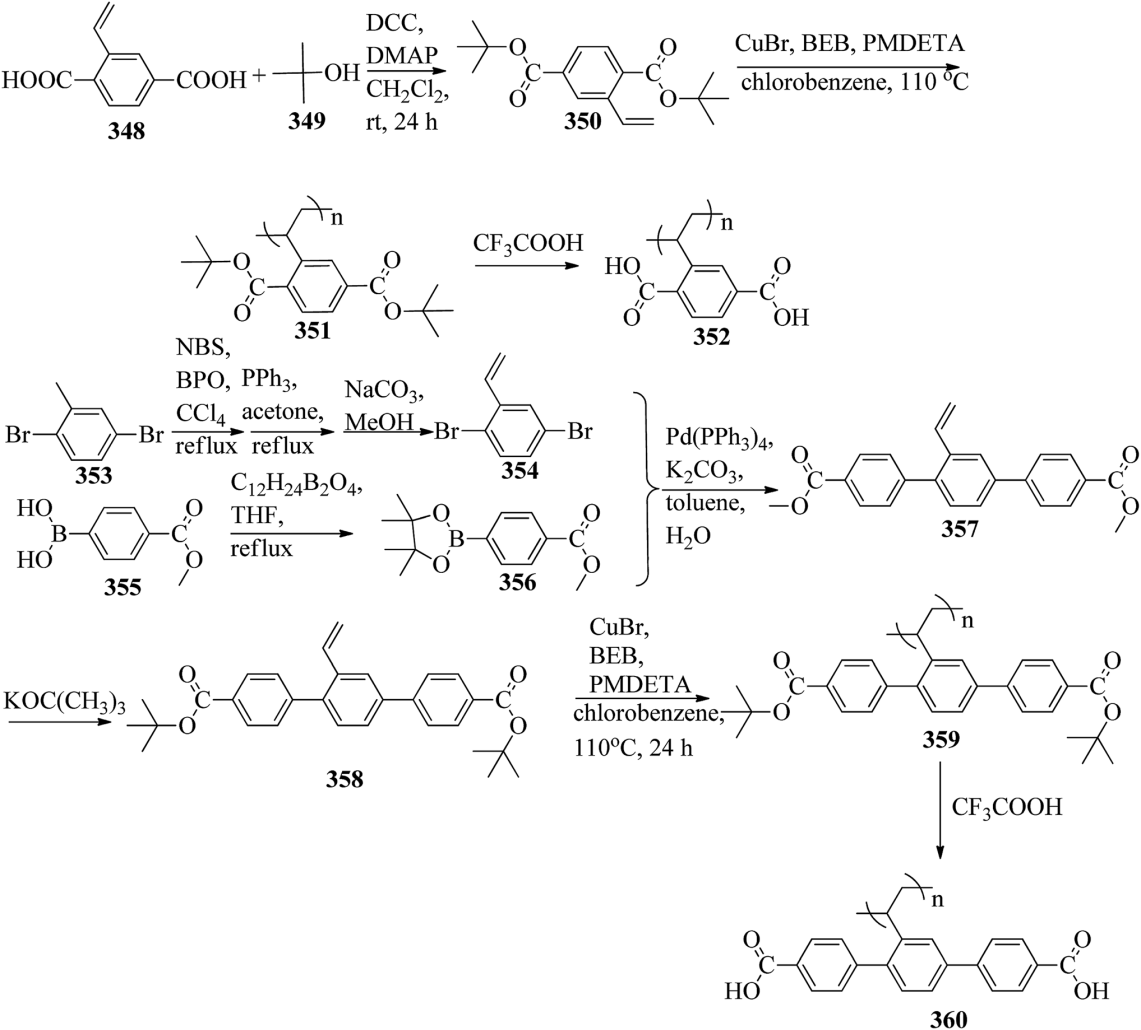
Synthetic route for the polymers 352 and 360.

**Scheme 45:**
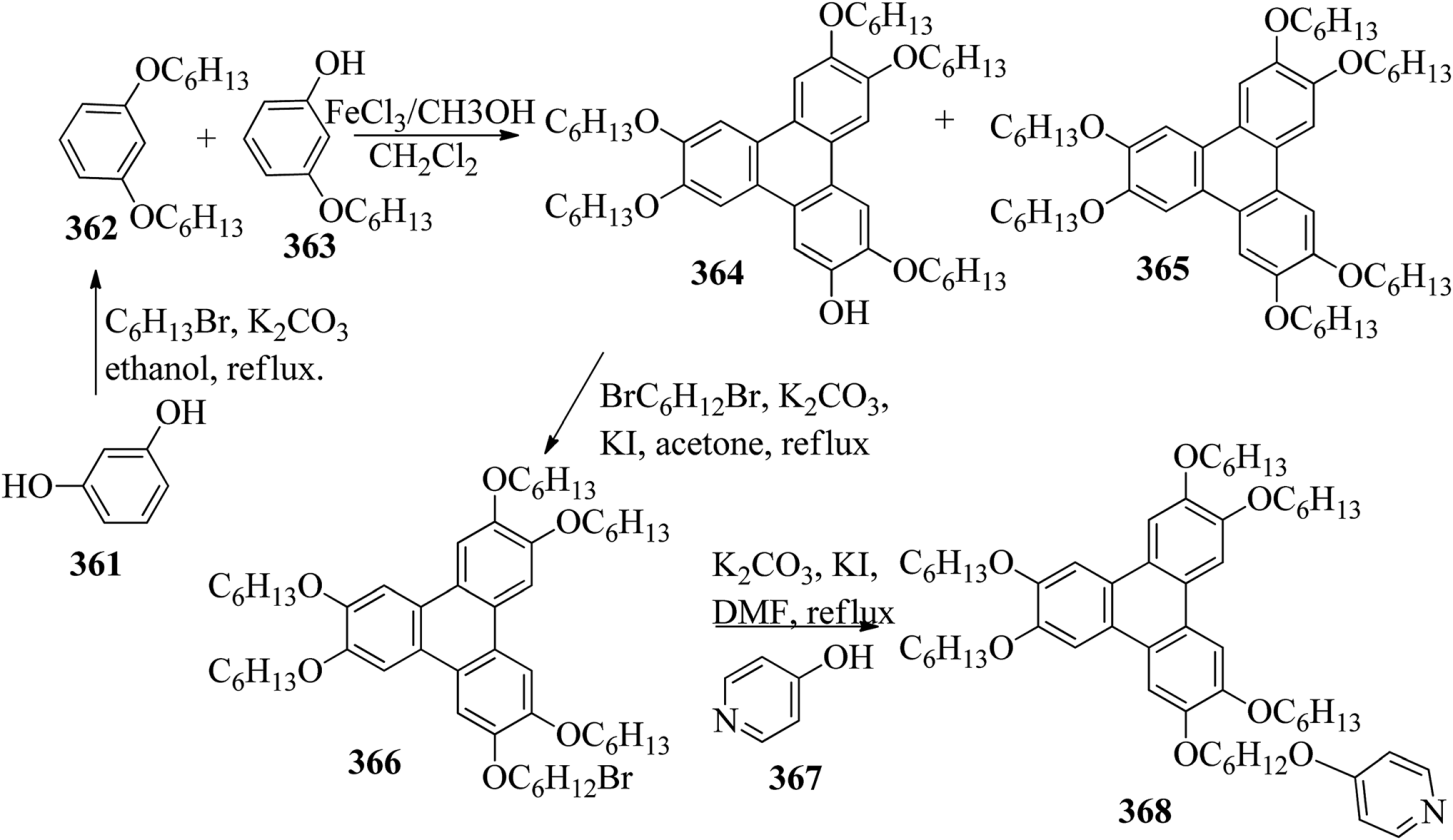
Synthetic route for compound 368.

Yang *et al.*^118^ synthesized main-chain/side-chain combined liquid crystalline polymers containing mesogen-jacketed liquid crystalline polymers (MJLCPs) as the main chains. In these polymers, a pyridine derivative with a triphenylene (Tp) unit was used as the hydrogen bond acceptor. 2-Vinylbenzene-1,4-dioic acid (348) was synthesized by referring to a previous report by Zhang *et al.*,^119^ and it was stirred at room temperature with 2-methylpropan-2-ol (349), *N*,*N*-dimethylpyridin-4-amine, and *N*,*N*′-dicyclohexylcarbodiimide in dichloromethane solvent for 24 h. The insoluble matter was removed by filtration and the solvent was evaporated under reduced pressure to afford di-*tert*-butyl 2-vinylterephthalate (350). 2,3-Dimethyl-2,3-butanediol was refluxed with 4-(methoxycarbonyl)phenylboronic acid in THF solvent for 2 h at 70 °C and the solvent was evaporated under reduced pressure to afford methyl 4-(4,4,5,5-tetramethyl-1,3,2-dioxaborolan-2-yl)benzoate. 2,5-Dibromostyrene (354) was synthesized by referring to a previous report by Qu *et al.*;^120^ then, it was taken in a three-necked flask to which 4-methyl-(4,4,5,5-tetramethyl-1,3,2-dioxaborolan-2-yl)benzoate, hydroquinone, potassium carbonate and Pd(PPh_3_)_4_ were added. The reaction was performed under a continuous stream of argon, and water and toluene were injected during the course of the reaction. Later, this reaction mixture was stirred for 48 h at 110 °C. The separated organic layer was dried using anhydrous magnesium sulphate (MgSO_4_) and the solvent was evaporated under reduced pressure to afford 2,5-bis(4-methoxycarbonyl phenyl)styrene (357). Further, this compound was dissolved in ether and potassium *tert*-butoxide was added slowly. The mixture was stirred for 2 h at ambient temperature, washed with water and dried using MgSO_4_. The solvent was evaporated after filtration to afford 2,5-bis(4-*tert*-btuoxylcarbonyl phenyl)styrene (358). *N*,*N*,*N*′,*N*′′,*N*′′-Pentamethyldiethylenetriamine (PMDETA), monomer 350, (1-bromoethyl)benzene, dry chlorobenzene, and CuBr were taken in a polymerization tube. The tube was sealed under vacuum after three freeze–pump–thaw cycles and later maintained in a thermostatted oil bath for 24 h at 110 °C. The tube was quenched in liquid nitrogen to stop the polymerization and was then maintained in ambient conditions. The solution was diluted using THF solvent, and copper salt was removed by passing it through the alumina column. Finally, 359 was precipitated from methanol and dried. Then, it was dissolved in chloroform, and trifluoroacetic acid was added slowly at ambient temperature. The solution was maintained for 24 h with stirring and the solvent was evaporated under reduced pressure to afford polymer 360 (Scheme 44). By referring to the report by Xing *et al.*,^121^ 2-hydroxyl-3,6,7,10,11-pentakis(hexyloxy)triphenylene (364) was synthesized, and it was refluxed with 1,6-dibromohexane, potassium iodide, and potassium carbonate in acetonitrile solvent. After the reaction, the mixture was filtered and the filtrate was collected; the solvent was evaporated under reduced pressure to afford compound 366, followed by refluxing compound 366 with 4-hydroxy pyridine (367) in the presence of potassium iodide and potassium carbonate in DMF solvent to yield compound 368 (Scheme 45). Finally, the expected complexes were prepared by mixing different molar ratios of 368 to the –COOH groups in the repeating units in pyridine solvent and stirring for 24 h at 60 °C; then, the solvent was allowed to evaporate slowly for 3 days at 50 °C, and the product was dried under vacuum at 60 °C for another 24 h. The phase behavior of the synthesized complexes strongly depends on the rigidity of the side-chain core of the MJLCPs and the amount of hydrogen bond acceptor. In the 352/368x complex, the simple columnar phase transforms to a hierarchical nanostructure containing a hexagonal columnar phase arising from the entire polymer chain and a discotic nematic phase assigned to the Tp moieties. Meanwhile, in the 360/368x complex, the simple smectic A phase transforms into a hierarchical nanostructure containing a smectic A phase arising from the entire polymer chain and a discotic nematic phase assigned to the Tp moieties when x is increased to 1. Hence, they suggested that when the 368 content is comparatively low, weak interactions between the Tp moieties cause the complexes to behave like independent MJLCPs, and that the Tp moieties can be strongly considered as side chain constituents of the MJLCPs. The interactions between the Tp moieties become stronger as the 368 content increases, and both types of mesogens can act separately to exhibit their solitary self-organizing structures.

**Scheme 46:**
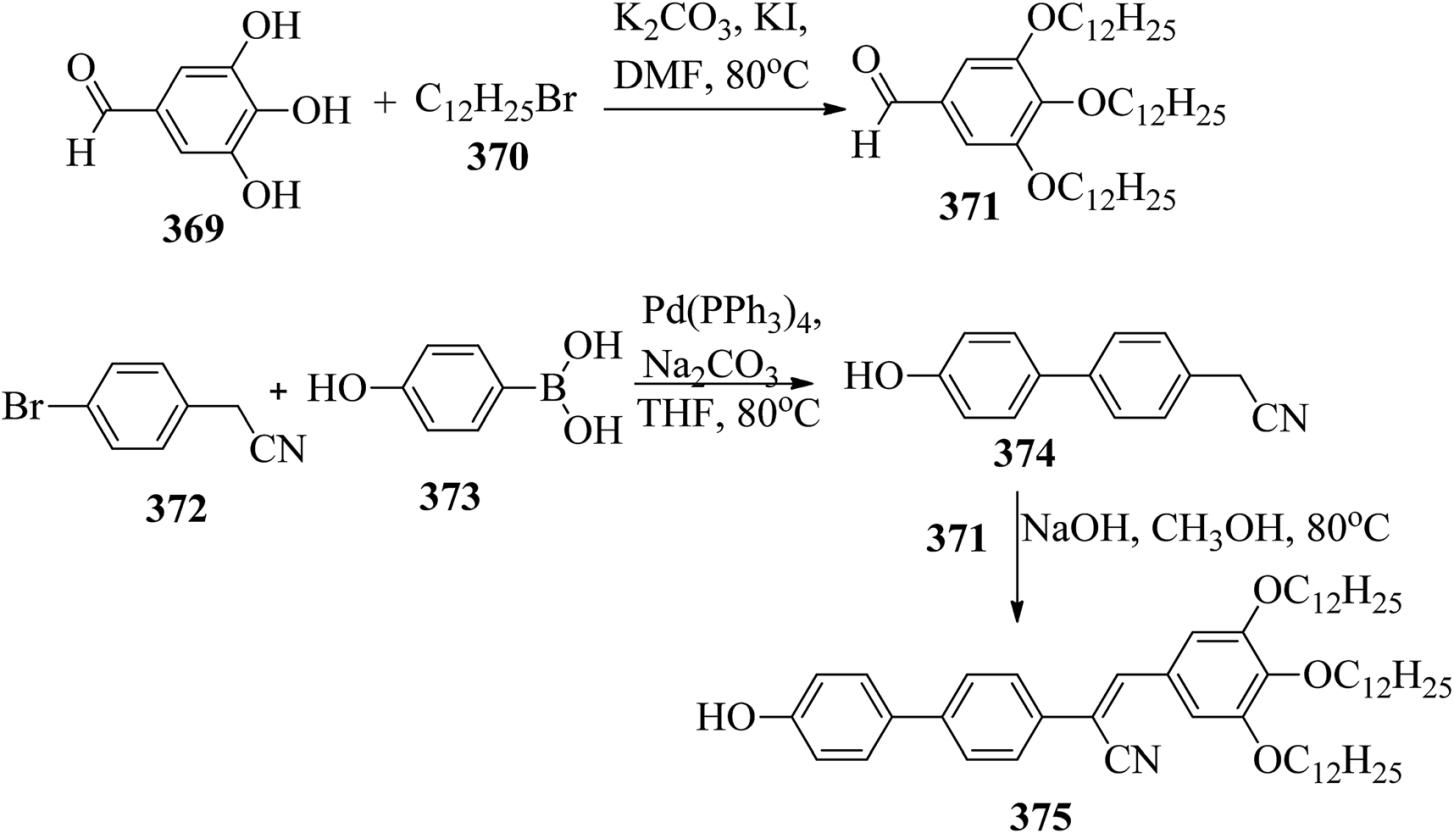
Synthesis of 375.

**Scheme 47:**
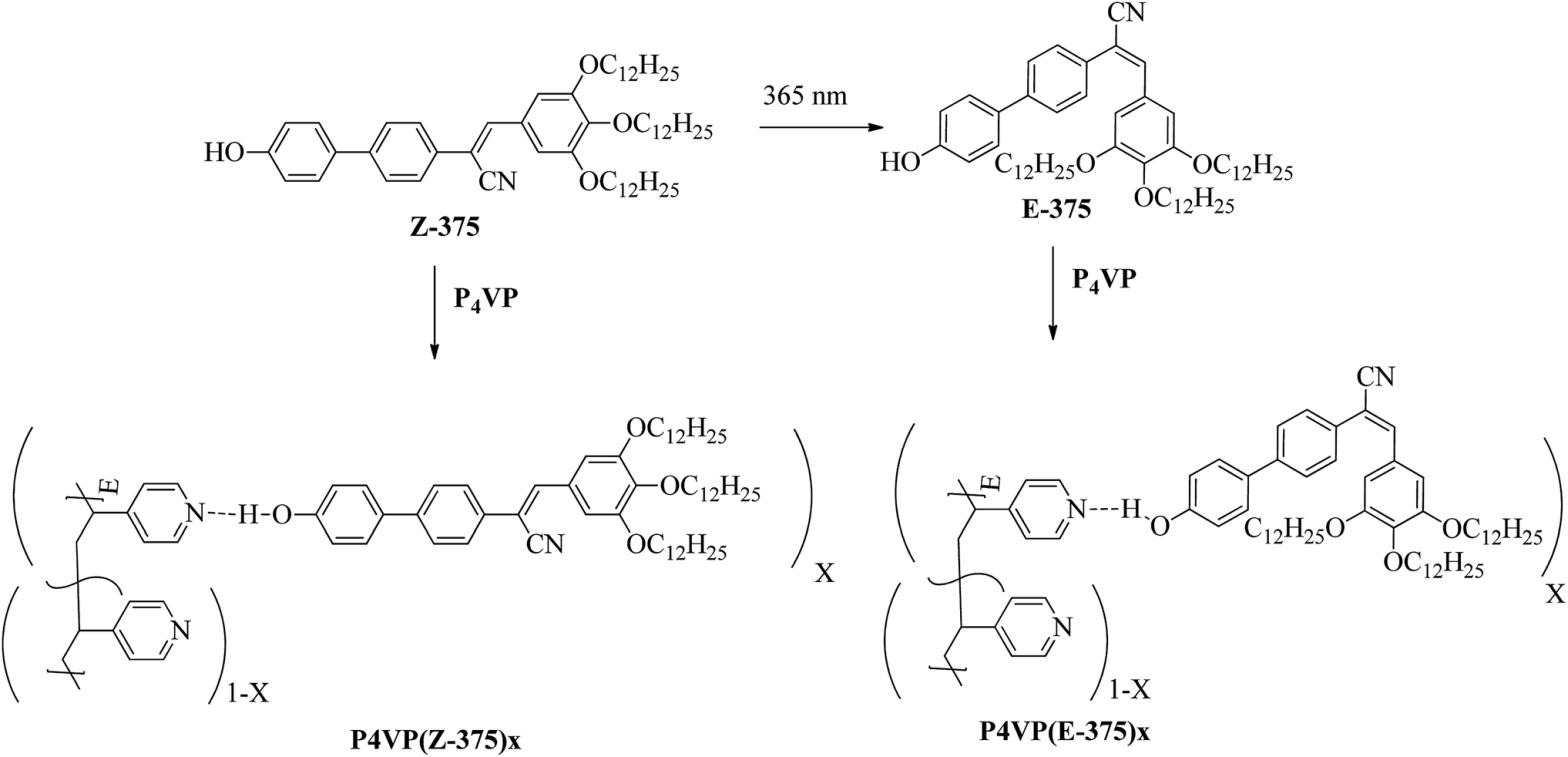
Synthesis of P4VP(*Z*-375)*x* and P4VP(*E*-375)*x*.

Zhu *et al.*^122^ prepared α-cyanostilbene containing hydrogen bonded supramolecular polymers. 3,4,5-trihydroxybenzaldehyde (369) monohydrate was dissolved in dry DMF; to this solution, K_2_CO_3_ and KI were added, and the mixture was stirred at 80 °C. To this reaction mixture, 1-bromododecane (370) was added dropwise; then, the reaction was continued overnight. After completion of the reaction, the reaction mixture was added to brine solution and extracted using dichloromethane. Using magnesium sulphate, the organic layer was dried, and the solvent was evaporated using a rotary evaporator. Finally, the pure 3,4,5-tris(dodecyloxy)benzaldehyde (371) was obtained by column chromatography. 4-Hydroxybenzeneboronic acid (373) and 4-bromophenylacetonitrile (372) were dissolved in THF; later, sodium carbonate solution was added. Under N_2_, Pd(PPh_3_)_4_ was added, and the reaction mixture was refluxed for 12 h. After completion of the reaction, the reaction mixture was cooled to room temperature; then, the mixture was neutralized with HCl. The product was extracted using brine/ethyl acetate, and the organic phase was dried using anhydrous magnesium sulphate. Finally, pure (4′-hydroxybiphenyl-4-yl)acetonitrile (374) was obtained by column chromatography. Compound 371, compound 374, and sodium hydroxide were dissolved in anhydrous methanol and stirred for 12 h at 50 °C under nitrogen atmosphere. After completion of the reaction, the reaction mixture was cooled to room temperature; then, it was neutralized using hydrochloric acid to obtain the product (*Z*)-2-(4′-hydroxybiphenyl-4-yl)-3-(3,4,5-tris-(dodecyloxy)phenyl)acrylonitrile (*Z*-375) (Scheme 46). Irradiating a concentrated solution of *Z*-375 in CHCl_3_ with UV light of 365 nm for 3 h yielded a mixture of *Z*-375 and E-375. Using column chromatography, *E*-375 was separated. P4VP and 375 (*Z*- or *E*-isomers) were dissolved in chloroform separately to obtain solutions of 5 wt%. 375 and P4VP solutions were mixed in appropriate quantities according to x in order to obtain P4VP(375)*x* aggregations, which were then stirred at room temperature for 24 h. After completion of the reaction, the solvent was evaporated slowly and the product was maintained in a vacuum oven at 35 °C. Thus, the supramolecular complexes were obtained (Scheme 47). Cylindrical assemblies formed in the *Z*-375-based complexes due to the wrapping of hydrogen-bonded dendritic *Z*-375 molecules around the P4VP chains. The hexagonal packing was due to the parallel arrangement of supramolecular cylinders. Columnar packing was not favored by the bent-shaped *E*-isomer. The lamellar arrangement was ascribed to the micro-phase partition between the aliphatic non-polar part and aromatic polar part in the polymer aggregations. At the same time, the hydrogen-bonding interactions were hindered to some extent due to the bulky shape of the *E*-isomer. The corresponding P4VP(*Z*-375)*x* and P4VP(*E*-375)*x* aggregates exhibited divergent stimuli-responsive properties and self-assembly behavior. P4VP(*Z*-375)*x* exhibited a hexagonal columnar structure at 0.4 ≤ *x* ≤ 1.0; at the same time, a lamellar structure developed for P4VP(*E*-375)*x* at 0.3 ≤ *x* ≤ 0.7. The P4VP(*Z*-375)0.5 film exhibited irreversible switching and increased fluorescence emission upon UV illumination. Thus, by controlling the configuration of molecules in supramolecular polymer systems, the functional responsive behaviors and phase structures can be readily tuned at the same time.

## 3. Optical textures and temperature ranges of the mesophases

**Fig. 24:**
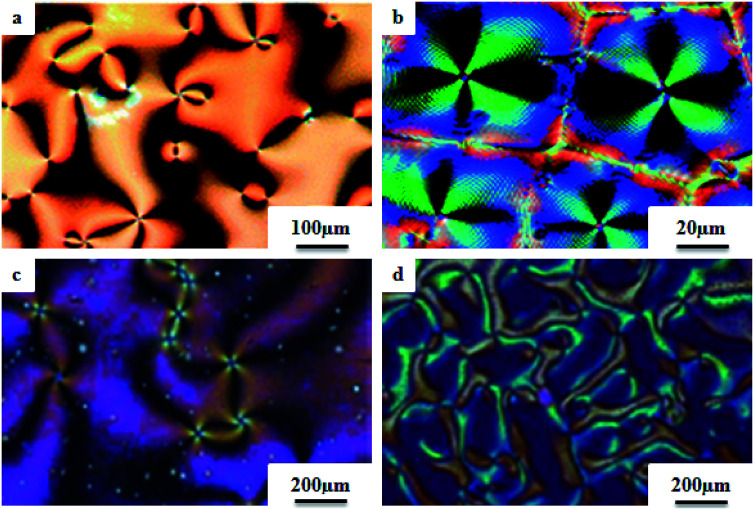
Textures of (a) nematic, (b) nematic*, (c) discotic nematic, and (d) columnar nematic phases observed by POM.^94,123^

**Fig. 25:**
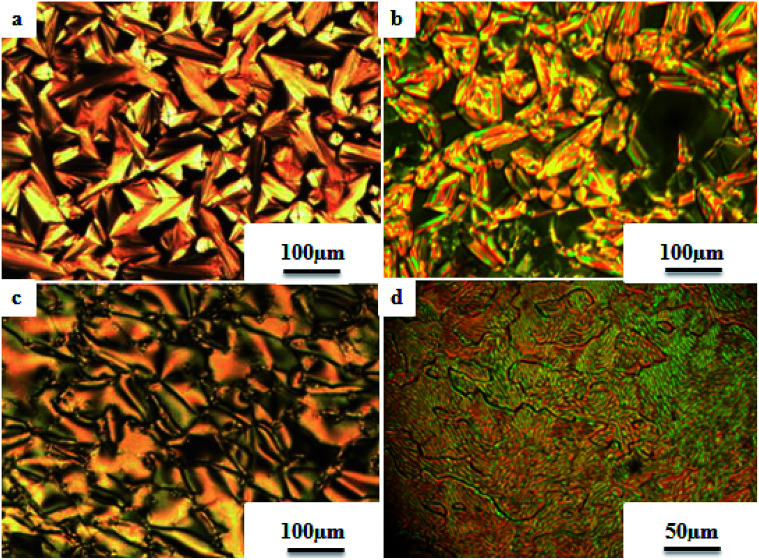
Textures of (a) smectic A, (b) smectic B, (c) smectic C, and (d) smectic X phases observed by POM.^61,124,125^

**Fig. 26:**
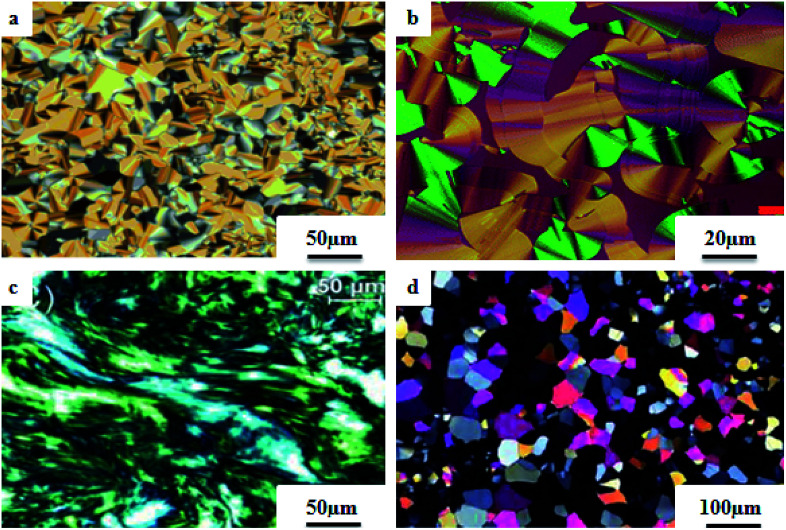
Textures of (a) columnar hexagonal, (b) columnar rectangular, (c) columnar orthorhombic, and (d) oblique columnar phases observed by POM.^90,97,126,127^

**Fig. 27:**
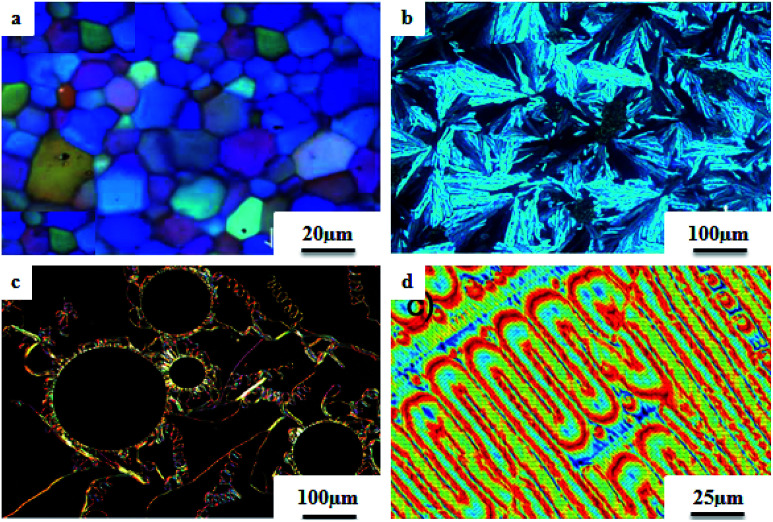
Textures of (a) BP*, (b) B1, (c) TGBA*, and (d) twist bend nematic phases observed by POM.^128–131^

The textures of various mesophases observed by POM for these pyridine-based compounds are shown in Fig. 24–27; also, the compounds which exhibit various mesophases and their temperature ranges are tabulated in Tables 1–7. As a whole, this paper provides a review of the latest advances in the area of pyridine-based liquid crystals with respect to the design of their molecular architectures and the establishment of suitable synthetic protocols, correlation of the shape and the type of mesophase, improvisation of the mesophase range, the roles of substituents, tuning of the melting and clearing temperatures with the aid of varying alkoxy chain lengths, preparation of hydrogen bonded complexes, photophysical properties, and device applications.

**Table 1:** List of compounds which exhibit nematic phases and their temperature ranges

Compound	Temperature range (°C)	Phase	Ref.
8a	131.0 → 144.2	Nematic	12
8b	90.6 → 133.8	Nematic	
8c	95.7 → 134.8	Nematic	
8d	87.2 → 128.7	Nematic	
8e	102.2 → 137.1	Nematic	
8f	94.9 → 133.9	Nematic	
8g	91.3 → 130.2	Nematic	
15a	113.0 → 120.0	Nematic*	13
15b	116.5 → 136.5	Nematic*	
23/24a	125.0 → 132.0	Nematic	17
23/24b	119.0 → 158.0	Nematic	
29a(4)	154.0 → 164.7	Nematic	21
29a(6)	111.5 → 156.4	Nematic	
29a(8)	109.3 → 149.1	Nematic	
29a(10)	127.6 → 144.8	Nematic	
29b(4)	107.3 → 115.4	Nematic	
29b(6)	110.9 → 121.8	Nematic	
29b(8)	98.3 → 114.4	Nematic	
29b(10)	96.9 → 114.6	Nematic	
29b(12)	96.1 → 104.1	Nematic	
40b	237.6 → 257.2	Nematic	22
40c	230.4 → 245.7	Nematic	
38b	203.0 → 237.1	Nematic	
38c	194.3 → 226.3	Nematic	
38d	196.2 → 229.7	Nematic	
38e	185.6 → 219.2	Nematic	
38f	187.3 → 220.5	Nematic	
38g	174.8 → 215.6	Nematic	
38h	179.8 → 220.1	Nematic	
57b,d/47a	186.9 → 154.9	Nematic	23
57c,d/47a	168.7 → 120.3	Nematic	
57b,d/47a*	177.6 → 123.8	Nematic*	
57c,d/47a*	157.5 → 82.1	Nematic*	
57b,d*/47a	147.9 → 126.3	Nematic*	
57c,d*/47a	91.3 → 73.5	Nematic*	
57b,d*/47a*	84.9 → 68.5	Nematic*	
57c,d*/47a*	64.3 → 42.3	Nematic*	
79c	112.7 → 115.7	Nematic	32
79d	104.9 → 109.6	Nematic	
792e	81.7 → 106.7	Nematic	
79f	82.3 → 103.8	Nematic	
79g	93.5 → 100.3	Nematic	
84b/85b	122.3 → 117.7	Nematic	37
84b/85f	108.4 → 106.7	Nematic*	
84c/85c	97.0 → 57.3	Nematic	
84d/85b	61.4 → 51.6	Nematic*	
84d/85c	88.6 → 41.9	Nematic*	
84d/85f	26.9 → 13.9	Nematic*	
84e/85c	107.5 → 76.3	Nematic	
84f/85b	132.8 → 91.7	Nematic*	
84f/85c	140.1 → 75.8	Nematic*	
84f/85d	78.3 → 69.7	Nematic*	
84f/85e	91.3 → 43	Nematic*	
84f/85f	111.1 → 81.8	Nematic*	
89a/90a	134.4 → 185.7	Nematic	40
89a/90b	135.0 → 183.0	Nematic	
89a/90c	140.1 → 190.7	Nematic	
89a/90d	137.5 → 180.0	Nematic	
89b/90a	143.8 → 184.4	Nematic	
89b/90b	143.2 → 180.1	Nematic	
89b/90c	136.7 → 180.6	Nematic	
89b/90d	135.7 → 173.2	Nematic	
89c/90a	138.8 → 204.2	Nematic	
89c/90b	140.1 → 196.3	Nematic	
89c/90c	140.0 → 198.2	Nematic	
89c/90d	140.8 → 193.2	Nematic	
89c/90a	139.5 → 194.4	Nematic	
89c/90b	138.2 → 191.9	Nematic	
89c/90c	140.4 → 190.6	Nematic	
89c/90d	140.6 → 187.0	Nematic	
109	80.7 → 48.3	Nematic	49
116	145.2 → 239.9	Nematic	
169a	50.3 → 41.2	Nematic	50
169b	80.7 → 48.3	Nematic	
169c	63.7 → 81.6	Nematic	
169d	77.1 → 85.9	Nematic	
174a	137.4 → 220.3	Nematic	
174b	145.2 → 239.9	Nematic	
174c	135.7 → 231.2	Nematic	
174d	134.9 → 228.0	Nematic	
177a	162.9 → 236.0	Nematic	
177b	160.9 → 238.8	Nematic	
177c	164.0 → 236.7	Nematic	
177d	164.3 → 238.0	Nematic	
126 + 50 wt% CL	119.0 → 117.0	Nematic	53
152a	133.3 → 186.3	Nematic	63
152b	135.3 → 156.0	Nematic	
152c	116.7 → 149.4	Nematic	
152d	131.0 → 144.9	Nematic	
152e	125.3 → 139.4	Nematic	
162a	128.0 → 141.0	Nematic	70
162b	134.0 → 139.0	Nematic	
163a	208.0 → 124.0	Nematic	
163b	172.0 → 201.0	Nematic	
162a/163a	108.0 → 192.0	Nematic	
162b/163a	114.0 → 186.0	Nematic	
162c/163a	114.0 → 180.0	Nematic	
162d/163a	116.0 → 178.0	Nematic	
162a/163b	120.0 → 198.0	Nematic	
162b/163b	103.0 → 193.0	Nematic	
162c/163b	109.0 → 188.0	Nematic	
162d/163b	110.0 → 189.0	Nematic	
186a	146.8 → 158.9	Nematic	75
186b	126.1 → 152.3	Nematic	
187a	86.2 → 109.4	Twist bend nematic	
187a	109.4 → 166.4	Nematic	
187b	93.5 → 98.0	Twist bend nematic	
187b	98.0 → 157.7	Nematic	
192a	86.0 → 77.0	Nematic	77
196a	115.7 → 128.3	Nematic	80
196b	109.6 → 128.7	Nematic	
196c	101.4 → 125.3	Nematic	
196d	117.9 → 133.7	Nematic	
196e	109.0 → 128.2	Nematic	
196f	122.2 → 138.8	Nematic	
208a	127.1 → 142.8	Nematic	82
219a	150.9 → 120.0	Columnar nematic	89
219b	152.8 → 128.2	Columnar nematic	
219c	147.2 → 103.4	Discotic nematic	
224/226a	93.6 → 84.7	Nematic	91
224/226b	91.4 → 71.4	Nematic	
224/226c	91.1 → 70.1	Nematic	
224/226d	91.9 → 74.5	Nematic	
224/226e	92.9 → 71.9	Nematic	
224/226f	91.9 → 74.5	Nematic	
225/226a	92.3 → 83.0	Nematic	
225/226b	90.7 → 73.5	Nematic	
225/226c	87.3 → 63.7	Nematic	
225/226d	91.0 → 67.1	Nematic	
225/226e	81.5 → 69.7	Nematic	
225/226f	92.5 → 82.1	Nematic	
224/227a	73.7 → 65.5	Nematic	
224/227b	76.4 → 67.8	Nematic	
224/227c	77.3 → 69.5	Nematic	
224/227d	80.0 → 68.9	Nematic	
224/227e	81.4 → 69.6	Nematic	
224/227f	84.8 → 71.5	Nematic	
225/227a	70.0 → 65.4	Nematic	
225/227b	74.5 → 63.6	Nematic	
225/227c	72.5 → 62.5	Nematic	
225/227d	76.3 → 68.4	Nematic	
225/227e	78.4 → 67.2	Nematic	
225/227f	82.8 → 71.5	Nematic	
238a/239a	138.0 → 241.2	Nematic	93
238a/239b	138.1 → 238.7	Nematic	
238a/239c	137.7 → 238.5	Nematic	
238a/239d	137.4 → 238.5	Nematic	
238a/239e	133.2 → 238.0	Nematic	
238a/239f	129.2 → 238.3	Nematic	
238b/239a	137.2 → 202.6	Nematic	
238b/239b	140.8 → 200.1	Nematic	
238b/239c	137.8 → 201.9	Nematic	
238b/239d	137.2 → 202.0	Nematic	
238b/239e	137.0 → 201.0	Nematic	
238b/239f	137.0 → 198.7	Nematic	
238c/239a	134.4 → 181.4	Nematic	
238c/239b	135.3 → 182.4	Nematic	
238c/239c	135.4 → 181.5	Nematic	
238c/239d	136.0 → 182.2	Nematic	
238c/239e	136.3 → 180.9	Nematic	
238c/239f	135.6 → 180.1	Nematic	
238d/239a	136.8 → 167.2	Nematic	
238d/239b	137.0 → 167.6	Nematic	
238d/239c	137.1 → 167.3	Nematic	
238d/239d	137.7 → 168.3	Nematic	
238d/239e	138.6 → 167.9	Nematic	
238d/239f	138.2 → 168.2	Nematic	
238e/239a	139.3 → 153.1	Nematic	
238e/239b	139.8 → 154.6	Nematic	
238e/239c	140.2 → 153.8	Nematic	
238e/239d	140.0 → 154.7	Nematic	
238e/239e	140.1 → 154.5	Nematic	
238e/239f	140.2 → 154.6	Nematic	
238f/239a	137.7 → 144.2	Nematic	
238f/239b	137.9 → 145.9	Nematic	
238f/239c	137.3 → 144.3	Nematic	
238f/239d	138.4 → 145.7	Nematic	
238f/239e	138.0 → 145.8	Nematic	
238f/239f	138.5 → 145.6	Nematic	
255/265	92.7 → 62.3	Nematic*	96
255/270	101.3 → 87.6	Nematic*	
278/288	146.9 → 96.6	Nematic*	97
278/286	145.2 → 88.1	Nematic*	
277/285	124.6 → 97.4	Nematic*	
277/284	122.6 → 97.4	Nematic*	
278/285	137.5 → 106.6	Nematic*	
278/284	148.3 → 86.8	Nematic*	
276/287	151.8 → 111.2	Nematic*	
345a	210.9 → 260.1	Nematic	108
345b	202.4 → 251.7	Nematic	
345c	208.3 → 255.8	Nematic	
345d	197.8 → 245.9	Nematic	
345e	201.1 → 248.3	Nematic	
345f	192.4 → 238.4	Nematic	
346a	207.3 → 254.8	Nematic	
346b	192.4 → 238.4	Nematic	
346c	203.0 → 246.0	Nematic	
346d	191.6 → 224.4	Nematic	
346e	194.0 → 229.7	Nematic	
346f	187.5 → 218.0	Nematic	

**Table 2:** List of compounds which exhibit smectic phases and their temperature ranges

Compound	Temperature range (°C)	Phase	Ref.
7a	108.0 → 136.2	Smectic A	12
7b	103.3 → 136.5	Smectic A	
7c	94.8 → 135.5	Smectic A	
7d	86.7 → 139.5	Smectic A	
7e	90.2 → 140.9	Smectic A	
7f	94.5 → 143.6	Smectic A	
7g	91.3 → 141.2	Smectic A	
15a	42.7 → 112.2	Smectic A	13
15b	74.2 → 115.3	Smectic A	
19	117.0 → 134.0	Smectic A	14
22c	123.6 → 135.5	Smectic A	15
22d	119.6 → 135.0	Smectic A	
22e	117.2 → 133.8	Smectic A	
22f	75.1 → 108.8	Smectic A	
22g	100.3 → 116.0	Smectic A	
22h	100.3 → 107.5	Smectic A	
22i	70.5 → 158.3	Smectic A	
22j	94.8 → 107.3	Smectic A	
25/24a	108.0 → 117.0	Smectic A	20
25/24b	117.0 → 135.0	Smectic A	
40d	231.2 → 249.5	Smectic A	22
40e	220.5 → 241.4	Smectic A	
40f	223.7 → 243.3	Smectic A	
40g	212.8 → 234.9	Smectic A	
40h	192.0 → 236.4	Smectic A	
57b,d/47a	154.9 → 84.8	Smectic A	23
57c,d/47a	120.3 → 74.9	Smectic A	
57b,d/47a*	123.8 → 70.1	Smectic A	
57c,d/47a*	82.1 → 60.8	Smectic A	
57b,d*/47a	126.3 → 64.9	Smectic A	
57c,d*/47a	73.5 → 35.2	Smectic A	
57b,d*/47a*	68.5 → 41.5	Smectic A	
56a(8)	104.2 → 109.3	Smectic C	30
56a(10)	101.6 → 127.6	Smectic C	
56a(12)	102.7 → 137.2	Smectic C	
56a(16)	106.9 → 134.4	Smectic C	
56b(8)	75.7 → 98.3	Smectic C	
56b(10)	78.1 → 96.9	Smectic C	
56b(12)	89.1 → 96.1	Smectic C	
82b	80.0 → 52.0	Smectic A	35
82c	82.0 → 74.0	Smectic A	
82d	81.0 → 65.0	Smectic A	
82e	92.0 → 78.0	Smectic A	
84b/85a	60.8 → 37.8	Smectic A	37
84b/85b	117.7 → 93.6	Smectic A	
84b/85c	134.0 → 78.6	Smectic A	
84b/85d	84.4 → 47.2	Smectic A	
84b/85e	91.6 → 55.2	Smectic A	
84b/85f	106.7 → 64.2	Smectic A	
84f/85a	68.7 → 11.6	Smectic A	
89a/90a	108.8 → 134.4	Smectic C	40
89a/90b	121.3 → 135.0	Smectic C	
89a/90c	123.8 → 140.1	Smectic C	
89a/90d	121.9 → 137.5	Smectic C	
89b/90a	131.3 → 143.8	Smectic C	
89b/90b	129.4 → 143.2	Smectic C	
89b/90c	128.2 → 136.7	Smectic C	
89b/90d	123.8 → 135.7	Smectic C	
89c/90a	118.8 → 138.8	Smectic C	
89c/90b	128.2 → 140.1	Smectic C	
89c/90c	131.9 → 140.0	Smectic C	
89c/90d	128.8 → 140.8	Smectic C	
89c/90a	129.4 → 139.5	Smectic C	
89c/90b	128.2 → 138.2	Smectic C	
89c/90c	136.9 → 140.4	Smectic C	
89c/90d	129.4 → 140.6	Smectic C	
97b	50.8 → 239.0	Smectic A	44
98b	91.0 → 133.0	Smectic A	
98c	107 → 157.0	Smectic A	
98d	101.5 → 202.6	Smectic A	
99a	88.3 → 170.1	Smectic A	
99b	83.7 → 216.8	Smectic A	
99c	86.4 → 252.9	Smectic A	
99d	88.6 → 272.9	Smectic A	
100a	124.4 → 105.1	Smectic A	
100b	124.3 → 167.2	Smectic A	
100c	120.9 → 221.3	Smectic A	
100d	121.8 → 228.3	Smectic A	
101b	80.1 → 108.8	Smectic A	
102c	45.4 → 34.4	Smectic A	
102d	60.5 → 77.8	Smectic A	
103b	122.6 → 199.0	Smectic A	
104b	29.6 → 222.8	Smectic A	
126	140.0 → 119.0	Smectic A	53
126	119.0 → 110.0	Smectic C	
126	80.0 → 62.0	Smectic X_2_	
126	62.0 → -4.0	Smectic X_1_	
126 + 50 wt% CL	117.0 → 59.0	Smectic A	
126 + 50 wt% CL	59.0 → -15.0	Smectic B	
135a	112.0 → 217.0	Smectic A	59
135b	113.1 → 256.0	Smectic A	
135c	88.7 → 275.0	Smectic A	
136c	140.4 → 153.8	Smectic X	
136c	153.8 → 336.0	Smectic A	
137c	162.0 → 312.0	Smectic A	
138c	131.7 → 337.0	Smectic A	
139a	104.3 → 254.3	Smectic A	
139b	104.5 → 304.8	Smectic A	
139c	103.8 → 328.0	Smectic A	
140a	143.7 → 216.7	Smectic A	
140b	156.9 → 220.2	Smectic A	
140c	138.7 → 203.7	Smectic A	
141c	118.1 → 153.9	Smectic X	
141c	153.9 → 274.0	Smectic A	
144a/145a	48.1 → 43.2	Smectic B	61
144a/145b	56.8 → 47.7	Smectic B	
144a/145c	58.4 → 55.3	Smectic B	
144a/145d	62.2 → 58.4	Smectic B	
144a/145e	63.8 → 53.1	Smectic B	
144a/145f	65.1 → 53.7	Smectic B	
144a/145g	65.6 → 52.7	Smectic B	
144b/145a	53.8 → 51.9	Smectic B	
144b/145b	57.9 → 43.3	Smectic B	
144b/145c	58.8 → 50.7	Smectic B	
144b/145d	63.8 → 59.6	Smectic B	
144b/145e	65.2 → 59.9	Smectic B	
144b/145f	70.7 → 67.8	Smectic B	
144b/145g	72.3 → 67.4	Smectic B	
152d	101.8 → 131.0	Smectic A	63
152e	94.5 → 138.4	Smectic A	
152f	90.9 → 142.2	Smectic A	
152g	99.2 → 143.0	Smectic A	
152h	102.1 → 142.2	Smectic A	
152i	104.4 → 137.3	Smectic A	
159c	46.5 → 44.8	Smectic A	68
159d	43.5 → 38.0	Smectic A	
159e	45.5 → 30.4	Smectic A	
159f	49.8 → 38.1	Smectic A	
159g	43.8 → 42.8	Smectic A	
159h	49.0 → 49.2	Smectic A	
162a	112.0 → 128.0	Smectic A	70
162b	94.0 → 134.0	Smectic A	
162c	96.0 → 134.0	Smectic A	
162d	97.0 → 133.0	Smectic A	
187a	121.9 → 86.2	Smectic X	75
187b	112.0 → 85.5	Smectic X	
187b	85.5 → 93.5	Smectic A	
224/229a	68.1 → 52.82	Smectic	91
224/229b	76.4 → 55.81	Smectic	
238a/239a	117.8 → 138.0	Smectic C	93
238a/239b	99.1 → 138.1	Smectic C	
238a/239c	92.2 → 137.7	Smectic C	
238a/239d	84.5 → 137.4	Smectic C	
238a/239e	88.6 → 133.2	Smectic C	
238a/239f	91.3 → 129.2	Smectic C	
238b/239a	119.1 → 137.2	Smectic C	
238b/239b	98.8 → 140.8	Smectic C	
238b/239c	91.6 → 137.8	Smectic C	
238b/239d	86.1 → 137.2	Smectic C	
238b/239e	93.2 → 137.0	Smectic C	
238b/239f	91.8 → 137.0	Smectic C	
238c/239a	118.0 → 134.4	Smectic C	
238c/239b	99.7 → 135.3	Smectic C	
238c/239c	90.8 → 135.4	Smectic C	
238c/239d	86.2 → 136.0	Smectic C	
238c/239e	89.8 → 136.3	Smectic C	
238c/239f	91.6 → 135.6	Smectic C	
238d/239a	118.1 → 136.8	Smectic C	
238d/239b	97.3 → 137.0	Smectic C	
238d/239c	92.8 → 137.1	Smectic C	
238d/239d	86.6 → 137.7	Smectic C	
238d/239e	90.0 → 138.6	Smectic C	
238d/239f	92.2 → 138.2	Smectic C	
238e/239a	118.1 → 139.3	Smectic C	
238e/239b	99.1 → 139.8	Smectic C	
238e/239c	92.3 → 140.2	Smectic C	
238e/239d	86.4 → 140.0	Smectic C	
238e/239e	87.0 → 140.1	Smectic C	
238e/239f	92.4 → 140.2	Smectic C	
238f/239a	118.4 → 137.7	Smectic C	
238f/239b	100.1 → 137.9	Smectic C	
238f/239c	91.8 → 137.3	Smectic C	
238f/239d	85.8 → 138.4	Smectic C	
238f/239e	89.4 → 138.0	Smectic C	
238f/239f	92.4 → 138.5	Smectic C	
278/288	96.6 → 41.3	Smectic A	97
278/286	88.1 → 44.9	Smectic A	
277/285	97.4 → 56.8	Smectic A	
277/284	97.4 → 64.6	Smectic A	
278/285	106.6 → 68.2	Smectic A	
278/284	86.8 → 60.6	Smectic A	
276/287	111.2 → 86.2	Smectic A	
332	100.0 → 135.0	Smectic C	106
*Z*-375	50.0 → 78.0	Smectic	115

**Table 3:** List of compounds which exhibit columnar phases and their temperature ranges

Compound	Temperature range (°C)	Phase	Ref.
328a	124.0 → 187.0	Achiral *Ia*3̄*d* phase	41
328a	187.0 → 196.0	Chiral *I*432 phase	
328b	128.0 → 201.0	Chiral *I*432 phase	
328c	123.0 → 191.0	Chiral *I*432 phase	
328d	92.0 → 184.0	Chiral *I*432 phase	
196f	122.2 → 138.8	Columnar rectangular	80
196g	rt → 107.2	Columnar rectangular	
196h	rt → 113.6	Columnar rectangular	
196i	rt → 90.84	Columnar rectangular	
196j	100.9 → 114.9	Columnar rectangular	
196k	129.2 → 137.8	Columnar rectangular	
196l	104.7 → 117.9	Columnar rectangular	
196m	rt → 93.7	Columnar rectangular	
204a	rt → 110.0	Columnar hexagonal	81
204b	rt → 87.2	Columnar hexagonal	
204c	rt → 71.6	Columnar hexagonal	
204d	rt → 74.3	Columnar hexagonal	
204e	rt → 81.3	Columnar hexagonal	
208b	105.5 → 125.2	Columnar orthorhombic	82
208c	105.1 → 121.4	Columnar orthorhombic	
208d	102.6 → 120.5	Columnar orthorhombic	
208e	101.6 → 118.2	Columnar orthorhombic	
208f	99.3 → 113.3	Columnar orthorhombic	
211a	123.0 → 57.0	Columnar rectangular	83
211b	108.0 → 91.0	Columnar hexagonal	
211c	rt → 66.0	Columnar hexagonal	
215c	35.7 → 2.4	Columnar hexagonal	85
216c	35.5 → -18.0	Columnar hexagonal	
236a	89.48 → 153.02	Oblique columnar	92
236b	88.74 → 135.77	Ordered columnar hexagonal	
236b	135.77 → 149.14	Disordered columnar hexagonal	
236c	74.95 → 120.36	Ordered columnar hexagonal	
236c	120.36 → 140.31	Disordered columnar hexagonal	
236d	112.8 → 50.3	Ordered columnar hexagonal	
311c	75.8 → 45.3	Columnar rectangular 1	102
311c	45.3 → rt	Columnar rectangular 2	
311d	92.2 → 42.1	Columnar hexagonal	
312b	126.7 → 99.1	Columnar rectangular	
312c	95.6 → 70.0	Columnar hexagonal	
312c	70.0 → 43.6	Columnar rectangular 1	
312c	43.6 → rt	Columnar rectangular 2	
312d	119.7 → 73.0	Columnar rectangular	
312d	73.0 → 57.3	Columnar rectangular 1	
312d	57.3 → rt	Columnar rectangular	

**Table 4:** List of compounds which exhibit TGBA* (twisted-grain-boundary) phase and their temperature ranges

Compound	Temperature range (°C)	Phase	Ref.
15a	112.2 → 113	TGBA*	13
15b	115.3 → 116.5	TGBA*	
84f/85b	91.7 → 61.8	TGBA*	37
84f/85c	75.8 → 67.9	TGBA*	
84f/85d	69.7 → 17.7	TGBA*	
84f/85f	81.8 → 22.9	TGBA*	

**Table 5:** List of compounds which exhibit blue phase (BP*) and their temperature ranges

Compound	Temperature range (°C)	Phase	Ref.
15b	136.5 → 137.5	BP*	13
57b,d*/47a	150.3 → 147.9	BP*	23
57c,d*/47a	96.9 → 91.3	BP*	
57b,d*/47a*	89.6 → 84.9	BP*	
57c,d*/47a*	70.3 → 64.3	BP*	
278/285	151.2 → 137.5	BP*	97

**Table 6:** List of compounds which exhibit B1 phase and their temperature ranges

Compound	Temperature range (°C)	Phase	Ref.
306b	102.0 → 162.3	B1	98
306c	168.5 → 179.3	B1	

**Table 7:** List of other compounds which exhibit different phases and their approximate temperature ranges

Compound	Temperature range (°C)	Phases	Ref.
31a(*n*)	90.0 < phases < 190.0	Nematic and smectic A	21
31b(*n*)	95.0 < phases < 170.0	Nematic	
31c(*n*)	110.0 < phases < 175.0	Nematic	
30a(*n*)	45.0 < phases < 100.0	Nematic	
30b(*n*)	35.0 < phases < 90.0	Nematic	
58b-eX_2_	0.0 < phases < 185.0	Smectic A, ordered columnar, disordered columnar, lamellar columnar	25
59c/60a–e	80.0 < phases < 125.0	Nematic and smectic A	26
59b/60a–e	90.0 < phases < 125.0	Nematic and smectic A	
59a/60a–e	85.0 < phases < 125.0	Nematic and smectic A	
63a–c	120.0 < phases < 185.0	Smectic C, lamellar and ordered columnar	28
64a–c	180.0 < phases < 280.0	Nematic, disordered and lamellar columnar	
67–68	20.0 < phases < 70.0	Nematic*	30
69–72	65.0 < phases < 240.0	Nematic* and smectic A	
73–74	110.0 < phases < 230.0	Nematic* and smectic A	
86/[87 + (0–100%) 88]	145.0 < phases < 180.0	Nematic and smectic C	38
127/129e	115.0 < phases < 135.0	Smectic A	57
128a/129a–c	60.0 < phases < 100.0	Nematic	
128a/129d–e	65.0 < phases < 130.0	Smectic A	
128b/129a–b	60.0 < phases < 110.0	Nematic	
128b/129c–e	60.0 < phases < 120.0	smectic A	
222/223a–h	60.0 < phase < 100.0	Nematic	90
268/265 (0–100 mol% of 178)	40.0 < phases < 150.0	Nematic* and BP*	96
269/265 (0–100 mol% of 178)	60.0 < phases < 150.0	Nematic* and BP*	
255/265 (0–100 mol% of 178)	60.0 < phases < 150.0	Nematic* and BP*	
272/265 (0–100 mol% of 178)	80.0 < phases < 150.0	Nematic* and BP*	
315/322e	45.0 < phases < 55.0	Nematic and smectic C	105
317/322d–g	65.0 < phases < 95.0	Smectic	
318/322e–g	25.0 < phases < 55.0	Nematic	
321/322d–g	60.0 < phases < 95.0	Nematic	
335	40.0 < phases < 150.0	Nematic and smectic	107

## 4. Summary and conclusions

In this review article, prominence has been provided to the available synthetic routes and thermotropic properties of various pyridine-based molecules. In addition, their photo-isomerisation, opto-electric properties, blue phase ranges and structure–property relationships have been explained in some cases. Most of the molecules with short chain lengths exhibit nematic phase, whereas smectic A phase is observed for molecules bearing longer chain lengths. This is because the ratio of lateral to terminal attraction between the molecules increases as the chain length increases. As the chain length increases, the probability of layer arrangement during the melting process (crystal–liquid crystalline transition) increases due to the weakened terminal attractions. Further, it has been noted that the incorporation of a pyridine system into a molecular architecture not only alters its mesogenic behavior but also modifies its electronic and dielectric properties. Therefore, these molecules are currently gaining a wide range of applications in the fields of photovoltaic devices, organic LEDs, and field effect transistors. Moreover, some new generation batteries have started using ionic liquid crystals as electrolytes. Furthermore, molecules possessing azo linkages exhibit phase transitions upon UV light irradiation. Generally, this phase transition is because of photoisomerisation of azo compounds from *trans* to *cis* configuration as the bent-shaped isomers destabilize the liquid crystal phases. In these molecules, mesogenic textures emerge immediately after stopping the UV irradiation, and the textures are fully retrieved within a few seconds. Hence, these molecules are more suitable for photonic applications. As a whole, this article will help researchers to establish synthetic strategies to prepare new pyridine-based mesogenic molecules.

## 5. Future prospects

The exigency of advancing technological developments is currently increasing. To meet these demands, the sustained design and preparation of new mesogenic molecules is highly desirable. One great challenge in the future is to synthesize these molecules in a greener way, *i.e.* using ionic liquids, ultra-sonication, *etc.* Further, the design of new molecular entities encompassing both mesomorphic and luminescence properties in the future will establish these materials as excellent contenders for opto-electronic applications. Furthermore, the design of pyridine-based luminescent mesomorphic molecules may be an innovative strategy to enlarge the viewing angles of twisted nematic liquid crystal displays and also to overcome limitations such as loss of contrast at high temperature and high heat generation due to high consumption of electricity. Finally, the refinement of this pyridine platform to tailor the exigency of various technological devices based on optical and electrical properties can be envisioned.

## Conflicts of interest

There are no conflicts to declare.

## Supplementary Material
